# Synthesis of 1-indolyl-3,5,8-substituted γ-carbolines: one-pot solvent-free protocol and biological evaluation

**DOI:** 10.3762/bjoc.17.101

**Published:** 2021-06-17

**Authors:** Premansh Dudhe, Mena Asha Krishnan, Kratika Yadav, Diptendu Roy, Krishnan Venkatasubbaiah, Biswarup Pathak, Venkatesh Chelvam

**Affiliations:** 1 Department of Chemistry, Indian Institute of Technology Indore, Khandwa Road, Simrol, Indore-453 552, India; 2 Department of Biosciences and Biomedical Engineering, Indian Institute of Technology Indore, Khandwa Road, Simrol, Indore-453 552, India,; 3 School of Chemical Sciences, National Institute of Science Education and Research, Bhubaneswar-752 050, Odisha, India

**Keywords:** γ-carboline, cascade reaction, cell uptake, cytotoxicity, fluorescence

## Abstract

1,5**-**Disubstituted indole-2-carboxaldehyde derivatives **1a**–**h** and glycine alkyl esters **2a**–**c** are shown to undergo a novel cascade imination-heterocylization in the presence of the organic base DIPEA to provide 1-indolyl-3,5,8-substituted γ-carbolines **3aa**–**ea** in good yields. The γ-carbolines are fluorescent and exhibit anticancer activities against cervical, lung, breast, skin, and kidney cancer cells.

## Introduction

**Figure 1:**
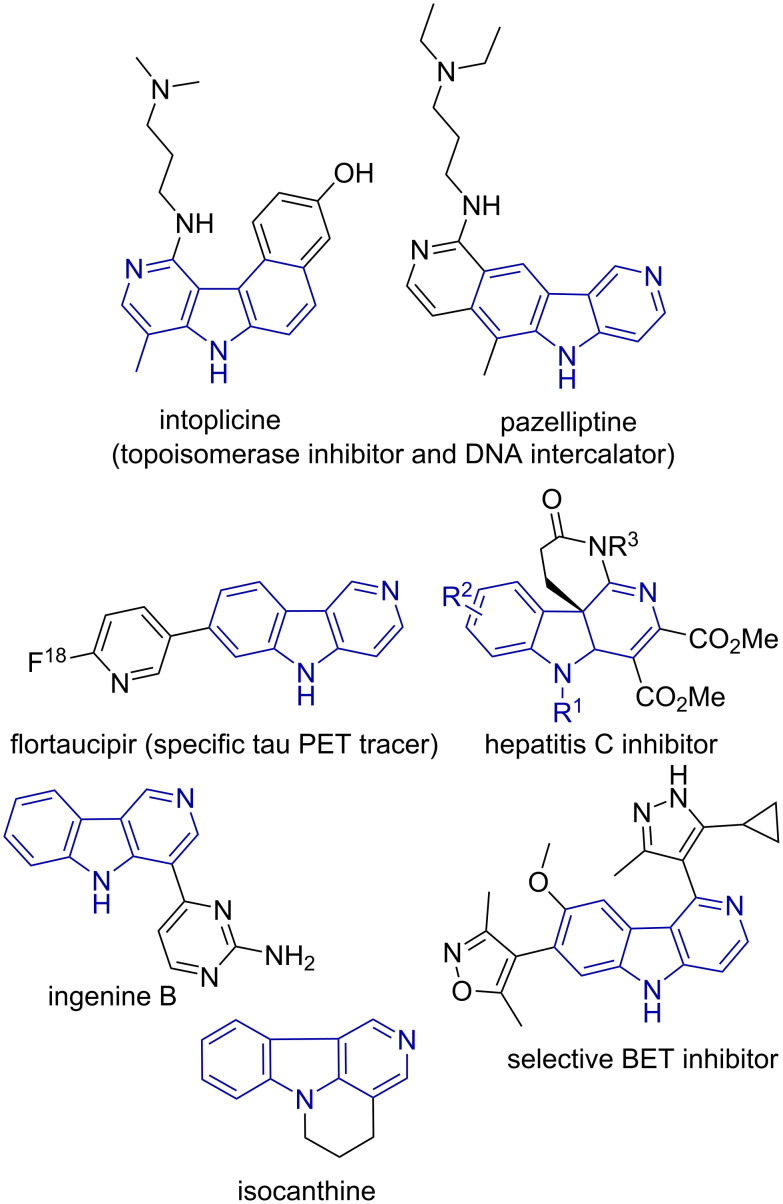
Selected examples of compounds containing the γ-carboline core.

Carbolines are privileged aza-heterocycles found in the core of several natural and synthetic compounds and are known for their biological applications. Among the four different isomers, 9*H*-pyrido[3,4-*b*]indole (β-carboline) is the most naturally abundant, present for instance, in the alkaloid harmine, a well-known selective inhibitor of monoamine oxidase-A (MAO-A) [1]. On the contrary, 5*H*-pyrido[4,3-*b*]indoles (γ-carbolines) are comparatively less examined, although these heterocycles have shown promising biological activities in preclinical and clinical studies (Figure 1) [2–6].

The pyrimidine-γ-carboline alkaloid ingenine B (isolated from an Indonesian sponge) exhibits a pronounced cytotoxicity against a murine lymphoma cell line [7] and several isocanthine analogs are effective against cervical cancer (HeLa cells) [8]. Moreover, γ-carbolines are known for their well-established DNA intercalating [9] and anti-Alzheimer [10] properties.

**Scheme 1:**
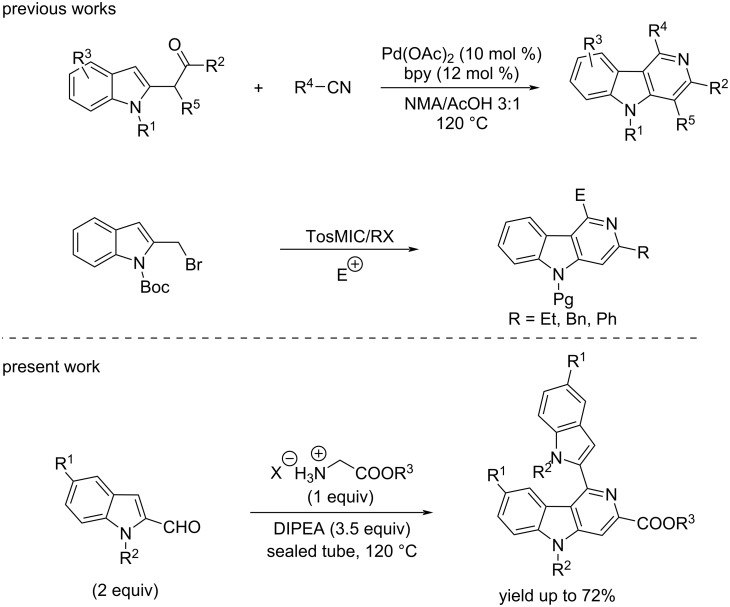
The synthetic strategy of present work in comparison with previous reports.

The classical Graebe–Ullmann synthesis [11] of γ-carbolines, one of the very early protocols in the domain, is based on the thermal decomposition of *N-*pyridylbenzotriazoles. Later, the reaction conditions were modified to make this reaction more versatile and operationally simple such as by the use of microwave irradiation [12]. Meanwhile, the Fischer indole synthesis was successfully extended for the synthesis of significant biologically active tetrahydro-γ-carboline derivatives [13–14]. A systematic assessment of the above Graebe–Ullmann and Fischer synthesis protocols revealed that these reactions are associated with i) low product yield, ii) limited scope including the use of a very specific set of substrates, and iii) involvement of extreme thermal conditions with the use of corrosive reagents. Much later, Larock and co-workers developed a Pd/Cu-catalyzed imino-annulation of internal alkynes [15], which paved the way for transition-metal-catalyzed cyclizations as easy access to these scaffolds. Notably, the gold-catalyzed tandem cycloisomerization/Pictet–Spengler cyclization of 2-(4-aminobut-1-yn-1-yl)aniline [16], the Ru and Rh-catalyzed [2 + 2 + 2] cycloadditions of yne-ynamides [17], and the Pd-catalyzed tandem coupling-cyclization [18] are significant works in the area (Scheme 1). However, the use of toxic and expensive metal catalysts has limited their development as environment-friendly synthetic protocols. More recently, an acid-catalyzed cyclization of α-indol-2-ylmethyl TosMIC (tosylmethyl isocyanide) derivatives to synthesize heterocycles [19] has been thoroughly studied (Scheme 1), including the synthesis of the carboline alkaloid ingenine B [20]. The iodine-catalyzed [3 + 3] cycloaddition of indolyl alcohol to enaminones [21] and the thiourea-catalyzed iso-Pictet–Spengler reaction of isotryptamine with aldehydes [22], are some noteworthy contributions to the field.

A cascade or domino reaction is an interesting approach for the design of efficient one-step transformations for complex molecule synthesis [23–24]. Employing domino reactions to simplify cumbersome industrial processes can afford complex pharmaceutical products in an economical and environment-friendly manner [25]. Easy workup procedures and single-step purification reduce the efforts in the synthesis of complex molecular architectures. Therefore, cascade reactions are essential in synthetic organic chemistry, even with moderate yields [26]. Recently, such reactions have claimed their much deserving place in drug design and natural product synthesis [27]. In the literature, there is only a limited number of direct synthetic procedures to prepare γ-carbolines till date [28], and this gives a cutting edge advantage to our new protocol wherein a solvent and metal-free direct access to the γ-carboline core from substituted indole-2-aldehyes and glycine ester salts has been discovered.

## Results and Discussion

### Optimization of reaction conditions and chemical synthesis

The base-catalyzed imination of aromatic aldehydes is a valuable method in organic synthesis to synthesize a variety of heterocyclic building blocks [29]. Among all the reported iminoesters, alkyl *N-*arylideneglycinates have attracted much attention in recent years. For instance, the metal-catalyzed asymmetric [3 + 2] cycloaddition of ethyl *N-*benzylideneglycinates with electron-deficient alkenes has been reported to yield substituted pyrrolidines [30].

Recently, we reported the synthesis of substituted pyrrole-2-aldehydes to 5-azaindole transformation during a base-catalyzed imination reaction [31]. However, we envisioned that our methodology might be strategically applied towards the synthesis of substituted γ-carbolines as a C-3 nucleophilic attack is more favored in indoles than in pyrroles. Herein, we report an interesting observation for conversion of substituted indole-2-aldehydes **1** to 1-indolyl-3,5,8-substituted γ-carbolines **3** by a cascade imination-heterocylization pathway when treated with the salt glycine methyl ester hydrochloride (**2a**) in the presence of a base.

Earlier in the literature, it was reported that 1*H*-indole-2-carbaldehyde derivatives underwent condensation with *N-*arylideneglycinate to form pyrimidoindole derivatives [32]. However, when 1-methyl-1*H*-indole-2-carbaldehyde (**1a**) and glycine methyl ester hydrochloride salt (**2a**) were reacted in the presence of DIPEA (Hünig’s base) at room temperature in a non-polar solvent such as toluene, only marginal amounts of the corresponding imine were observed, that could not be isolated (Table 1, entry 1). When the reaction mixture was further heated to reflux for 16 h, only traces of 1-indolyl 3,5,8-substituted γ-carboline **3aa** were formed that were still insufficient for complete characterization. Intending to improve the yield of **3aa**, we screened various solvents, non-nucleophilic organic bases such as triethylamine and DBU, and several inorganic bases like K_2_CO_3_, Cs_2_CO_3,_ and NaH (Table 1, entries 2–6).

**Table 1:** Optimization of the reaction conditions for the transformation of 1-methylindole-2-carbaldeyde (**1a**) to γ-carboline **3aa**.

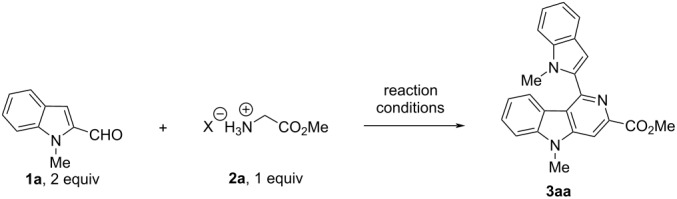						

entry	mmol of **1a** (conc.)	mmol of **2a** (conc.)	base (3.5 equiv, no of mmol, conc.)	solvent (5 mL)	temp.	yield

1	0.62 mmol (0.12 M)	0.31 mmol (0.06 M)	DIPEA (1.09 mmol, 0.21 M)	toluene^a^	rt to reflux	trace
2	0.62 mmol (0.12 M)	0.31 mmol (0.06 M)	Et_3_N (1.09 mmol, 0.21 M)	toluene^a^	rt to reflux	no product
3	0.62 mmol (0.12 M)	0.31 mmol (0.06 M)	DBU (1.09 mmol, 0.21 M)	toluene^a^	rt to reflux	trace
4	0.62 mmol (0.12 M)	0.31 mmol (0.06 M)	K_2_CO_3_ (1.09 mmol, 0.21 M)	Et_2_O^a^	rt to reflux	no product
5	0.62 mmol (0.12 M)	0.31 mmol (0.06 M)	Cs_2_CO_3_ (1.09 mmol, 0.21 M)	DMF^a^	rt to reflux	no product
6	0.62 mmol (0.12 M)	0.31 mmol (0.06 M)	NaH (1.09 mmol, 0.21 M)	THF^a^	rt to reflux	no product
7	0.62 mmol	0.31 mmol	DIPEA^b^	–	120 °C	70%

^a^Reactions were monitored by TLC for 3 h at room temperature followed by reflux for 16 h in the appropriate solvent; ^b^solvent-free reaction carried out in a 25 mL borosilicate sealed tube in a preheated oil bath in an air atmosphere at 120 °C.

After systematic screening of several reaction conditions, we found that heating at 120 °C of a neat mixture consisting of 1-methyl-1*H*-indole-2-carbaldehyde (**1a**, 2.0 equiv), glycine methyl ester HCl salt (**2a**, 1.0 equiv), and DIPEA (3.5 equiv) in a sealed tube for 6 h, led to the isolation of γ-carboline **3aa** in 70% yield (Table 1, entry 7). The product **3aa** was subsequently characterized by various spectroscopic techniques.

With the initial success at hand, the reaction was found to be equally effective with various glycine alkyl ester HCl salts **2a–c** but failed to result in the formation of 1-indolyl-3-cyano-5-methyl γ-carboline derivative **3ad** when 2-aminoacetonitrile **2d** was utilized as the condensation component. Then, a range of 1- and 1,5-disubstituted indole-2-carboxaldehyde derivatives **1a–h** was synthesized (for details see Supporting Information File 1) to evaluate the scope of the reaction further.

**Scheme 2:**
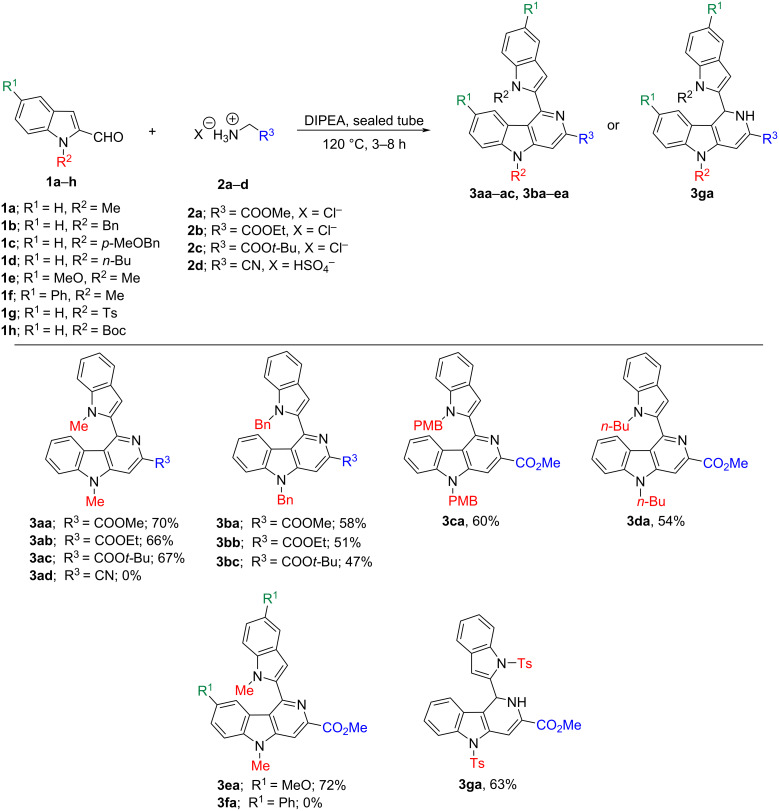
Series of synthesized 1-indolyl-3,5,8-substituted γ-carboline **3aa–ac**, **3ba-ea** and 1-indolyl-1,2-dihydro-3,5-substituted γ-carboline **3ga** derivatives.

**Figure 2:**
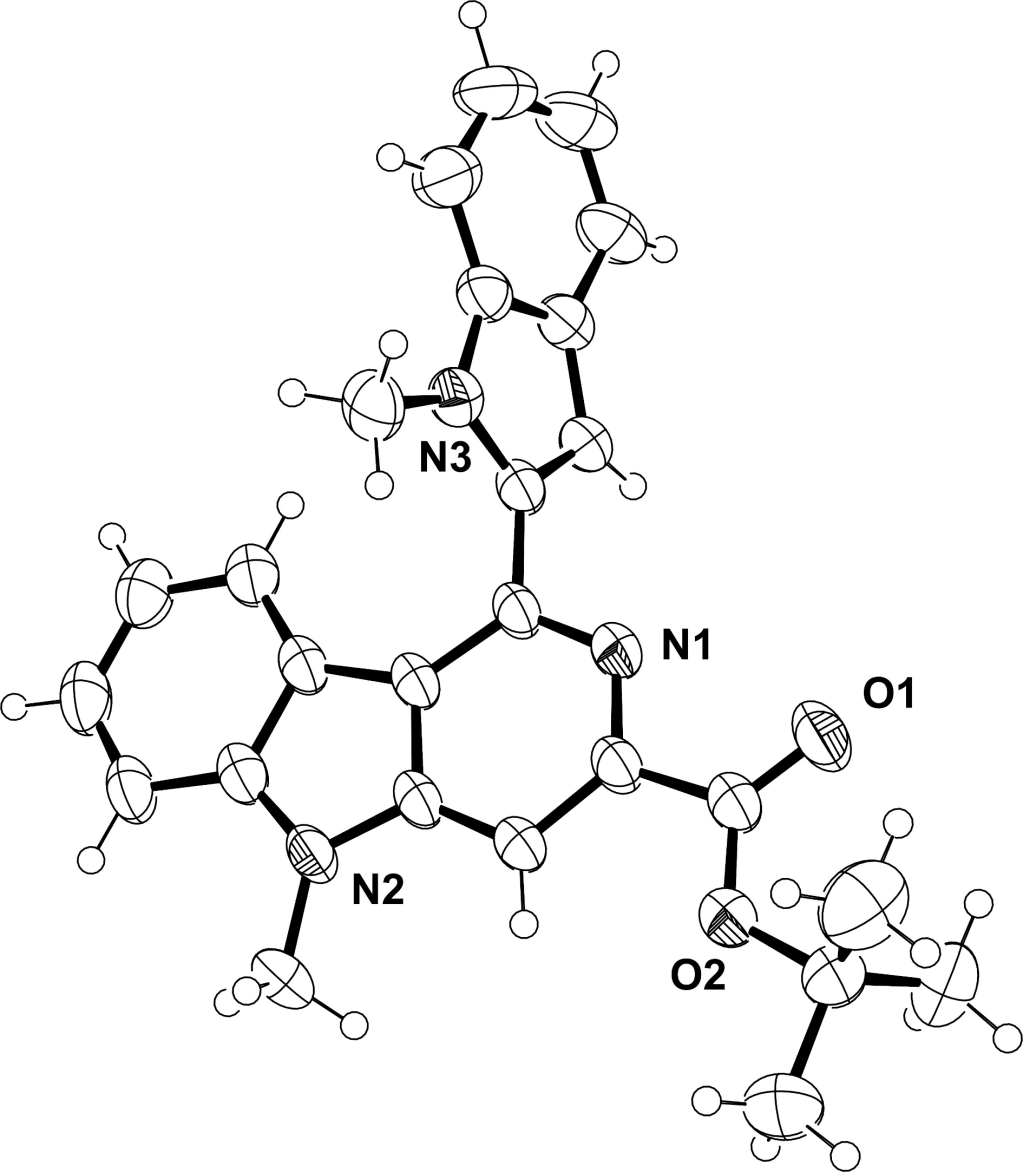
Single-crystal XRD structure of **3ac** (CCDC: 1897787).

Indole-2-carbaldehyde derivatives with electron-donating substituents in the 1-position of the indole ring system **1a–e** were transformed in moderate to good yields into their corresponding γ-carboline derivatives **3aa–ac** and **3ba–ea** due to an enhanced C-3 nucleophilicity of the indole nucleus (Scheme 2). The formation of the corresponding γ-carboline products was confirmed unequivocally by single-crystal X-ray diffraction analysis of **3ac** (Figure 2).

The presence of two substituents in the 1,5-position of indole-2-carbaldehyde substrates such as **1e** (5-methoxy-1-methyl-1*H*-indole-2-carbaldehyde) and **1f** (1-methyl-5-phenyl-1*H*-indole-2-carbaldehyde) influenced the outcome of the heterocylization reaction in different ways. For instance, 1-methyl-5-methoxy-substituted compound **1e** was successfully transformed into 1-indolyl-3-carbomethoxy-5-methyl-8-methoxy γ-carboline (**3ea**) in 72% yield, whereas the 1-methoxy-5-phenyl-substituted indolecarbaldehyde **1f** remained unreacted under the optimized reaction conditions and did not yield the expected 5-methyl-1-(1-methyl-5-phenyl-1*H*-indol-2-yl)-3-carbomethoxy-8-phenyl γ-carboline derivative **3fa**. The reason for this remains unclear but is likely due to the electron-withdrawing nature of the phenyl substituent at the 5-position in substrate **1f**. Indole substrates with a weak electron-withdrawing substituent in the 1-position such as *N-*tosyl in 1-tosyl-1*H*-indole-2-carbaldehyde (**1g**) did not affect the reaction course. The substrate was smoothly transformed into the corresponding 1-indolyl-3,5-disubstituted 1,2-dihydro-γ-carboline derivative **3ga** instead of completely aromatized γ-carboline when heated at 120 °C with glycine methyl ester hydrochloride and DIPEA for 8 h in a sealed tube. However, electron-withdrawing 1-substituents such as an *N-*Boc group in 1-*tert*-butyloxycarbonyl-1*H*-indole-2-carbaldehyde (**1h**), impeded the conversion to γ-carboline **3ha** (structure not shown) due to probable decomposition and decrease in nucleophilicity at the 3-position in substrate **1h**.

### Plausible mechanism for the formation of γ-carbolines

**Scheme 3:**
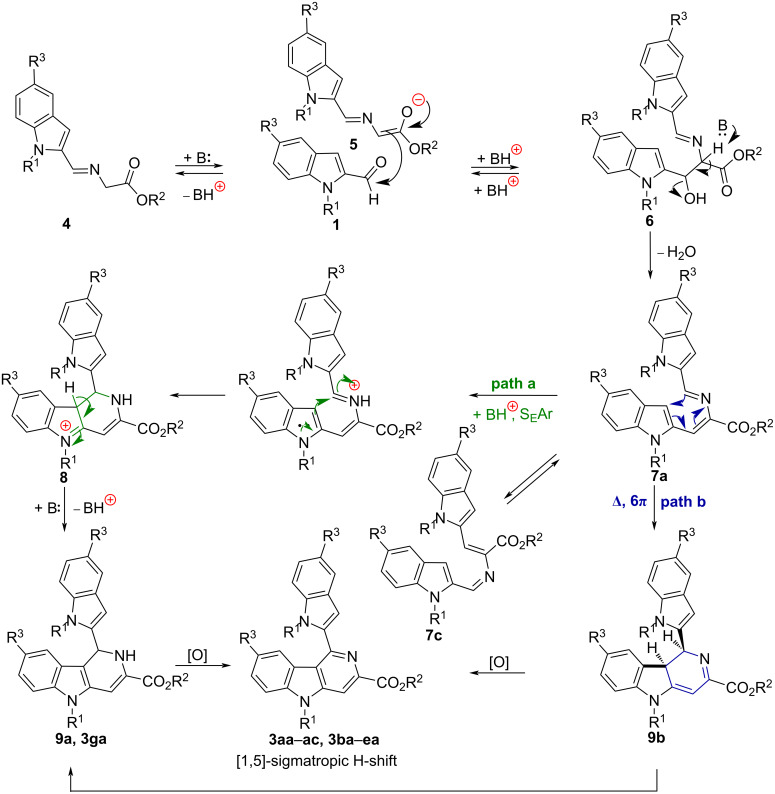
Plausible mechanism for the formation of 1,2-dihydro-γ-carboline derivative **3ga** and 1-indolyl-3,5,8-substituted γ-carbolines **3aa–ac** and **3ba–ea**.

The probable mechanistic explanation (Scheme 3) for the formation of γ-carboline derivatives **3aa–ac** and **3ba–ea** involves the initial formation of *trans-*iminoester **4** from the N*-*protected indole-2-carboxaldehydes **1a–e** and **1g**, and glycine alkyl esters **2a–c**. The Hünig’s base, DIPEA, helps abstract the active methylene proton from iminoester **4** to generate enolate ion **5**, which undergoes nucleophilic addition with another molecule of aldehyde **1** to furnish the iminoalcohol intermediate **6**. The iminoalcohol **6** undergoes dehydration under the reaction conditions to give *E*-imine/*Z*-enamine **7a** or *Z*-imine/*E*-enamine **7c** intermediates irreversibly, which plays a decisive role in determining ring closure either via path a or path b.

In path a, the protonation of the imine nitrogen in **7a** by the conjugate acid (+ BH) leads to an electrophilic aromatic substitution at the 3-position of the indole unit to form a carbon–carbon bond in the intermediate **8**. A further proton abstraction in **8** by the base then gives the 1-indolyl-3,5-substituted 1,2-dihydro-γ-carboline intermediate **9a** or **3ga**. In path b, the intermediate **7a** cyclizes via a thermal 6π-electrocyclic reaction of the conjugated triene system to form the 1-indolyl-3,5-substituted 1,9β-dihydro-γ-carboline **9b**, that may also undergo a [1,5]-sigmatropic hydrogen shift, to reinstall aromaticity of the indole ring, leading to the formation of **9a**. In situ oxidation of intermediates **9a** or **9b**, probably from the dissolved oxygen present in the reaction mixture, leads to the formation of 1-indolyl-3,5,8-substituted γ-carbolines **3aa–ac** and **3ba–ea**. We successfully isolated and characterized the 1,2-dihydro γ-carboline derivative **3ga**, which again verifies the proposed mechanism. During the formation of carbolines, the substrates, **1a–e** and **1g** were exclusively transformed to γ-carbolines or the 1,2-dihydro-γ-carboline **9a**, and no traces of any β-carboline product were observed, which proves that the heterocyclization reaction is highly regiospecific.

### Optical properties of γ-carbolines

**Figure 3:**
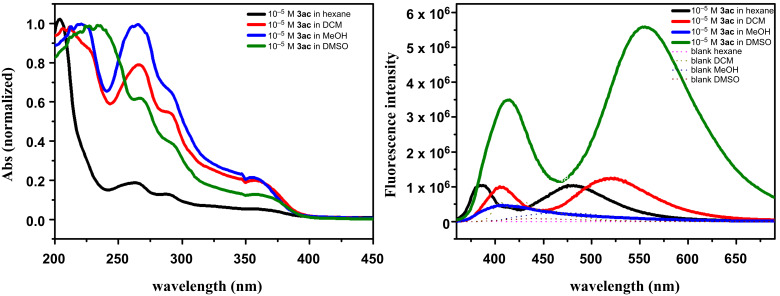
UV–vis absorption (left side) and emission (right side) spectra of **3ac** measured in different solvents.

Interestingly, the γ-carboline derivatives were found to be highly fluorescent under UV light irradiation. A systematic literature survey revealed that the structural core of carbolines had been widely exploited for the development of organic fluorescent entities, and in general, their UV absorbance ranges between 340 to 380 nm. For a deeper insight into the optical properties of the novel substituted γ-carbolines, absorption and emission studies were carried out in different organic solvents (Figure 3). The representative γ-carboline derivative *tert*-butyl-5-methyl-1-(1-methyl-1*H*-indol-2-yl)-5*H*-pyrido[4,3-*b*]indole-3-carboxylate (**3ac**) revealed similar absorption features with a shift in absorption maximum in different solvents. The highest absorption maximum (λ_max_) was observed at 230 nm for **3ac** in DMSO (Figure 3, left side).

**Figure 4:**
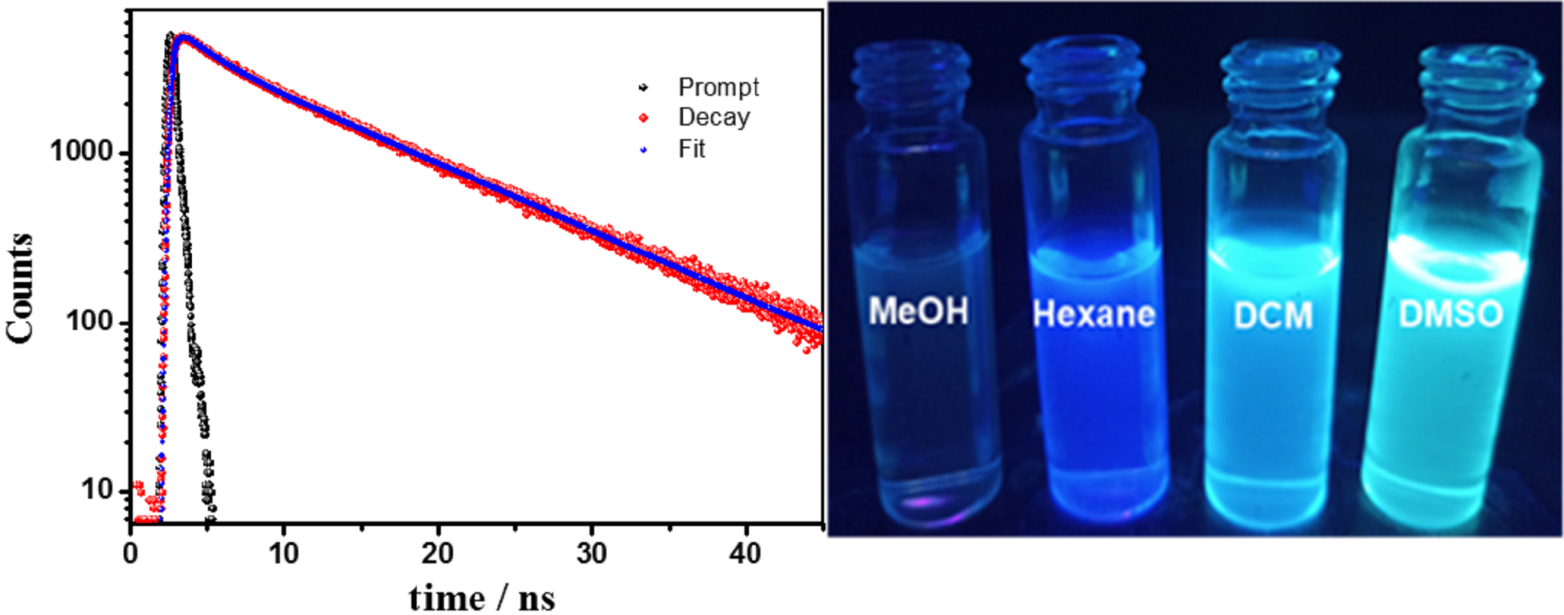
Fluorescence decay profile of **3ac** in DMSO (left side; λ_ex_ 360 nm) and 10^−5^ M solutions of compound **3ac** in four different solvents in a UV chamber (right side).

The fluorescence studies carried out for **3ac** in four different solvents revealed that the emission maxima shifted bathochromically by almost 40 nm upon changing the solvent polarity, for instance, from non-polar hexane to moderately polar dichloromethane and then highly polar DMSO (Table 2, Figure 3). The fluorescence quenching of **3ac** in methanol is attributed to the partial protonation of the carboline unit's nitrogen atoms facilitated by polar-protic solvents [33]. The fluorescence lifetimes were measured by time-correlated single-photon counting (TCSPC) experiments. The average fluorescence lifetime of compound **3ac** was found to be 8.35 ns and 4.73 ns in DMSO and DCM, respectively (Table 2, Figure 4).

**Table 2:** Optical data for γ-carboline **3ac**.

solvent	λ_abs_ (nm)	ε (10^3^ M^−1^ cm^−1^)	λ_em_ (nm)	τ (ns)

hexane	204, 262, 290, 355	0.78	386, 480	1.90
DCM	210, 266, 290, 356	1.01	405, 520	4.73
MeOH	220, 265, 290, 355	2.05	407, 422	0.99
DMSO	230, 266, 290, 357	1.67	413, 555	8.35

### Biological evaluation of γ-carbolines as anticancer agents

**Figure 5:**
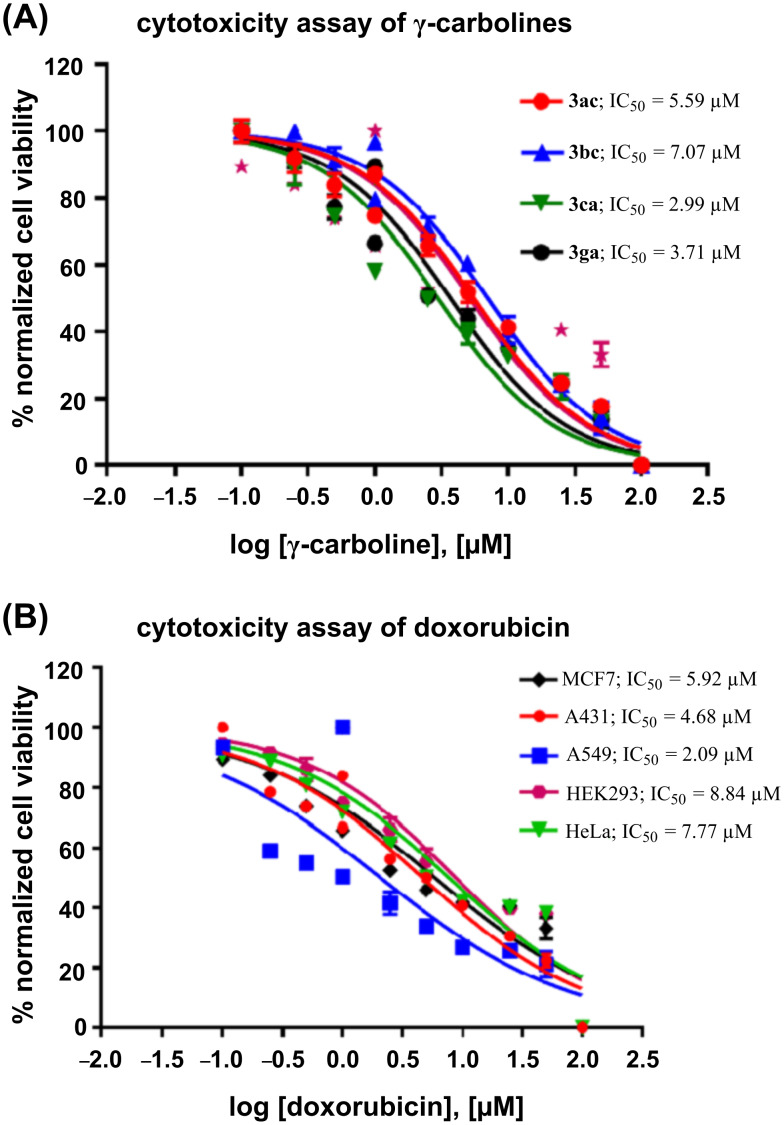
Dose–response curves for (A) γ-carbolines **3ac, 3bc, 3ca, 3ga** in the breast cancer cell line, MCF7 and (B) doxorubicin against the panel of tested cancer cell lines.

**Figure 6:**
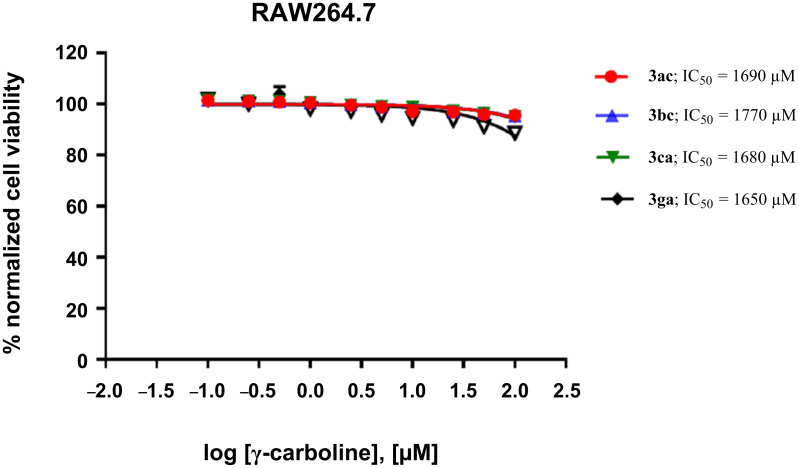
Dose–response curve of γ-carbolines **3ac, 3bc, 3ca, 3ga** in macrophage cell line, RAW264.7.

A panel of carboline derivatives **3ac**, **3bc**, **3ca**, and **3ga**, along with a standard drug, doxorubicin, were screened for their cytotoxicity against various cancer lines (Figure 5, Table 3 and Figure S2, in Supporting Information File 1) such as MCF-7 (breast cancer), HeLa (cervical cancer), HEK293 (human embryonic kidney cells), A431 (skin cancer), A549 (lung cancer), and macrophage or immune cell line (RAW 264.7). The cancer cells were treated with increasing concentrations of the carboline derivatives **3ac**, **3bc**, **3ca**, **3ga** and doxorubicin (0.1 μM, 0.25 μM, 0.5 μM, 1 μM, 2.5 μM, 5 μM, 10 μM, 25 μM, 50 μM, 100 μM) and incubated for 48 h. Half-maximal inhibitory studies show that the γ-carbolines are highly toxic to cancer cells at micromolar concentrations similar to doxorubicin, whereas they are non-cytotoxic (Figure 6) to human macrophages or immune cells.

**Table 3:** IC_50_ values of γ-carbolines **3ac**, **3bc**, **3ca**, **3ga** and doxorubicin against various cancer cell lines.

compound	IC_50_ (µM) in cancer cell lines				

γ-carboline	MCF7	A431	A549	HEK293	HeLa
**3ac**	5.59	4.89	4.76	2.29	4.89
**3bc**	7.07	9.18	5.53	7.14	8.15
**3ca**	2.99	4.47	5.27	6.73	1.30
**3ga**	3.71	3.57	5.05	4.98	1.07
doxorubicin	5.92	4.68	2.09	8.84	7.77

**Figure 7:**
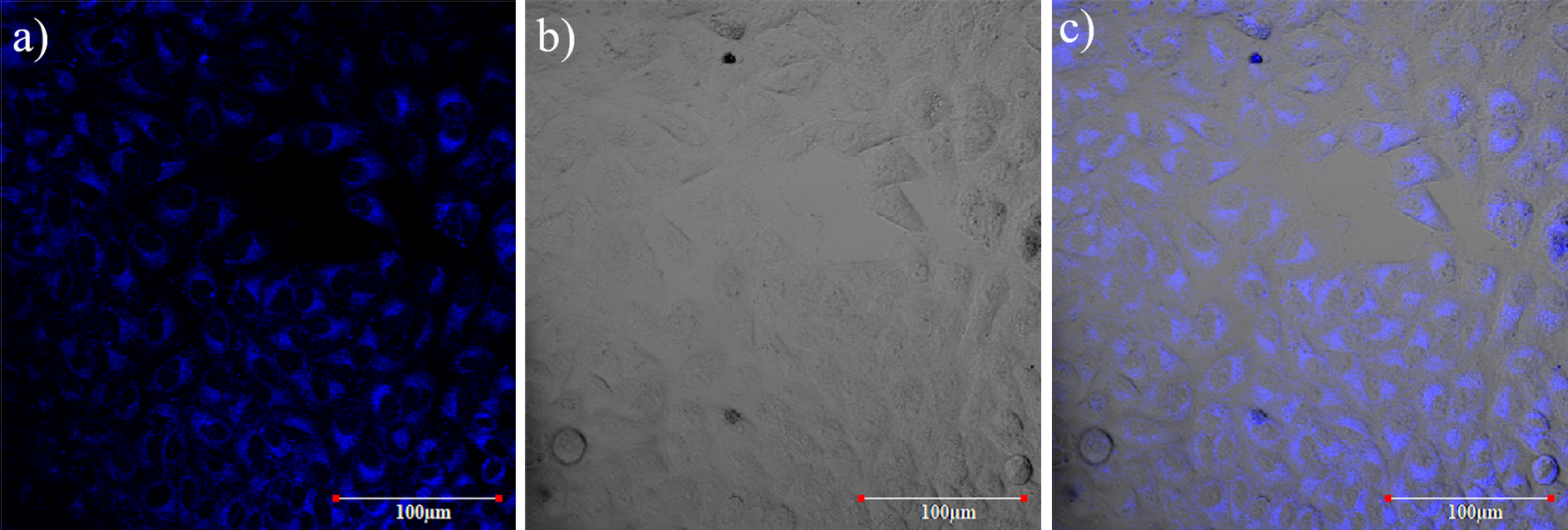
Laser scanning confocal microscopy studies (λ_ex_ = 405 nm; collection range = 420–470 nm) for uptake of carboline derivative **3ac** in HeLa cells. a) Confocal fluorescent image of HeLa cells after 3 h incubation with 10 μM concentration of **3ac** (20-fold magnification, 2-fold zoom); b) DIC image of HeLa cells; c) overlay of (a) and (b) indicating the distribution of **3ac** in the cytoplasm with distinct cell nucleus.

At last, to evaluate cell uptake of the novel γ-carboline for fluorescence imaging, live-cell imaging experiments were performed. In brief, HeLa cells were incubated with carboline **3ac** (10 μM and 100 nM), and the cellular uptake was examined using confocal microscopy (λ_ex_ = 405 nm; λ_em_ = 420–470 nm). Compound **3ac** showed excellent cytosolic uptake in cancer cells when incubated at a 10 μM concentration (Figure 7), whereas only little uptake was observed at a concentration of 100 nM (Figure S3, Supporting Information File 1).

## Conclusion

In summary, we have developed an operationally simple one-pot synthetic protocol for the synthesis of highly substituted γ-carboline derivatives. The metal- and solvent-free method provides direct access to complex molecular structures in good yield from inexpensive substrates. The optical and biological evaluations carried out for representative γ-carbolines revealed promising photophysical and anticancer properties of the core framework for developing novel theranostic applications to diagnose and treat cancer in the future.

## Experimental

### General methods and materials

All reactions were carried out in oven-dried glassware with magnetic stirring. Starting materials and other reagents were obtained from a commercial supplier and used without further purification. NMR spectra were recorded on an Avance III 400 Ascend Bruker spectrometer. CDCl_3_ and D_2_O were used as NMR solvents. Chemical shifts (δ) were reported as part per million (ppm), and TMS was used as an internal reference. High-resolution mass spectra were recorded using a Bruker Daltonik High-Performance LC-MS (electrospray ionization quadrupole time-of-flight) spectrometer. X-ray structure analysis was carried out at a Bruker KAPPA APEXII single crystal X-ray diffractometer. Melting points (mp) are uncorrected and were measured on a Veego melting point apparatus (capillary method). Analytical thin-layer chromatography (TLC) was carried out on silica gel plates (silica gel 60 F254 aluminum supported plates), and the spots were visualized with a UV lamp (254 nm and 365 nm) or using chemical staining with Brady’s reagent, KMnO_4_, ninhydrin, iodine, and bromocresol. Column chromatography was performed using silica gel (100–200 mesh or 230–400 mesh) and neutral alumina (175 mesh). DMF, DCM, DMA, toluene, and acetonitrile were dried using CaH_2_ and distilled over flame-dried 4 Å molecular sieves. THF and Et_2_O were dried over Na/benzophenone and stored over flame-dried 4 Å molecular sieves under an inert atmosphere prior to use. Organic bases, including DIPEA, Et_3_N, and DBU, were stored over anhydrous KOH pellets.

### In vitro cytotoxicity studies

#### Cytotoxicity analysis in cancer and macrophage cells

Cancer (MCF7, A431, A549, HEK293 or HeLa cell lines) or RAW264.7 cells were seeded in a 96-well plate (4,200 cells/well) and allowed to form a monolayer for a period of 48 h. Old medium was replaced with fresh medium (0.2 mL) containing an increasing concentration of γ-carboline derivatives **3ac**, **3bc**, **3ca**, **3ga** and doxorubicin (0.1 μM, 0.25 μM, 0.5 μM, 1 μM, 2.5 μM, 5 μM, 10 μM, 25 μM, 50 μM, 100 μM) and incubated for 48 h or 3 h, respectively. The medium in each well was discarded, and the cells were rinsed with PBS (3 × 0.2 mL) followed by treatment with 0.5% crystal violet (0.05 mL) for 20 minutes at room temperature. The cells were rinsed with PBS (3 × 0.2 mL) and methanol (0.20 mL) was added to each well followed by incubation for 20 minutes. The absorbance from each well proportional to the live cell was measured using a Synergy H1 multimode plate reader (BioTek Instruments, Inc., Winooski, VT, USA) at an excitation and emission wavelength of 530 nm and 590 nm, respectively.

Dose–response curves were obtained from a plot of the semi-logarithmic [conc] vs the intensity of the fluorescence emission, and the IC_50_ (concentration at which 50% of the enzymatic activity is inhibited) was calculated for the carboline derivatives or doxorubicin using GraphPad Prism, version 7.02 for Windows (GraphPad Software, San Diego, CA).

#### HeLa cell uptake study of γ-carboline **3ac**

A live-cell imaging experiment was performed with HeLa cells. The HeLa cells were placed in a 4-well confocal dish (cell count ≈ 100 cells per well) and incubated for 48 h at 37 °C under 5% CO_2_. After 3 h of incubation with carboline derivative **3ac** (10 nM, 100 nM, 1 μM, 10 μM, and 100 μM), the cellular uptake and distribution was monitored by using confocal microscopy (λ_ex_ = 405 nm; λ_em_ range = 420–470 nm).

## Supporting Information

File 1 Copies of ^1^H, ^13^C NMR spectra of **1a–h**, **3aa–ac**, **3ba–bc, 3da**, **3ea**, **3ga**, **12a–b**, **12e–f**, **12i**, **14d**, **14g** and **15**, UV calibration curves in different organic solvents for γ-carboline **3ac**, and single-crystal XRD data of **3ac**.
