# Supplementary material for: Synthesis of 1-indolyl-3,5,8-substituted γ-carbolines: one-pot solvent-free protocol and biological evaluation
Source: Beilstein J Org Chem. 2021 Jun 17;17:1453–63. doi: 10.3762/bjoc.17.101 (PMC8218543; doi:10.3762/bjoc.17.101)
Supplement: File 1 — Copies of 1H, 13C NMR spectra of 1a–h, 3aa–ac, 3ba–bc, 3da, 3ea, 3ga, 12a–b, 12e–f, 12i, 14d, 14g and 15, UV calibration curves in different organic solvents for γ-carboline 3ac, and single-crystal XRD data of 3ac. [file Beilstein_J_Org_Chem-17-1453-s001.pdf]

BEILSTEIN JOURNAL OF ORGANIC CHEMISTRY

Supporting Information

for

Synthesis of 1-indolyl-3,5,8-substituted γ-carbolines: one-pot solvent-free protocol and biological evaluation

Premansh Dudhe, Mena Asha Krishnan, Kratika Yadav, Diptendu Roy, Krishnan Venkatasubbaiah, Biswarup Pathak and Venkatesh Chelvam

Beilstein J. Org. Chem. **2021,** *17,* 1453–1463. doi:10.3762/bjoc.17.101

Copies of ^{1}H, ^{13}C NMR spectra of 1a–h, 3aa–ac, 3ba–bc, 3da, 3ea, 3ga, 12a–b, 12e–f, 12i, 14d, 14g and 15, UV calibration curves in different organic solvents for γ-carboline 3ac, and single-crystal XRD data of 3ac

License and Terms: This is a supporting information file under the terms of the Creative Commons Attribution License (https://creativecommons.org/licenses/by/4.0). Please note that the reuse, redistribution and reproduction in particular requires that the author(s) and source are credited and that individual graphics may be subject to special legal provisions.
The license is subject to the *Beilstein Journal of Organic Chemistry* terms and conditions: (https://www.beilstein-journals.org/bjoc/terms)

Table of contents

1. General procedure for the synthesis of 1-indolyl-3,5,8-substituted γ-carboline **3aa–ac**, **3ba-ea**, and 1-indolyl-1,2-dihydro-3,5-substituted γ-carboline **3ga** derivatives S2
2. Synthesis of precursors or indole substrates S9
3. UV calibration of γ-carboline **3ac** in organic solvents S20
4. Single-crystal XRD analysis of **3ac** S21
5. Dose–response or IC_{50} curves for γ-carbolines, **3ac**, **3bc**, **3ca**, and **3ga** S23
6. Confocal microscopy studies in HeLa cells with 100 nM of **3ac** S25
7. Copies of ^{1}H and ^{13}C NMR spectra of **1a–h**, **3aa–ac**, **3ba–bc, 3da**, **3ea**, **3ga**, **12a–b**, **12e–f**, **12i**, **14d**, **14g** and **15**. S26
8. References S78

1. General procedure for the synthesis of 1-indolyl-3,5,8-substituted γ-carboline (**3aa–ac**, **3ba–ea**) and 1-indolyl-1,2-dihydro-3,5-substituted γ-carboline (**3ga**) derivatives

A mixture of the N-substituted indole-2-carbaldehyde (**1a–h**, 2.0 mmol), glycine alkyl ester hydrochloride (**2a–c**, 1.0 mmol), and DIPEA (3.5 mmol) was heated in a sealed tube for 3–8 h at 120 °C. After the completion of the reaction, as evident by TLC, the reaction mixture was diluted with dichloromethane (CH_2Cl_2, 10 mL) and washed with cold brine (10 mL). The aqueous layer was again extracted with CH_2Cl_2 (3 × 10 mL). Organic layers were pooled and dried over anhydrous Na_2SO_4, filtered, concentrated under reduced pressure, and purified over neutral alumina gel (175 mesh) column chromatography, using a mixture of ethyl acetate and hexane as eluent to afford the products **3aa–ac**, **3ba–ea** and **3ga**.

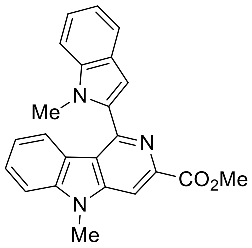

3aa

Methyl 5-methyl-1-(1-methyl-1H-indol-2-yl)-5H-pyrido[4,3-b]indole-3-carboxylate (**3aa**). According to the general procedure mentioned above, **1a** (0.100 g, 0.62 mmol), **2a** (39 mg, 0.31 mmol), and DIPEA (190 µL, 1.09 mmol) were heated in a sealed tube at 120 °C for 6 h. After workup, the crude reaction mixture was purified through alumina (neutral, 175 mesh) column chromatography using hexane/EtOAc 80:20 as eluent; Yield 70% (80 mg); Yellow solid; m.p. = 210–212 °C; R_f 0.35 (2:1 hexane-EtOAc); IR (KBr) 3055 (=C–H), 2956–2854 (C–H), 1734 (C=O), 1687–1534 (C=C), 1407–1376 (C–H bend), 782 (=C–H bend) cm^{-1}; ^1H NMR (400 MHz, $CDCl_3$) δ 8.31 (s, 1H), 7.82 (d, J = 8.0 Hz, 1H), 7.74 (d, J = 8.0 Hz, 1H), 7.56 (dd, J = 8.0, 7.3 Hz, 1H), 7.51 (d, J = 8.3 Hz, 1H), 7.45 (d, J = 8.3 Hz, 1H), 7.33 (dd, J = 8.0, 7.8 Hz, 1H), 7.19 (dd, J = 7.5, 7.5 Hz, 1H), 7.15 (dd, J = 7.6, 7.5 Hz, 1H), 6.99 (s, 1H), 4.05 (s, 3H), 4.00 (s, 3H), 3.75 (s, 3H); ^{13}C NMR (100 MHz, $CDCl_3$) δ 166.9, 146.3, 145.8, 142.9, 142.2, 138.3, 137.6, 128.1, 127.9, 123.1, 122.4, 121.3, 121.2, 121.1, 120.8, 119.8, 109.8, 109.1, 105.7, 104.3, 53.0, 31.0, 29.6; HRMS (ESI) calcd for $[C_{23}H_{19}N_3O_2+H^+]$ 370.1550, found 370.1515.

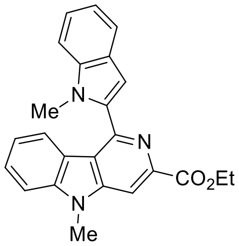

3ab *Ethyl 5-methyl-1-(1-methyl-1H-indol-2-yl)-5H-pyrido[4,3-b]indole-3-carboxylate* (**3ab**). According to the general procedure mentioned above, **1a** (0.100 g, 0.62 mmol), **2b** (43 mg, 0.31 mmol), and DIPEA (190 µL, 1.09 mmol) were heated in a sealed tube at 120 °C for 6 h. After workup, the crude reaction mixture was purified through alumina (neutral, 175 mesh) column chromatography using hexane/EtOAc 85:15 as eluent; Yield 66% (78 mg); Yellow solid; m.p. = 175–177 °C; R_f 0.40 (2:1 hexane-EtOAc); IR (KBr) 3058 (=C–H), 2988–2851 (C–H), 1735 (C=O), 1704–1536 (C=C), 1409–1375 (C–H bend), 780 (=C–H bend) cm^{-1}; ^1H NMR (400 MHz, $CDCl_3$) δ 8.27 (s, 1H), 7.89 (d, J = 8.0 Hz, 1H), 7.73 (d, J = 7.8 Hz, 1H), 7.56 (dd, J = 7.8, 7.3 Hz, 1H), 7.50 (d, J = 8.0 Hz, 1H), 7.46 (d, J = 8.0 Hz, 1H), 7.33 (dd, J = 7.8, 7.8 Hz, 1H), 7.19 (m, 2H), 7.01 (s, 1H), 4.53 (q, J = 7.0 Hz, 2H), 3.99 (s, 3H), 3.79 (s, 3H), 1.48 (t, J = 7.0 Hz, 3H); ^{13}C NMR (100 MHz, $CDCl_3$) δ 166.3, 146.3, 145.9, 143.2, 142.2, 138.3, 137.6, 128.0, 127.8, 123.1, 122.4, 121.2, 121.1, 120.9, 120.8, 119.8, 109.8, 109.1, 105.5, 104.4, 61.9, 31.1, 29.5, 14.5; HRMS (ESI) calcd for $[C_{24}H_{21}N_3O_2+H^+]$ 384.1707, found 384.1672.

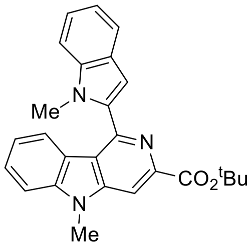

3ac *tert-Butyl 5-methyl-1-(1-methyl-1H-indol-2-yl)-5H-pyrido[4,3-b]indole-3-carboxylate* (**3ac**). According to the general procedure mentioned above, **1a** (0.100 g, 0.62 mmol), **2c** (52 mg, 0.31 mmol), and DIPEA (190 µL, 1.09 mmol) were heated in a sealed tube at 120 °C for 8 h. After workup, the crude reaction mixture was purified through alumina (neutral, 175 mesh) column chromatography using hexane/EtOAc 90:10 as eluent; Yield 67% (85 mg); Yellow solid; m.p. = 200–202 °C; R_f 0.60 (2:1 hexane-EtOAc); IR (KBr) 3053 (=C–H), 2972–2852 (C–H), 1729 (C=O), 1686–1532 (C=C), 1412–1365 (C–H bend), 781 (=C–H bend) cm^{-1}; ^1H NMR (400 MHz, $CDCl_3$) δ 8.14 (s, 1H), 8.07 (d, J = 8.0 Hz, 1H), 7.73 (d, J = 7.8 Hz, 1H), 7.55 (dd, J = 7.5, 7.5 Hz, 1H), 7.52–7.43 (m, 2H), 7.33 (dd, J = 8.0, 7.3 Hz, 1H), 7.23–7.11 (m, 2H),7.06 (s, 1H), 3.97 (s, 3H), 3.88 (s, 3H), 1.69 (s, 9H);^{13}C NMR (100 MHz, $CDCl_3$) δ 165.2, 146.2, 146.1, 144.3, 142.2, 138.4, 137.7, 127.8, 127.7,

123.1, 122.4, 121.2, 120.9*, 120.3, 119.7, 109.8, 109.0, 104.7, 104.6, 81.9, 31.2, 29.5, 28.3; HRMS (ESI) calcd for [$C_{26}H_{25}N_3O_2$+H^+] 412.2020, found 412.2012.

*Higher intensity carbon

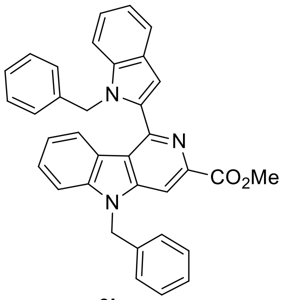

3ba *Methyl 5-benzyl-1-(1-benzyl-1H-indol-2-yl)-5H-pyrido[4,3-b]indole-3-carboxylate* (**3ba**). According to the general procedure mentioned above, **1b** (100 mg, 0.42 mmol), **2a** (26 mg, 0.21 mmol), and DIPEA (95 µL, 0.74 mmol) were heated in a sealed tube at 120 °C for 6 h. After workup, the crude reaction mixture was purified through alumina (neutral, 175 mesh) column chromatography using hexane/EtOAc 85:15 as eluent; Yield 58% (63 mg); Yellow solid; m.p. = 168–170 °C; R_f 0.60 (2:1 hexane-EtOAc);IR (ATR) 3062 (=C–H), 2920–2850 (C–H), 1710 (C=O), 1667–1528 (C=C), 1467–1315 (C–H bend), 787–694 (=C–H bend) cm^{-1};^{1}H NMR (400 MHz, $CDCl_3$) δ 8.20 (s, 1H), 8.13 (d, *J* = 8.0 Hz, 1H), 7.75 (d, *J* = 7.5 Hz, 1H), 7.48 (dd, *J* = 7.5, 7.3 Hz, 1H), 7.41 (m, 2H), 7.35–7.23 (m, 4H), 7.20 (d, *J* = 7.5 Hz, 1H), 7.16 (d, *J* = 8.0 Hz, 1H), 7.14–7.08 (m, 3H), 6.99–6.89 (m, 5H), 5.67 (s, 2H), 5.60 (s, 2H), 3.98 (s, 3H); ^{13}C NMR (100 MHz, $CDCl_3$) δ 166.8, 146.5, 145.8, 142.9, 141.7, 138.3, 138.1, 136.9, 135.6, 129.0, 128.1*, 128.0, 127.9, 126.7, 126.6, 126.3, 123.3, 122.7, 121.3, 121.13, 121.06, 121.0, 120.0, 110.6, 109.6, 105.7, 105.5, 52.9, 47.7, 46.8; HRMS (ESI) calcd for [$C_{35}H_{27}N_3O_2$+H^+] 522.2176, found 522.2160.

*Higher intensity carbon

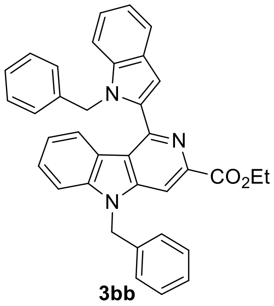

3bb *Ethyl 5-benzyl-1-(1-benzyl-1H-indol-2-yl)-5H-pyrido[4,3-b]indole-3-carboxylate* (**3bb**). According to the general procedure mentioned above, **1b** (0.100 g, 0.62 mmol), **2b** (43 mg, 0.31 mmol), and DIPEA (190 µL, 1.09 mmol) were heated in a sealed tube at 120 °C for 6 h. After workup, the crude reaction mixture was purified through alumina (neutral, 175 mesh) column chromatography using hexane/EtOAc 80:20 as eluent;

Yield 51% (85 mg); Reddish yellow liquid; R_f 0.40 (2:1 hexane-EtOAc); IR (KBr) 3059 (=C–H), 2965–2860 (C–H), 1722 (C=O), 1609–1574 (C=C), 1423–1383 (C–H bend), 799 (=C–H bend) cm^{-1}; ^1H NMR (400 MHz, $CDCl_3$) δ8.21–8.14 (m, 2H), 7.75 (d, J = 7.5 Hz, 1H),7.47 (dd, J = 7.8, 7.3 Hz, 1H),7.40 (m, 2H), 7.35–7.23 (m, 4H),7.20 (d, J = 7.3 Hz, 1H), 7.16 (d, J = 7.3 Hz, 1H),7.14–7.08 (m, 3H), 7.02–6.89 (m, 5H),5.72 (s, 2H), 5.59 (s, 2H), 4.45 (q, J = 7.1 Hz, 2H), 1.40 (t, J = 7.1 Hz, 3H); ^{13}C NMR (100 MHz, $CDCl_3$) δ 166.2, 146.5, 145.9, 143.2, 141.7, 138.4, 138.1, 136.9, 135.7, 129.0, 128.2, 128.1, 128.0, 127.8, 126.7, 126.6, 126.4, 123.3, 122.7, 121.2, 121.1, 121.0, 120.9, 120.0, 110.6, 109.6, 105.52, 105.48, 61.8, 47.7, 46.8, 14.4; HRMS (ESI) calcd for[$C_{36}H_{29}N_3O_2+H^+$] 536.2333, found 536.2349.

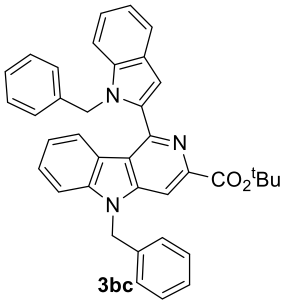

3bc

tert-Butyl 5-benzyl-1-(1-benzyl-1H-indol-2-yl)-5H-pyrido[4,3-b]indole-3-carboxylate (**3bc**). According to the general procedure mentioned above, **1b** (0.100 g, 0.62 mmol), **2c** (52 mg, 0.31 mmol), and DIPEA (190 µL, 1.09 mmol) were heated in a sealed tube at 120 °C for 8 h. After workup, the crude reaction mixture was purified through alumina (neutral, 175 mesh) column chromatography using hexane/EtOAc 85:15 as eluent; Yield 47% (82 mg); Yellow solid; m.p. = 148–150 °C; R_f 0.60 (2:1 hexane-EtOAc); IR (ATR) 3062 (=C–H), 2926–2848 (C–H), 1706 (C=O), 1665–1531 (C=C), 1495–1323 (C–H bend), 782–694 (=C–H bend) cm^{-1}; ^1H NMR (400 MHz, $CDCl_3$) δ 8.28 (d, J = 8.0 Hz, 1H), 8.06 (s, 1H), 7.75 (d, J = 7.8 Hz, 1H), 7.47 (dd, J = 7.5, 7.3 Hz, 1H),7.43–7.36 (m, 2H),7.34–7.22 (m, 4H), 7.21–7.15 (m, 2H), 7.15–7.10 (m, 3H), 7.02–6.93 (m, 5H),5.86 (s, 2H), 5.58 (s, 2H), 1.63 (s, 9H); ^{13}C NMR (100 MHz, $CDCl_3$) δ 165.2, 146.4, 146.1, 144.5, 141.7, 138.6, 138.1, 137.0, 135.8, 129.0, 128.2, 128.0, 127.84, 127.75, 126.7, 126.5, 126.4, 123.3, 122.7, 121.2, 121.1, 120.9, 120.4, 120.0, 110.6, 109.6, 105.7, 104.9, 81.8, 47.5, 46.8, 28.2; HRMS (ESI) calcd for[$C_{38}H_{33}N_3O_2+H^+$] 564.2646, found 564.2644.

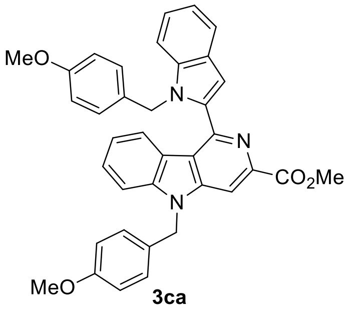

Methyl 5-(4-methoxybenzyl)-1-(1-(4-methoxybenzyl)-1H-indol-2-yl)-5H-pyrido[4,3-b]indole-3-carboxylate (**3ca**). According to the general procedure mentioned above, **1c** (100 mg, 0.38 mmol), **2a** (24 mg, 0.19 mmol) and DIPEA (120 µL, 0.66 mmol) were heated in a sealed tube at 120 °C for 3 h. After workup, the crude reaction mixture was purified through alumina (neutral, 175 mesh) column chromatography using hexane/EtOAc 85:15 as eluent; Yield 60% (66 mg); Yellow solid;m.p. = 106–108 °C;R_f 0.60 (2:1 hexane-EtOAc); IR(ATR) 3056 (=C–H), 2952–2835 (C–H), 1737 (C=O), 1664–1512 (C=C), 1457–1348 (C–H bend), 1106–989 (C–O), 819–695 (=C–H bend) cm^{-1};^{1}H NMR (400 MHz, $CDCl_3$) δ 8.22 (s, 1H), 8.04 (d, J = 8.0 Hz, 1H), 7.73 (d, J = 7.8 Hz, 1H), 7.51–7.39 (m, 3H), 7.27 (d, J = 7.3 Hz, 1H), 7.18 (dd, J = 7.5, 7.0 Hz, 1H), 7.12 (dd, J = 7.3, 7.3 Hz, 1H), 7.07 (d, J= 8.5 Hz, 2H), 7.06 (s, 1H), 6.85 (d, J = 8.8 Hz, 2H), 6.83 (d, J = 8.8 Hz, 2H), 6.45 (d, J = 8.5 Hz, 2H), 5.55 (s, 2H), 5.54 (s, 2H), 3.99 (s, 3H), 3.76 (s, 3H), 3.52 (s, 3H); ^{13}C NMR (100 MHz, $CDCl_3$) δ 166.9, 159.4, 158.4, 146.6, 145.7, 142.9, 141.7, 138.0, 137.0, 130.4, 128.1, 127.95, 127.92, 127.75, 127.68, 123.3, 122.6, 121.2, 121.1, 121.04, 121.01, 119.9, 114.5, 113.5, 110.6, 109.6, 105.7, 105.3, 55.3, 55.1, 52.9, 47.2, 46.4; HRMS (ESI) calcd for[$C_{37}H_{31}N_3O_4$+H^+] 582.2387, found 582.2373.

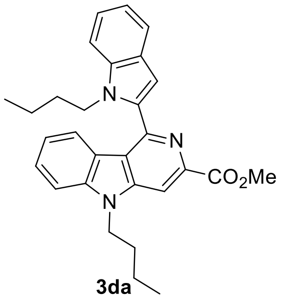

Methyl 5-butyl-1-(1-butyl-1H-indol-2-yl)-5H-pyrido[4,3-b]indole-3-carboxylate(**3da**). According to the general procedure mentioned above, **1d** (0.100 g, 0.49 mmol), **2a** (31 mg, 0.25 mmol), and DIPEA (114 µL, 0.88 mmol) were heated in a sealed tube at 120 °C for 3 h. After workup, the crude reaction mixture was purified through alumina (neutral, 175 mesh) column chromatography using hexane/EtOAc 85:15 as eluent;

Yield 54% (61 mg); Yellow liquid; R_f 0.60 (2:1 hexane-EtOAc); IR (KBr) 3064 (=C–H), 2972–2854 (C–H), 1726 (C=O), 1621–1570 (C=C), 1462–1317 (C–H bend), 796 (=C–H bend) cm^{-1}; ^{1}H NMR (400 MHz, $CDCl_3$) δ 8.27 (s, 1H), 8.00 (d, J = 8.0 Hz, 1H), 7.72 (d, J = 7.5 Hz, 1H), 7.59–7.45 (m, 3H), 7.30 (dd, J = 7.6, 7.5 Hz, 1H), 7.18 (dd, J = 8.2, 7.2 Hz, 1H), 7.13 (dd, J = 8.2, 7.3 Hz, 1H), 6.97 (s, 1H), 4.43 (t, J = 6.8 Hz, 2H), 4.34 (t, J = 7.0 Hz, 2H), 4.05 (s, 3H), 2.00–1.87 (m, 2H), 1.71–1.60 (m, 2H), 1.52–1.39 (m, 2H), 1.16–1.04 (m, 2H), 0.99 (t, J = 7.0 Hz, 3H), 0.62 (t, J = 7.3 Hz, 3H); ^{13}C NMR (100 MHz, $CDCl_3$) δ 167.1, 146.8, 145.4, 142.7, 141.6, 137.7, 136.9, 127.92, 127.87, 123.3, 122.2, 121.3, 120.9, 120.8*, 119.6, 110.2, 109.3, 105.6, 104.7, 52.9, 43.9, 43.4, 32.2, 31.1, 20.6, 20.0, 13.9, 13.5; HRMS (ESI) calcd for[$C_{29}H_{31}N_3O_2+H^+$] 454.2489, found 454.2559.

*Carbon merged

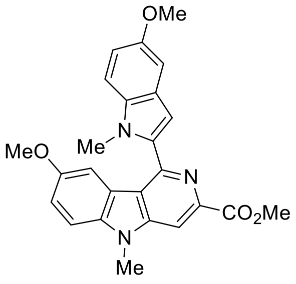

3ea

Methyl 8-methoxy-1-(5-methoxy-1-methyl-1H-indol-2-yl)-5-methyl-5H-pyrido[4,3-b]indole-3-carboxylate (**3ea**). According to the general procedure mentioned above, **1e** (70 mg, 0.37 mmol), **2a** (23 mg, 0.18 mmol), and DIPEA (110 µL, 0.63 mmol) were heated in a sealed tube at 120 °C for 3 h. After workup, the crude reaction mixture was purified through alumina (neutral, 175 mesh) column chromatography using hexane/EtOAc 85:15 as eluent; Yield 72% (55 mg); Yellow solid; m.p. = 160–162 °C; R_f 0.60 (2:1 hexane-EtOAc); IR (ATR) 3070 (=C–H), 2957–2850 (C–H), 1701 (C=O), 1660–1528 (C=C), 1485–1329 (C–H bend), 1105–991 (C–O), 810–688 (=C–H bend) cm^{-1}; ^{1}H NMR (400 MHz, $CDCl_3$) δ 8.26 (s, 1H), 7.40 (d,J = 9.8 Hz, 1H), 7.32 (d, J = 8.8 Hz, 1H), 7.21–7.16 (m, 2H), 7.15 (d, J = 2.0 Hz, 1H), 6.97 (dd, J = 9.0 Hz, 2.3 Hz, 1H), 6.91 (s, 1H), 4.05 (s, 3H), 3.96 (s, 3H), 3.89 (s, 3H), 3.71 (s, 3H), 3.55 (s, 3H); ^{13}C NMR (100 MHz, $CDCl_3$) δ 167.0, 154.8, 154.3, 146.3, 145.8, 142.6, 137.9, 137.2, 133.8, 128.1, 121.2, 120.8, 117.7, 112.8, 110.4, 109.9, 105.8, 105.0, 103.8, 102.5, 55.8, 55.7, 53.0, 31.1, 29.6; HRMS (ESI) calcd for[$C_{25}H_{23}N_3O_4+H^+$] 430.1761, found 430.1764.

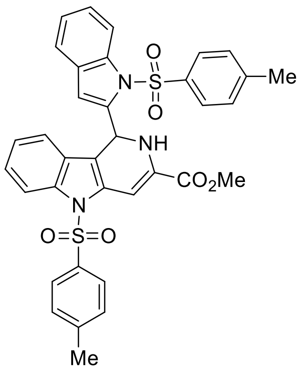

3ga

Methyl 5-tosyl-1-(1-tosyl-1H-indol-2-yl)-2,5-dihydro-1H-pyrido[4,3-b]indole-3-carboxylate (**3ga**). According to the general procedure mentioned above, **1g** (0.100 g, 0.33 mmol), **2a** (21 mg, 0.17 mmol), and DIPEA (101 µL, 0.58 mmol) were heated in a sealed tube at 120 °C for 8 h. After workup, the crude reaction mixture was purified through alumina (neutral, 175 mesh) column chromatography using hexane/EtOAc 90:10 as eluent; Yield 63% (70 mg); Yellow solid; m.p. = 205–207 °C; R_f 0.60 (2:1 hexane-EtOAc); IR (ATR) 3413 (N–H), 3062 (=C–H), 2956–2850 (C–H), 1706 (C=O), 1633–1489 (C=C), 1448–1350 (C–H bend), 1307 (N–S=O), 1145 (S=O),812–687 (=C–H bend) cm^{-1}; ^{1}H NMR (400 MHz, $CDCl_3$) δ 8.16 (dd, J = 8.0, 8.0 Hz, 2H), 7.73 (d, J = 8.5 Hz, 2H), 7.70 (d, J = 8.5 Hz, 2H), 7.32–7.26 (m, 3H),7.24–7.19 (m, 3H), 7.18–7.14 (m, 2H), 7.09 (d, J = 2.0 Hz, 1H), 6.98 (dd, J = 7.5, 7.3 Hz, 1H), 6.59 (d, J = 2.0 Hz, 1H), 6.38 (d, J = 7.8 Hz, 1H), 5.90 (s, 1H), 5.64 (s, 1H), 3.83 (s, 3H), 2.41 (s, 3H), 2.37 (s, 3H); ^{13}C NMR (100 MHz, $CDCl_3$) δ 163.9, 145.4, 145.0, 139.4, 137.7, 137.4, 136.3, 135.2, 135.0, 132.6, 130.1, 129.9, 128.8, 127.7, 126.8, 126.3, 125.2, 124.4, 124.0, 123.8, 121.2, 117.5, 114.9,114.8, 113.1, 110.6, 94.4, 52.6, 47.9, 21.7, 21.6; HRMS* (ESI) calcdfor[$C_{35}H_{27}N_3O_6S_2+H^+$] 650.1414, found 650.1386.

*HRMS peak corresponds to dehydrogenated (aromatized) form of **3ga**.

2. Synthesis of precursors or indole substrates

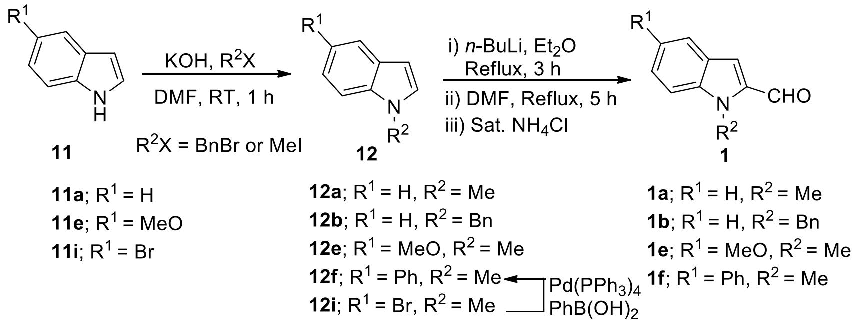

Scheme S1. Synthesis of N-substituted indole-2-carboxaldehyde derivatives **1a**, **1b**, **1e**, and **1f**.

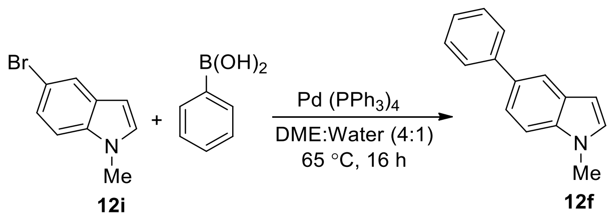

Scheme S2. Preparation of **12f** by Suzuki (sp^2 C–sp^2 C) reaction.

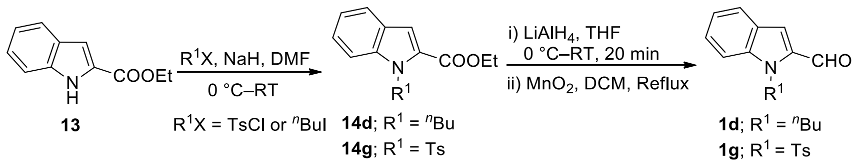

Scheme S3. Synthesis of N-substituted indole-2-carboxaldehyde derivatives **1d** and **1g**.

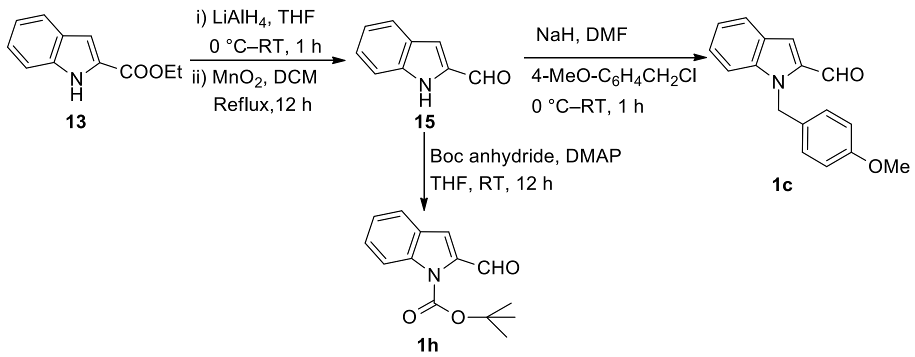

Scheme S4. Synthesis of N-substituted indole-2-carboxaldehydes **1c** and **1h**.

Synthesis of precursors or indole substrates 1a–h

A. Synthesis of N-substituted indole-2-carboxaldehyde derivatives 1a, 1b, 1e, and 1f

(a) General procedure for the synthesis of N-substituted indoles **12a**, **12b**, **12e**, and **12i**

Crushed potassium hydroxide (5.0 equiv) was dissolved in *N,N*-dimethyl formamide (5 mL) in a two-necked round-bottomed flask (50 mL) by stirring for 5 min at room temperature. Then, 5-substituted indole (**11a**, **11e**, or **11i**, 1.0 equiv) was added in one portion and the mixture was stirred for another 5 min under an inert atmosphere. Subsequently, the reaction mixture was cooled to 0 °C, and benzyl bromide or methyl iodide (2.0 equiv) was added drop-wise using a glass syringe over a period of 5 min. The reaction mixture was warmed to room temperature, monitored by TLC, and stirred for another 1 h. After the completion of the reaction, the reaction mixture was quenched with cold brine (10 mL) and extracted with EtOAc (3 × 10 mL). The combined organic layer was dried over anhydrous Na_2SO_4, filtered, and concentrated under reduced pressure. The crude residue was purified by silica gel (230–400 mesh) column chromatography using hexane/EtOAc solvent mixtures as eluent to afford **12a, b**, **12e** and **12i**.

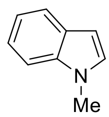

12a *1-Methyl-1H-indole*[6] (**12a**). According to the general procedure mentioned above, methyl iodide (1.06 mL, 17.08 mmol) was added to a mixture of KOH (2.40 g, 42.68 mmol) and indole (**11a**, 1.0 g, 8.54 mmol) in DMF (5 mL) drop-wise and the reaction mixture was stirred at room temperature for 1 h. After workup, the crude residue was purified over silica gel (230–400 mesh) column chromatography using hexane/EtOAc 99.0:1.0 as eluent. Yield 92% (1.03 g); Off white liquid; R_f 0.50 (9:1 hexane-EtOAc); ^{1}H NMR (400 MHz, $CDCl_3$) δ 7.62 (d, J = 7.8 Hz, 1H), 7.30 (d, J = 7.5 Hz, 1H), 7.21 (dd, J = 7.8, 7.3 Hz, 1H), 7.10 (dd, J = 7.5, 7.3 Hz, 1H), 7.01 (d, J = 3.0 Hz, 1H), 6.47 (d, J = 3.0 Hz, 1H), 3.74 (s, 1H); ^{13}C NMR (100 MHz, $CDCl_3$) δ 136.8, 128.8, 128.5, 121.5, 120.9, 119.3, 109.2, 100.9, 32.8.

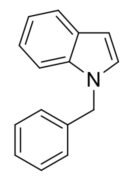

12b *1-Benzyl-1H-indole*[7] (**12b**). According to the general procedure mentioned above, benzyl bromide (68 µL, 5.12 mmol) was added to a mixture of KOH (0.957 g, 17.07 mmol) and indole **11a** (0.500 g, 4.27 mmol) in DMF (3 mL) drop-wise and the reaction mixture was stirred at room temperature for 2 h. After workup, the crude residue was purified over silica gel (230–400 mesh) column chromatography using hexane as eluent. Yield 93% (0.824 g); Pale green liquid; R_f 0.60 (9:1 hexane-EtOAc); ^{1}H NMR (400 MHz, $CDCl_3$) δ 7.65 (d, J = 7.8 Hz, 1H), 7.32–7.20 (m, 4H), 7.16 (ddd, J = 7.6, 7.0, 0.7 Hz), 7.13–7.06 (m, 4H), 6.55 (d, J =

3.0 Hz, 1H), 5.31 (s, 2H); ^{13}C NMR (100 MHz, $CDCl_3$) δ 137.6, 136.3, 128.8, 128.7, 128.3, 127.6, 126.8, 121.7, 121.0, 119.6, 109.7, 101.7, 50.1.

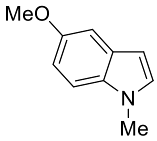

12e *5-Methoxy-1-methyl-1H-indole*[8] (**12e**). According to the general procedure mentioned above, methyl iodide (169 µL, 2.72 mmol) was added to a mixture of KOH (0.380 g, 6.79 mmol) and 5-methoxyindole (**11e**, 0.20 g, 1.36 mmol) in DMF (3 mL) drop-wise and the reaction mixture was stirred at room temperature for 30 min. After workup, the crude residue was purified over silica gel (230–400 mesh) column chromatography using hexane/EtOAc 98:2 as eluent. Yield 99% (0.216 g); White solid; R_f 0.40 (9:1 hexane-EtOAc); ^{1}H NMR (400 MHz, $CDCl_3$) δ 7.22 (d, J = 8.8 Hz, 1H), 7.13–7.09 (m, 1H), 7.02 (d, J = 2.5 Hz, 1H), 6.94–6.87 (m, 1H), 6.42 (d, J = 2.5 Hz, 1H), 3.86 (s, 3H), 3.76 (s, 3H); ^{13}C NMR (100 MHz, $CDCl_3$) δ 154.0, 132.2, 129.3, 128.8, 111.9, 109.9, 102.6, 100.4, 55.9, 33.0.

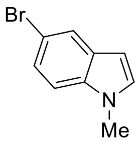

12i *5-Bromo-1-methyl-1H-indole*[8] (**12i**). According to the general procedure mentioned above, methyl iodide (317 µL, 5.10 mmol) was added to a mixture of KOH (0.71 g, 12.75 mmol) and 5-bromoindole (**11i**, 0.50 g, 2.55 mmol) in DMF (3 mL) drop-wise and the reaction mixture was stirred at room temperature for 1 h. After workup, the crude residue was purified over silica gel (230–400 mesh) column chromatography using hexane/EtOAc (98:2) as eluent. Yield 99% (0.525 g); Pale yellow liquid; R_f 0.45 (9:1 hexane-EtOAc); ^{1}H NMR (400 MHz, $CDCl_3$) δ 7.75 (d, J = 1.5 Hz, 1H), 7.30 (dd, J = 8.8, 1.5 Hz, 1H), 7.18 (d, J = 8.8 Hz, 1H), 7.04 (d, J = 3.0 Hz, 1H), 6.43 (d, J = 3.0 Hz, 1H), 3.76 (s, 3H); ^{13}C NMR (100 MHz, $CDCl_3$) δ 135.4, 130.2, 130.0, 124.3, 123.3, 112.7, 110.7, 100.6, 33.0.

(b) Preparation of **12f** by Suzuki (sp^2 C–sp^2 C) reaction

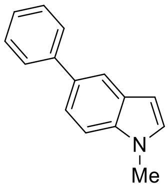

12f *1-Methyl-5-phenyl-1H-indole*[9] (**12f**). 5-Bromo-1-methyl-1*H*-indole (**12i**, 0.200 g, 0.95 mmol) and phenylboronic acid (0.14 g, 1.14 mmol) were dissolved in a mixture (4:1) of dimethoxyethane (DME) and milli-Q water in a two-necked round-bottomed flask (100 mL) and the solution was degassed (by bubbling N_2 gas for 30 min). Then, K_3PO_4 (403 mg,

1.90 mmol) and $Pd(PPh_3)_4$ (23 mg, 0.02 mmol) were added to the mixture at room temperature in one portion. The reaction mixture was heated at 65 °C using an oil bath under inert atmosphere and monitored by TLC for 16 h. After the completion of the reaction, the reaction was quenched with brine (5 mL) and extracted with EtOAc (3 × 10 mL). The combined organic layers were dried over anhydrous Na_2SO_4, filtered, and concentrated under reduced pressure. The crude residue was purified by silica gel (230–400 mesh) column chromatography using hexane/EtOAc 96:4 as eluent to afford **12f**. Yield 81% (0.160 g); White solid; R_f 0.55 (9:1 hexane-EtOAc); ^{1}H NMR (400 MHz, $CDCl_3$) δ 7.87 (d, J = 1.2 Hz, 1H), 7.68 (d, J = 7.8 Hz, 2H), 7.51 (dd, J = 8.3, 1.2 Hz, 1H), 7.46 (dd, J = 7.8, 7.6 Hz, 2H), 7.40 (d, J = 8.3 Hz, 1H), 7.33 (dd, J = 7.3, 7.3 Hz, 1H), 7.09 (d, J = 2.8 Hz, 1H), 6.56 (d, J = 2.8 Hz, 1H), 3.82 (s, 3H); ^{13}C NMR (100 MHz, $CDCl_3$) δ 142.7, 136.3, 132.9, 129.5, 129.0, 128.7, 127.5, 126.3, 121.5, 119.5, 109.5, 101.4, 33.0.

(c) General procedure for the synthesis of N-substituted indole-2-carboxaldehydes **1a**, **1b, 1e, 1f**)

The N-substituted indole (**12a**, **12b**, **12e** or **12f**, 1.0 equiv.) was dissolved in anhydrous diethyl ether (10 mL) in a two necked round-bottomed flask (50 mL) fitted with a double-walled condenser, under an inert atmosphere at room temperature. Then, *n*-BuLi (1.2 equiv) was added drop-wise to the mixture using a glass syringe at the same temperature over a period of 10 min. The reaction mixture was further refluxed for 3 h on a preheated oil bath and allowed to cool to ambient temperature. Anhydrous DMF (1.5 equiv) was added to the *N*-substituted 2-lithiated indole anion and refluxed further for another 5 h. Saturated ammonium chloride (20 mL) was added to the reaction mixture at room temperature, and the aqueous layer was extracted with EtOAc (3 × 10 mL). The combined organic layers were dried over anhydrous Na_2SO_4, filtered, and concentrated under reduced pressure. The crude residue was purified using hexane/EtOAc as eluent over silica gel column chromatography to afford compounds **1a, 1b**, **1e**, and **1f**.

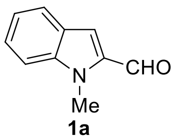

1a

1-Methyl-1H-indole-2-carbaldehyde[1] (**1a**). According to the general procedure mentioned above, 1-methyl-1*H*-indole (**12a**, 1.0 g, 7.62 mmol) was dissolved in dry diethyl ether (10 mL) under an inert atmosphere. *n*-BuLi (5.71 mL, 9.17 mmol, 1.6 M in hexane) was added drop-wise at room temperature, and the reaction mixture was refluxed for 3 h. The reaction mixture was allowed to cool to room temperature, and DMF (0.88 mL, 11.43 mmol)

was added. The reaction mixture was further refluxed for 5 h and allowed to cool to room temperature. Saturated ammonium chloride (20 mL) was added to the reaction mixture, and the aqueous layer was extracted with EtOAc (3 × 10 mL). The combined organic layers were dried over anhydrous Na_2SO_4, filtered, concentrated, and purified by silica gel (230–400 mesh) column chromatography using hexane/EtOAc 98:2 as eluent. Yield 79% (0.946 g); Pale yellow liquid; R_f 0.50 (8:2 hexane-EtOAc); ^{1}H NMR (400 MHz, $CDCl_3$) δ 9.88 (s, 1H), 7.73 (d, J = 8.0 Hz, 1H), 7.50–7.34 (m, 2H), 7.24 (s, 1H), 7.18 (dd, J = 7.8, 7.6 Hz, 1H), 4.09 (s, 3H); ^{13}C NMR (100 MHz, $CDCl_3$) δ 182.9, 140.9, 135.7, 126.9, 126.3, 123.4, 120.9, 117.5, 110.4, 31.5.

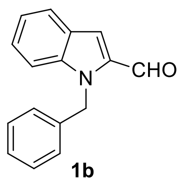

1b *1-Benzyl-1H-indole-2-carbaldehyde*[2] (**1b**). According to the general procedure, 1-benzyl-1*H*-indole (**2b**, 0.75 g, 3.62 mmol) was dissolved in dry diethyl ether (7.5 mL) under an inert atmosphere. *n*-BuLi (2.70 mL, 4.34 mmol, 1.6 M in hexane) was added drop-wise at room temperature, and the reaction mixture was refluxed for 3 h. The reaction mixture was allowed to cool to room temperature, and DMF (0.40 mL, 5.43 mmol) was added. The reaction mixture was further refluxed for 5 h and allowed to cool to room temperature. Saturated ammonium chloride (20 mL) was added, and the aqueous layer was extracted with EtOAc (3 × 10 mL). The combined organic layers were dried over anhydrous Na_2SO_4, filtered, concentrated, and purified by silica gel (230–400 mesh) column chromatography using hexane/EtOAc 98:2 as eluent. Yield 83% (0.710 g); Yellow liquid; R_f 0.60 (8:2 hexane-EtOAc); ^{1}H NMR (400 MHz, $CDCl_3$) δ 9.90 (s, 1H), 7.76 (d, J = 8.0 Hz, 1H), 7.42–7.34 (m, 2H), 7.33 (s, 1H), 7.28–7.14 (m, 4H), 7.12–7.05 (m, 2H), 5.83 (s, 2H); ^{13}C NMR (100 MHz, $CDCl_3$) δ 182.7, 140.7, 137.8*, 135.4, 128.6, 127.4, 127.2, 126.6, 123.5, 121.2, 118.3, 111.1, 48.0.

*Higher intensity carbon

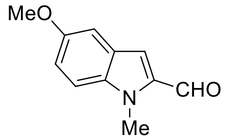

1e *5-Methoxy-1-methyl-1H-indole-2-carbaldehyde* (**1e**). According to the general procedure, 5-methoxy-1-methyl-1*H*-indole (**12e**, 0.200 g, 1.24 mmol) was dissolved in dry diethyl ether (5 mL) under an inert atmosphere. *n*-BuLi (0.93 mL, 1.49 mmol, 1.6 M in hexane) was added drop-wise at room temperature, and the reaction mixture was refluxed for

3 h. The reaction mixture was allowed to cool to room temperature, and DMF (0.14 mL, 1.86 mmol) was added to the reaction mixture. The reaction mixture was further refluxed for 5 h and cooled to ambient temperature. Saturated ammonium chloride (10 mL) was added and the aqueous layer was extracted with EtOAc (3 × 10 mL). The combined organic layers were dried over anhydrous Na_2SO_4, filtered, concentrated, and purified by silica gel (230–400 mesh) column chromatography using hexane/EtOAc 90:10 as eluent. Yield 58% (0.137 g); Off white solid; R_f 0.35 (8:2 hexane-EtOAc); ^{1}H NMR (400 MHz, $CDCl_3$) δ 9.83 (s, 1H), 7.28 (d, J = 8.8 Hz, 1H), 7.13 (s, 1H), 7.12–7.06 (m, 2H), 4.05 (s, 3H), 3.85 (s, 3H); ^{13}C NMR (100 MHz, $CDCl_3$) δ 182.7, 154.8, 136.6, 135.9, 126.5, 119.0, 116.5, 111.4, 102.7, 55.7, 31.7.

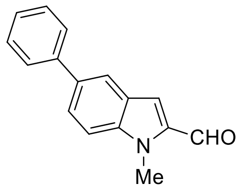

1f

1-Methyl-5-phenyl-1H-indole-2-carbaldehyde (**1f**). According to the general procedure, 1-methyl-5-phenyl-1*H*-indole (**12f**, 0.13 g, 0.63 mmol) was dissolved in dry diethyl ether (5 mL) under an inert atmosphere. *n*-BuLi (0.47 mL, 0.75 mmol, 1.6 M in hexane) was added drop-wise at room temperature, and the reaction mixture was refluxed for 3 h and allowed to cool to room temperature. DMF (0.07 mL, 0.95 mmol) was added to the reaction mixture and reflux continued for further 5 h. Saturated ammonium chloride solution (10 mL) was added to the mixture after cooling to room temperature. The aqueous layer was extracted with EtOAc (3 × 10 mL), and the combined organic layers were dried over anhydrous Na_2SO_4. The organic layer was filtered, concentrated, and purified by silica gel (230–400 mesh) column chromatography using hexane/EtOAc 96:4 as eluent. Yield 68% (0.100 g); White solid; R_f 0.50 (9:1 hexane-EtOAc); ^{1}H NMR (400 MHz, $CDCl_3$) δ 9.90 (s, 1H), 7.95–7.88 (m, 1H), 7.69 (dd, J = 8.8, 1.5 Hz, 1H), 7.66–7.59 (m, 2H), 7.50–7.41 (m, 3H), 7.34 (dd, J = 7.5, 7.3 Hz, 1H), 7.29 (s, 1H), 4.12 (s, 3H); ^{13}C NMR (100 MHz, $CDCl_3$) δ 182.9, 141.5, 140.4, 136.3, 134.5, 128.9, 127.3, 127.0, 126.9, 126.8, 121.5, 117.7, 110.7, 31.8.

B. Synthesis of N-substituted indole-2-carboxaldehyde derivatives 1d and 1g

(a) General procedure for the synthesis of N-substituted ethyl-1*H*-indole-2-carboxylate **14d** and **14g**

In a round-bottomed flask (50 mL), sodium hydride (NaH, 2.0 equiv) was added to dry DMF (2 mL), and the suspension was cooled to 0 °C under an inert atmosphere. Ethyl indole-2-

carboxylate (**13**, 1.0 equiv) was added to the suspension in one portion and the reaction mixture was stirred for 5 min at the same temperature. Alkyl or tosyl halide (1.5 equiv) was added drop-wise to the reaction mixture via a glass syringe, and the reaction mixture was allowed to warm to room temperature and stirred until **13** was consumed completely. EtOAc (5 mL) was added to the reaction mixture, followed by cold brine (5 mL). The aqueous layer was extracted with EtOAc (3 × 5 mL), and the combined organic layers were dried over anhydrous Na_2SO_4, filtered, and concentrated under reduced pressure. The crude residue was purified using hexane: EtOAc as eluent by silica gel (230–400 mesh) column chromatography.

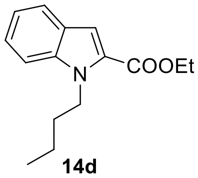

14d *Ethyl 1-butyl-1H-indole-2-carboxylate*[10] (**14d**). According to the general procedure, NaH (46 mg, 1.06 mmol, 55–60% in mineral oil) was added to dry DMF (2 mL) in a 50 mL RB flask and the suspension was cooled to 0 °C under an inert atmosphere. Ethyl indole-2-carboxylate (0.10 g, 0.53 mmol) was added to the suspension in one portion and the reaction mixture was stirred for 5 min at the same temperature. *n-Butyl iodide* (91 µL, 0.80 mmol) was added drop-wise and the reaction mixture stirred at room temperature for further 5 min. After the completion of the reaction as confirmed by TLC, EtOAc (5 mL) was added, followed by cold brine solution (5 mL). The aqueous layer was extracted with EtOAc (3 × 5 mL) and the combined organic layers were dried over anhydrous Na_2SO_4. The organic layer was filtered, concentrated, and purified by silica gel (230–400 mesh) column chromatography using hexane/EtOAc 98:2 as eluent. Yield 98% (0.125 g); Colorless liquid; R_f 0.45 (9:1 hexane-EtOAc); ^{1}H NMR (400 MHz, $CDCl_3$) δ 7.67 (d, J = 8.0 Hz, 1H), 7.40 (d, J = 7.8 Hz, 1H), 7.36–7.28 (m, 2H), 7.14 (dd, J = 7.8, 7.0 Hz, 1H), 4.57 (t, J = 7.5 Hz, 2H), 4.38 (q, J = 7.0 Hz, 2H), 1.86–1.71 (m, 2H), 1.45–1.31 (m, 5H), 0.95 (t, J = 7.3 Hz, 3H); ^{13}C NMR (100 MHz, $CDCl_3$) δ 162.1, 139.1, 127.5, 126.0, 124.8, 122.6, 120.4, 110.5, 110.4, 60.5, 44.6, 32.8, 20.2, 14.4, 13.9.

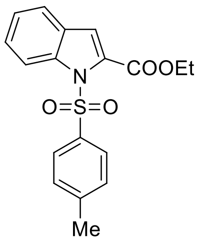

14g

Ethyl 1-tosyl-1H-indole-2-carboxylate[11] (**14g**). According to the general procedure, NaH (46 mg, 1.06 mmol, 55–60% in mineral oil,) was added to dry DMF (2 mL) in a 50 mL RB flask and the suspension was cooled to 0 °C under an inert atmosphere. Ethyl indole-2-carboxylate (100 mg, 0.53 mmol) was added in one portion and the reaction mixture was stirred for 5 min at the same temperature. Tosyl chloride (0.152 g, 0.80 mmol) was added in one portion and the reaction mixture was stirred further at room temperature for overnight. After the completion of the reaction, as confirmed by TLC, EtOAc (5 mL) was added to the reaction mixture followed by cold brine (5 mL). The aqueous layer was extracted with EtOAc (3 × 5 mL) and the combined organic layers were dried over anhydrous Na_2SO_4. The organic layer was filtered, concentrated, and purified by silica gel (230–400 mesh) column chromatography using hexane/EtOAc 90:10 as eluent. Yield 98% (0.180 g); Colorless liquid; R_f 0.50 (8:2 hexane-EtOAc); ^{1}H NMR (400 MHz, $CDCl_3$) δ 8.10 (d, J = 8.5 Hz, 1H), 7.91 (d, J = 8.0 Hz, 2H), 7.55 (d, J = 7.8 Hz, 1H), 7.42 (dd, J = 8.5, 7.8 Hz, 1H), 7.30–7.20 (m, 3H), 7.14 (s, 1H), 4.41 (q, J = 7.0 Hz, 2H), 2.36 (s, 3H), 1.39 (t, J = 7.0 Hz, 3H); ^{13}C NMR (100 MHz, $CDCl_3$) δ 161.4,144.9, 138.2, 135.7, 131.9, 129.6, 128.2, 127.4, 126.9, 124.1, 122.4, 116.5, 115.4, 62.0, 21.6, 14.1.

(b) General procedure for the synthesis of N-substituted indole-2-carboxaldehyde **1d** and **1g**

The N-substituted ethyl indole-2-carboxylate (**14d** or **14g**, 1.0 equiv) was dissolved in dry THF and cooled to 0 °C under an inert atmosphere. Lithium aluminum hydride ($LiAlH_4$, 3.0 equiv) was added in one portion to the mixture with constant stirring. The reaction mixture was allowed to warm at room temperature and continued to stir for a further 20 min. After the completion of the reaction, saturated ammonium chloride solution (5 mL) was added cautiously to quench the reaction. The aqueous layer was extracted with EtOAc (3 × 10 mL), and the combined organic layers were dried over anhydrous Na_2SO_4. The organic layer was filtered, concentrated, and used in the next step without further purification. The crude residue was dissolved in dichloromethane (10 mL), MnO_2 (15.0 equiv) was added in one portion, and the reaction mixture was refluxed for 12–48 h. After the completion of the reaction, CH_2Cl_2 was evaporated under reduced pressure, and the crude residue was purified

through silica gel (230–400 mesh) column chromatography using hexane/EtOAc mixture as eluent.

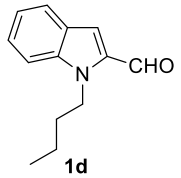

1d *1-Butyl-1H-indole-2-carbaldehyde*[3] (**1d**). According to the general procedure, **14d** (125 mg, 0.51 mmol) was dissolved in dry THF (3 mL) in a round-bottomed flask (50 mL) under an inert atmosphere. The solution was cooled to 0 °C, and $LiAlH_4$ (59 mg, 1.53 mmol) was added in one portion to the reaction mixture. The reaction mixture was allowed to warm to room temperature and stirred for another 20 min. After the completion of the reaction, saturated ammonium chloride solution (5 mL) was added cautiously to quench the reaction. The aqueous layer was extracted with EtOAc (3 × 10 mL), and the combined organic layers were dried over anhydrous Na_2SO_4. The organic layer was filtered, concentrated, and used in the next step without further purification. The crude residue was dissolved in dichloromethane (10 mL), MnO_2 (652 mg, 7.50 mmol) was added in one portion, and the reaction mixture was refluxed for 24 h. After the completion of the reaction, CH_2Cl_2 was evaporated under reduced pressure, and the crude residue was purified through silica gel (230–400 mesh) column chromatography using hexane/EtOAc 90:10 as eluent. Yield 98% (100 mg); White gummy solid; R_f 0.55 (4:1 hexane-EtOAc); ^{1}H NMR (400 MHz, $CDCl_3$) δ 9.80 (s, 1H), 7.66 (d, J = 8.0 Hz, 1H), 7.38–7.29 (m, 2H), 7.18 (s, 1H), 7.13–7.05 (m, 1H), 4.49 (t, J = 7.3 Hz, 2H), 1.75–1.63 (m, 2H), 1.34–1.22 (m, 2H), 0.86 (t, J = 7.3 Hz, 3H); ^{13}C NMR (100 MHz, $CDCl_3$) δ 181.5, 139.3, 134.4, 125.7, 125.4, 122.4, 119.8, 116.8, 109.7, 43.5, 31.6, 19.1, 12.8.

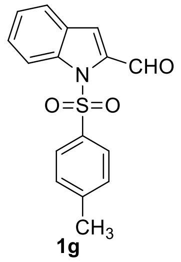

1g *1-Tosyl-1H-indole-2-carboxylate*[4] (**1g**). According to the general procedure, **14g** (180 mg, 0.52 mmol) was dissolved in dry THF (3 mL) in a round-bottomed flask (50 mL) under an inert atmosphere. The solution was cooled to 0 °C, and $LiAlH_4$ (60 mg, 1.56 mmol) was added in one portion, and the reaction mixture was allowed to warm to room temperature with constant stirring for another 20 min. After the completion of the reaction, saturated ammonium chloride solution (5 mL) was added cautiously to quench the reaction.

The aqueous layer was extracted with EtOAc (3 × 10 mL), and the combined organic layers were dried over anhydrous Na_2SO_4. The organic layer was filtered, concentrated, and used in the next step without further purification. The crude residue was dissolved in dichloromethane (10 mL), MnO_2 (649 mg, 7.46 mmol) was added in one portion, and the reaction mixture was refluxed for 48 h. After the completion of the reaction, CH_2Cl_2 was evaporated under reduced pressure, and the crude residue was purified through silica gel (230–400 mesh) column chromatography using hexane/EtOAc 90:10 as eluent. Yield 89% (132 mg); White gummy solid; R_f 0.40 (4:1 hexane-EtOAc); ^{1}H NMR (400 MHz, $CDCl_3$) δ 10.5 (s, 1H), 8.22 (d, J = 7.5 Hz, 1H), 7.65 (d, J = 8.3 Hz, 2H),7.61 (d, J = 7.3 Hz, 1H),7.51 (dd, J = 7.5, 7.5 Hz, 1H), 7.46 (s, 1H), 7.30 (dd, J = 7.5, 7.3 Hz, 1H), 7.18 (d, J = 8.3 Hz, 2H), 2.32 (s, 3H); ^{13}C NMR (100 MHz, $CDCl_3$) δ 183.4, 145.6, 138.5, 137.8, 134.7, 130.0, 128.8, 128.2, 126.7, 124.8, 123.6, 118.9, 115.4, 21.6.

C. Synthesis of N-substituted indole-2-carboxaldehydes 1c and 1h

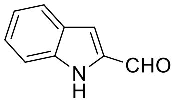

15 *1H-indole-2-carbaldehyde*[12] (**15**). Ethyl indole-2-carboxylate (**13**, 200 mg, 1.06 mmol) was dissolved in dry THF under an inert atmosphere, and the solution was cooled to 0 °C before the addition of $LiAlH_4$ (120 mg, 3.17 mmol) in a single portion. The reaction mixture was allowed to warm to room temperature and stirred for another 1 h. After the completion of the reaction, as confirmed by TLC, the reaction mixture was quenched using saturated ammonium chloride solution (10 mL), and the aqueous layer was extracted with EtOAc (3 × 10 mL). The combined organic layers were dried over anhydrous Na_2SO_4, filtered, and concentrated under reduced pressure. The crude residue was dissolved in CH_2Cl_2 (10 mL), MnO_2 (1.382 g, 15.90 mmol) was added to the solution at room temperature in one portion, and the reaction mixture was refluxed for 12 h. After the completion of the reaction, the crude reaction mixture was filtered through celite and recrystallized from 10% CH_2Cl_2 in hexane. Yield 88% (105 mg); Yellow crystalline solid; R_f 0.60 (4:1 hexane-EtOAc); ^{1}H NMR (400 MHz, $CDCl_3$) δ 9.85 (s, 1H), 9.66 (s, 1H), 7.75 (d, J = 8.0 Hz, 1H), 7.48 (d, J = 7.5 Hz, 1H), 7.40 (dd, J = 8.0, 7.3 Hz, 1H), 7.29 (s, 1H), 7.18 (dd, J = 7.5, 7.3 Hz, 1H); ^{13}C NMR (100 MHz, $CDCl_3$) δ 182.4, 138.3, 136.0, 127.43, 127.35, 123.5, 121.3, 115.3, 112.7.

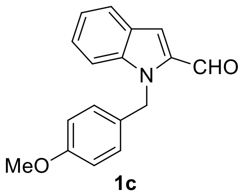

1c *1-(4-Methoxybenzyl)-1H-indole-2-carbaldehyde* (**1c**). Under an inert atmosphere, indole-2-carbaldehyde (**15**, 135 mg, 0.93 mmol) was dissolved in dry DMF (3 mL) in a single neck round-bottomed flask (50 mL). The mixture was cooled to 0 °C, and sodium hydride (56 mg, 1.40 mmol, 60% in mineral oil) was added in a single portion with stirring. The reaction mixture was allowed to warm to room temperature and stirred for further 5 minutes. 4-Methoxybenzyl chloride (250 µL, 1.86 mmol) was added drop-wise to the reaction mixture at room temperature, and the reaction was stirred for another 1 h. After the completion of the reaction, cold brine solution (5 mL) was added cautiously to quench the reaction. The aqueous layer was extracted with EtOAc (3 × 10 mL), and the combined organic layers were dried over anhydrous Na_2SO_4. The organic layer was filtered, concentrated, and purified through silica gel (230–400 mesh) column chromatography using hexane/EtOAc 95:5 as eluent. Yield 81% (200 mg); White solid; R_f 0.45 (9:1 hexane-EtOAc); ^{1}H NMR (400 MHz, $CDCl_3$) δ 9.90 (s, 1H), 7.75 (d, J = 8.2 Hz, 1H), 7.46–7.34 (m, 2H), 7.31 (s, 1H), 7.18 (dd, J = 7.8, 6.8 Hz, 1H), 7.08 (d, J = 8.5 Hz, 2H), 6.78 (d, J = 8.5 Hz, 2H), 5.76 (s, 2H), 3.73 (s, 3H); ^{13}C NMR (100 MHz, $CDCl_3$) δ 182.7, 158.9, 140.6, 135.3, 129.9, 128.1, 127.2, 126.6, 123.5, 121.2, 118.4, 114.0, 111.1, 55.2, 47.4.

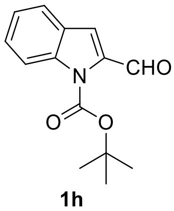

1h *tert-Butyl 2-formyl-1H-indole-1-carboxylate*[5] (**1h**). A stirred solution of indole-2-carboxaldehyde (**15**, 75 mg, 0.52 mmol), trimethylamine (86 µL, 0.62 mmol), and dimethyl aminopyridine (6 mg, 0.05 mmol) in dry THF (3 mL) was cooled to 0 °C. Boc anhydride (145 µL, 0.62 mmol) was added drop-wise to the mixture using a glass syringe under inert atmosphere. The reaction mixture was allowed to warm to room temperature and stirred overnight. After the completion of the reaction, the reaction mixture was concentrated under reduced pressure and the crude product was purified through silica gel (230–400 mesh) column chromatography using hexane/EtOAc 95:5.0 as eluent. Yield 94% (120 mg); White gummy solid; R_f 0.50 (9:1 hexane-EtOAc); ^{1}H NMR (400 MHz, $CDCl_3$) δ 10.43 (s, 1H), 8.16 (d, J = 7.8 Hz, 1H), 7.67 (d, J = 7.5 Hz, 1H), 7.48 (dd, J = 7.8, 7.8 Hz, 1H), 7.43 (s, 1H), 7.29

(dd, J = 7.8, 7.5 Hz, 1H), 1.71 (s, 9H); ^{13}C NMR (100 MHz, $CDCl_3$) δ 184.2, 149.9, 137.94, 137.9, 128.3, 127.6, 123.9, 123.2, 116.5, 116.1, 85.6, 28.2.

3. UV calibration of γ-carboline **3ac** in organic solvents

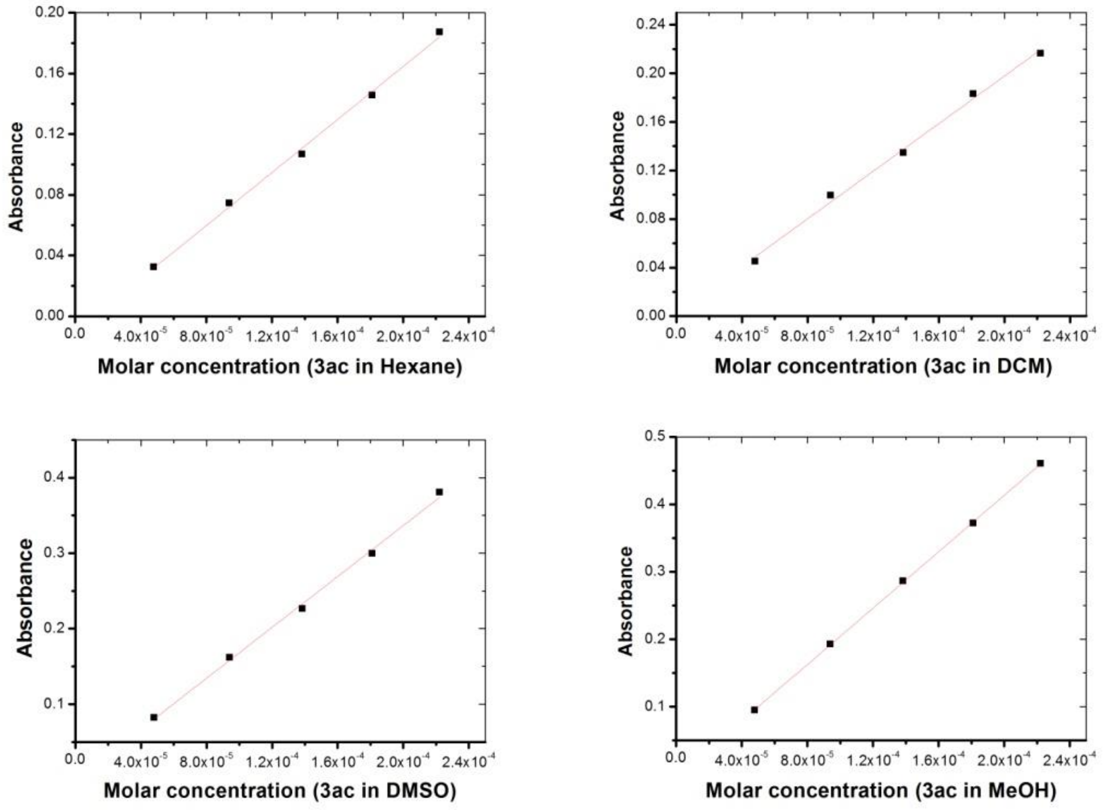

Figure S1. UV calibration curve for γ-carboline **3ac** in different solvents.

4. Single-crystal XRD analysis of **3ac**

Crystallographic data for compound **3ac** and details of X-ray diffraction experiments and crystal structure refinements are given below. Using Olex2, the structures were solved with the ShelXS structure solution program using Direct Methods and refined with the ShelXL refinement package using Least Squares minimization. All non-hydrogen atoms were refined with anisotropic displacement coefficients. Crystallographic data for the structure **3ac** has been deposited with the Cambridge Crystallographic Data Center as CCDC 1897787. Copies of the data can be obtained free of charge on application to CCDC, 12 Union Road, Cambridge CB2 1EZ, UK (fax: (+44) 1223-336-033; email: deposit@ccdc.cam.ac.uk.

Bond precision: C-C = 0.0037ÅWavelength=1.54184

Cell: a=9.8883(3)Å b=9.9509(3)Å c=22.4024(7)Å

alpha/°=90 beta/°=90.314(3) gamma/°=90

Temperature: 293 K

**Parameters**	**Calculated**	**Reported**
Volume/Å^3	2204.31(12)	2204.32(12)
Space group	P 21/n	P 1 21/n 1
Hall group	-P 2yn	-P 2yn
Empirical formula	$C_{26}H_{25}N_3O_2$	$C_{26}H_{25}N_3O_2$
Sum formula	$C_{26}H_{25}N_3O_2$	$C_{26}H_{25}N_3O_2$
Formula weight	411.49	411.49
ρcalcgcm^{-3}	1.240	1.240
Z	4	4
μ (mm^{-1})	0.632	0.632

F(000)	872.0	872.0
F(000’)	874.49	
h,k,l_{max}	12,12,28	12,12,27
N_{ref}	4582	4418
T_{min},T_{max}	0.906,0.927	0.357,1.000
$T_{min'}$	0.904	

Correction method= # Reported T Limits: T_{min}=0.357 T_{max}=1.000

AbsCorr = MULTI-SCAN

Data completeness= 0.964 Theta(max)= 75.494

R(reflections)= 0.0962 (3510) wR2(reflections)= 0.2860 (4418)

S = 1.155 N_{par}= 285

Dose–response or IC_{50} curves for the representative γ-carbolines **3ac**, **3bc**, **3ca**, and **3ga** against cancer cell lines

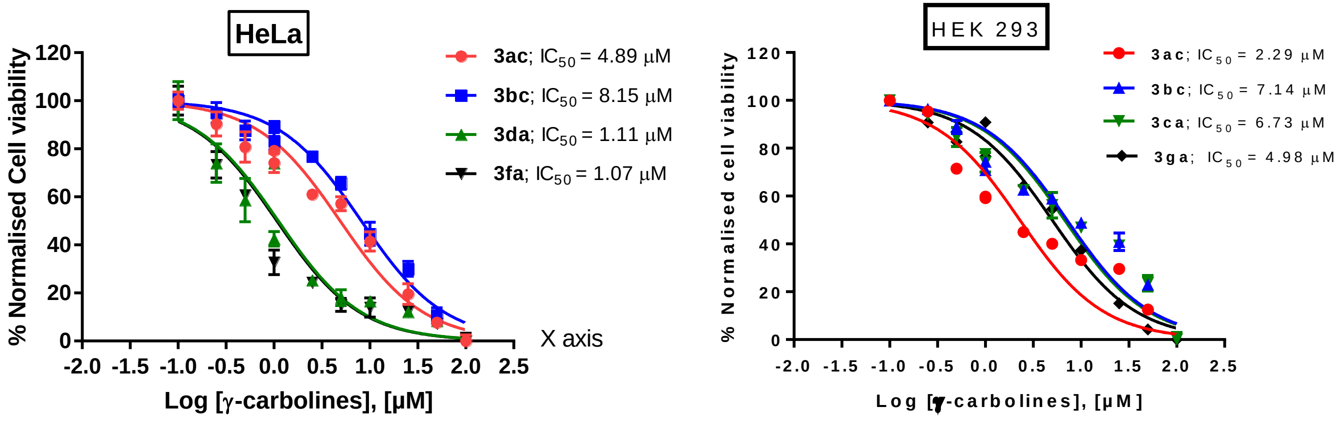

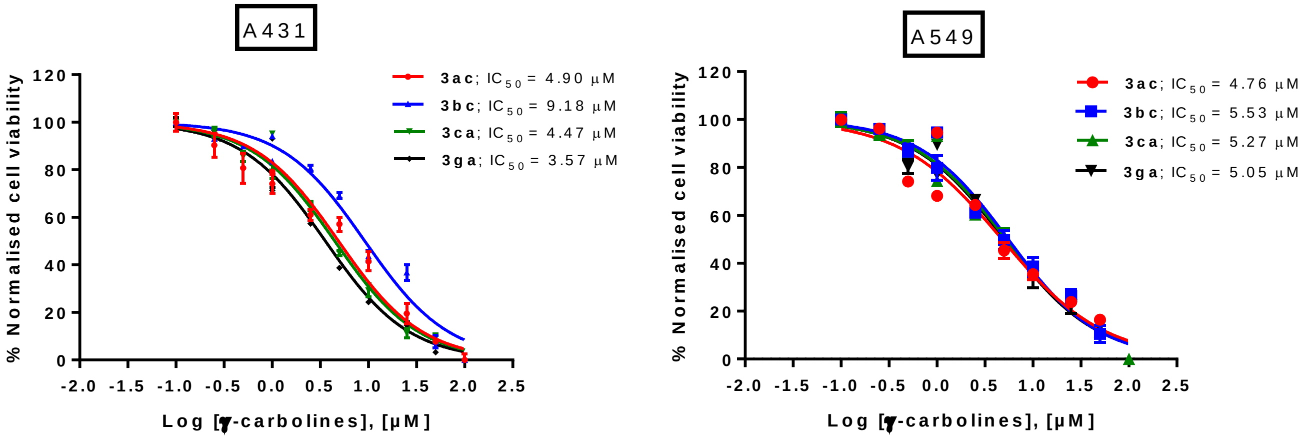

Figure S2. Dose–response or IC_{50} curves for γ-carbolines **3ac**, **3bc**, **3ca**, and **3ga** in various cancer cell lines.

5. Confocal microscopic studies in HeLa cells with 100 nM **3ac**

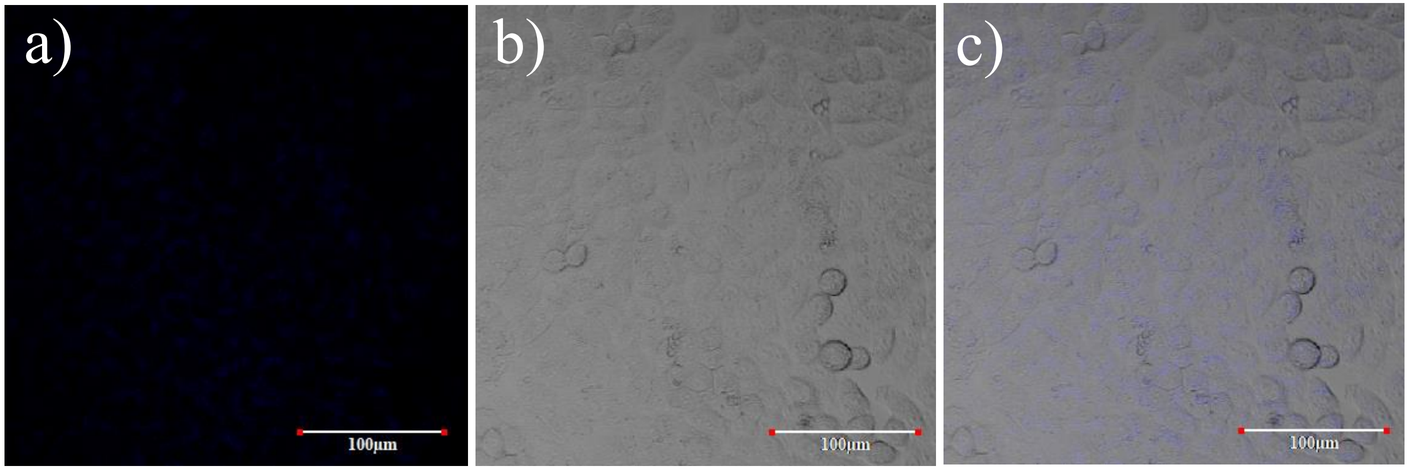

Figure S3. Laser scanning confocal microscopy studies (λ_{ex} = 405 nm; collection range = 420–470 nm) for uptake of **3ac** in HeLa cells; a) confocal image of HeLa cells after 3 h of incubation with 100 nM concentration of **3ac** (20-fold magnification, 2-fold zoom); b) DIC image of HeLa cells; c) overlay of (a) and (b) showing nominal uptake of **3ac** in cytoplasm.

Copies of ^{1}H and ^{13}C NMR spectra of 1a–h, 3aa–3ac, 3ba–3bc, 3da, 3ea, 3ga, 12a–b, 12e–f, 12i, 14d, 14g, and 15.

^{1}H NMR spectrum of *1-methyl-1H-indole-2-carbaldehyde* (**1a**)

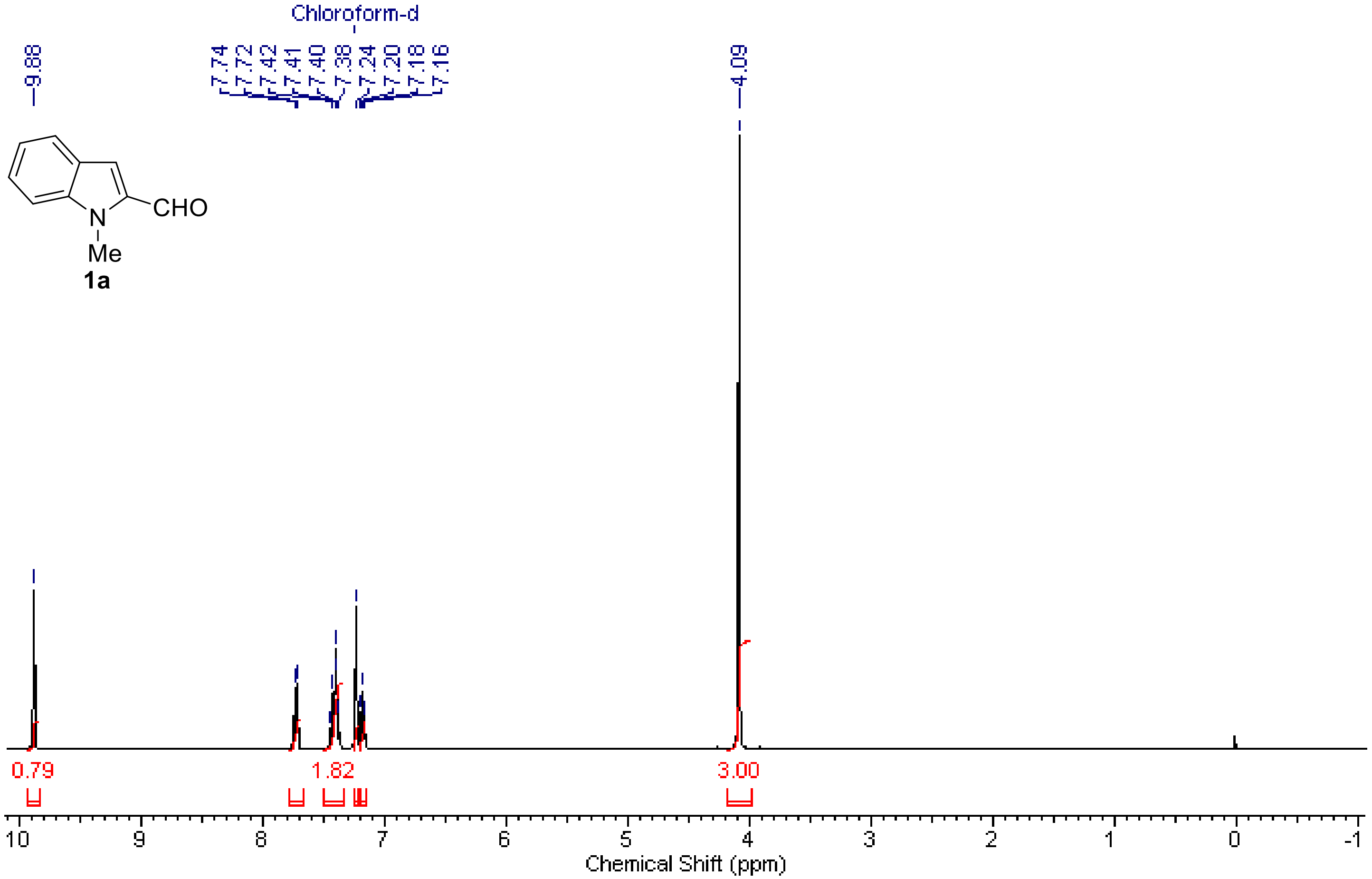

^{13}C NMR spectrum of *1-methyl-1H-indole-2-carbaldehyde* (**1a**)

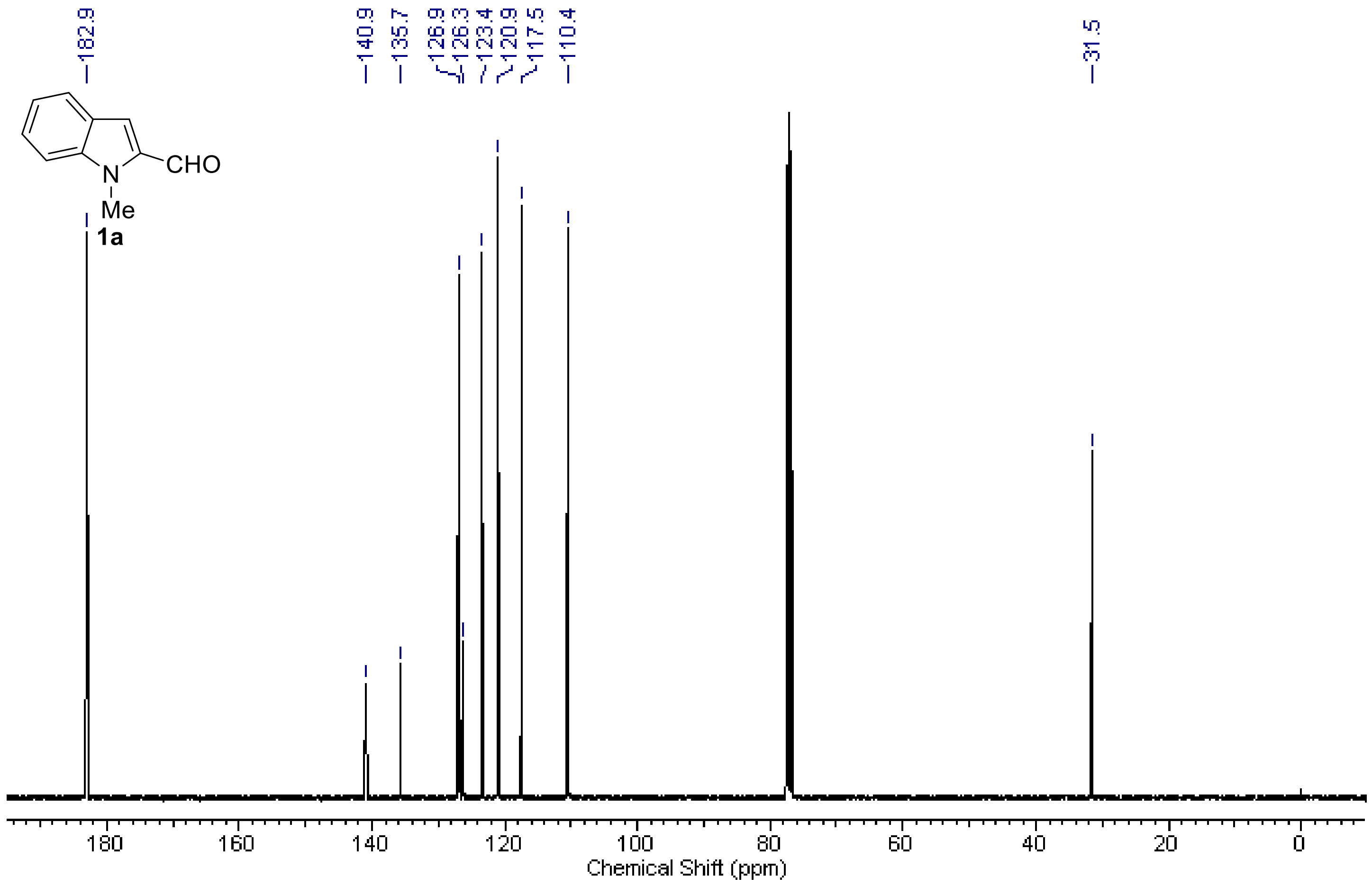

^{1}H NMR spectrum of *1-benzyl-1H-indole-2-carbaldehyde* (**1b**)

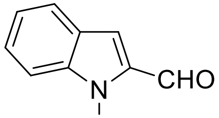

1b

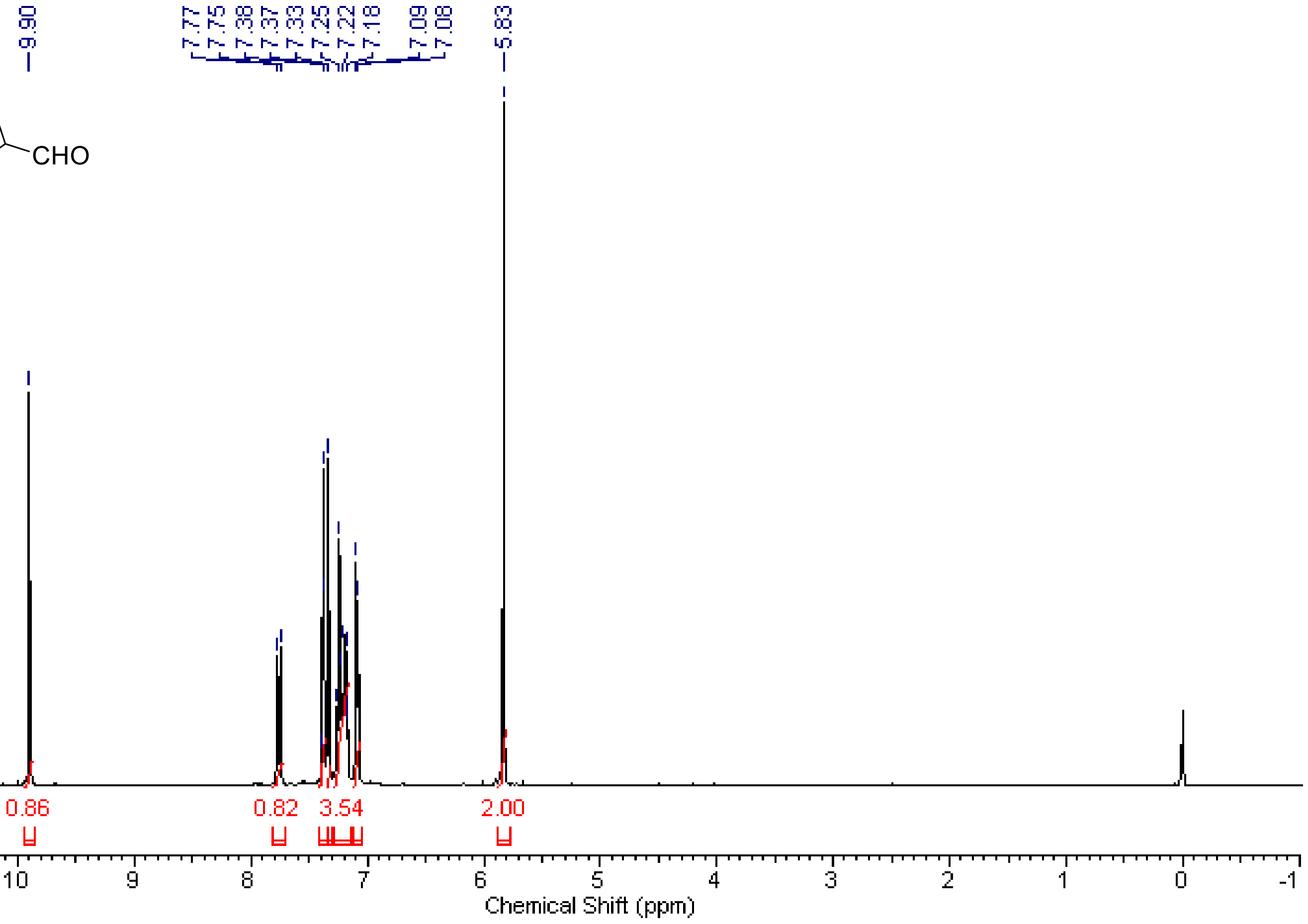

^{13}C NMR spectrum of *1-benzyl-1H-indole-2-carbaldehyde* (**1b**)

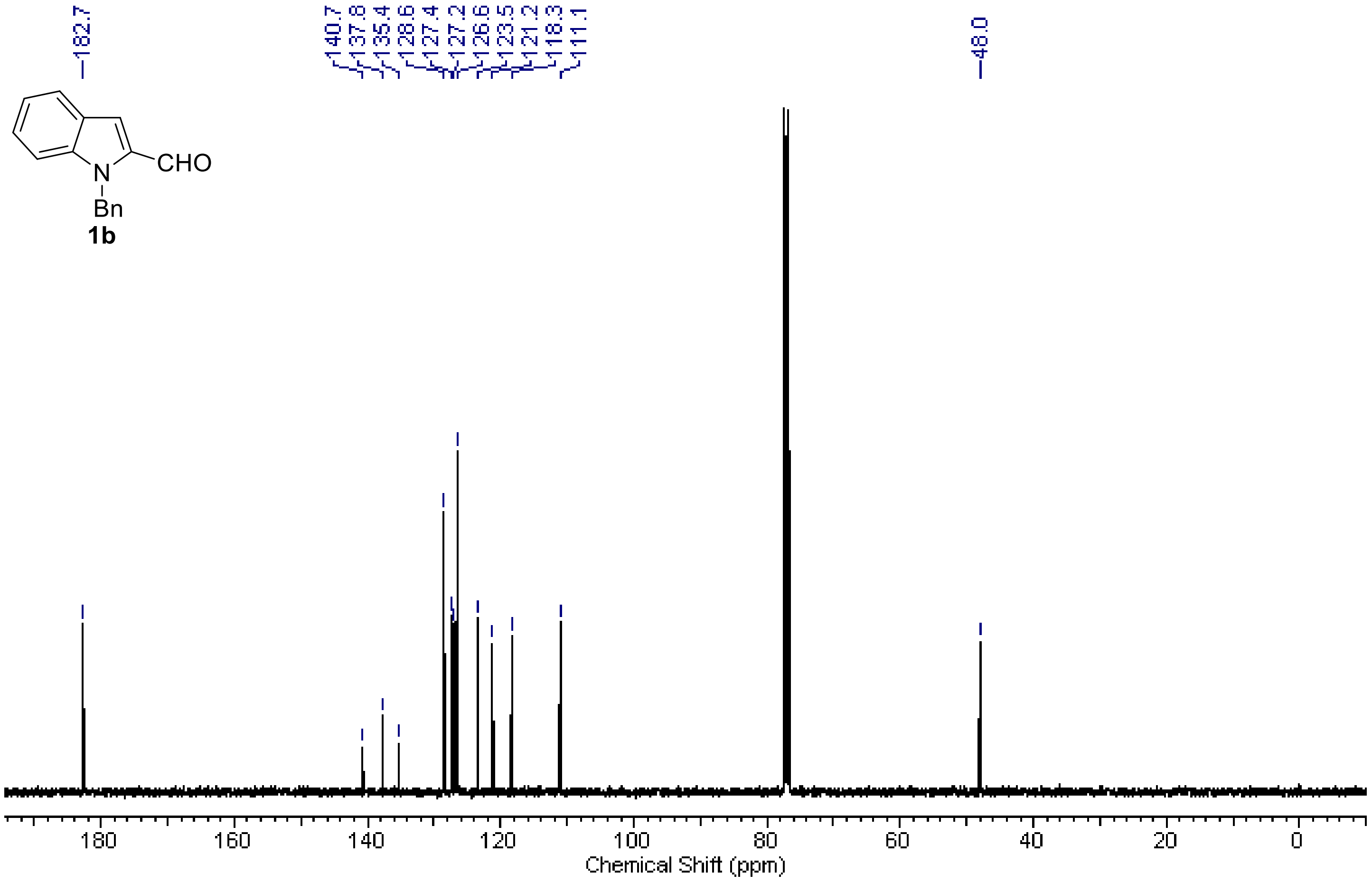

^{1}H NMR spectrum of *1-(4-methoxybenzyl)-1H-indole-2-carbaldehyde* (**1c**)

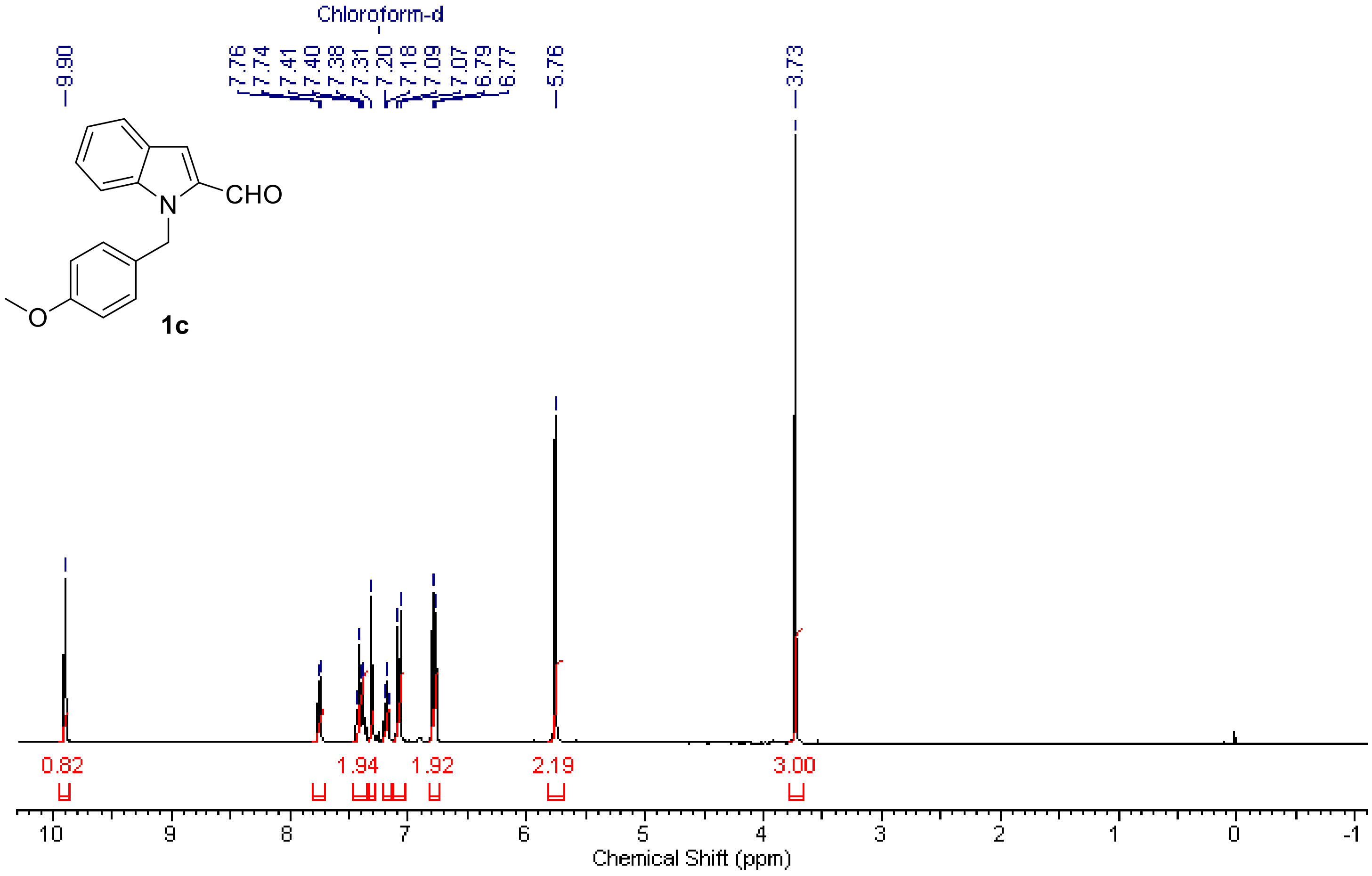

^{13}C NMR spectrum of *1-(4-methoxybenzyl)-1H-indole-2-carbaldehyde* (**1c**)

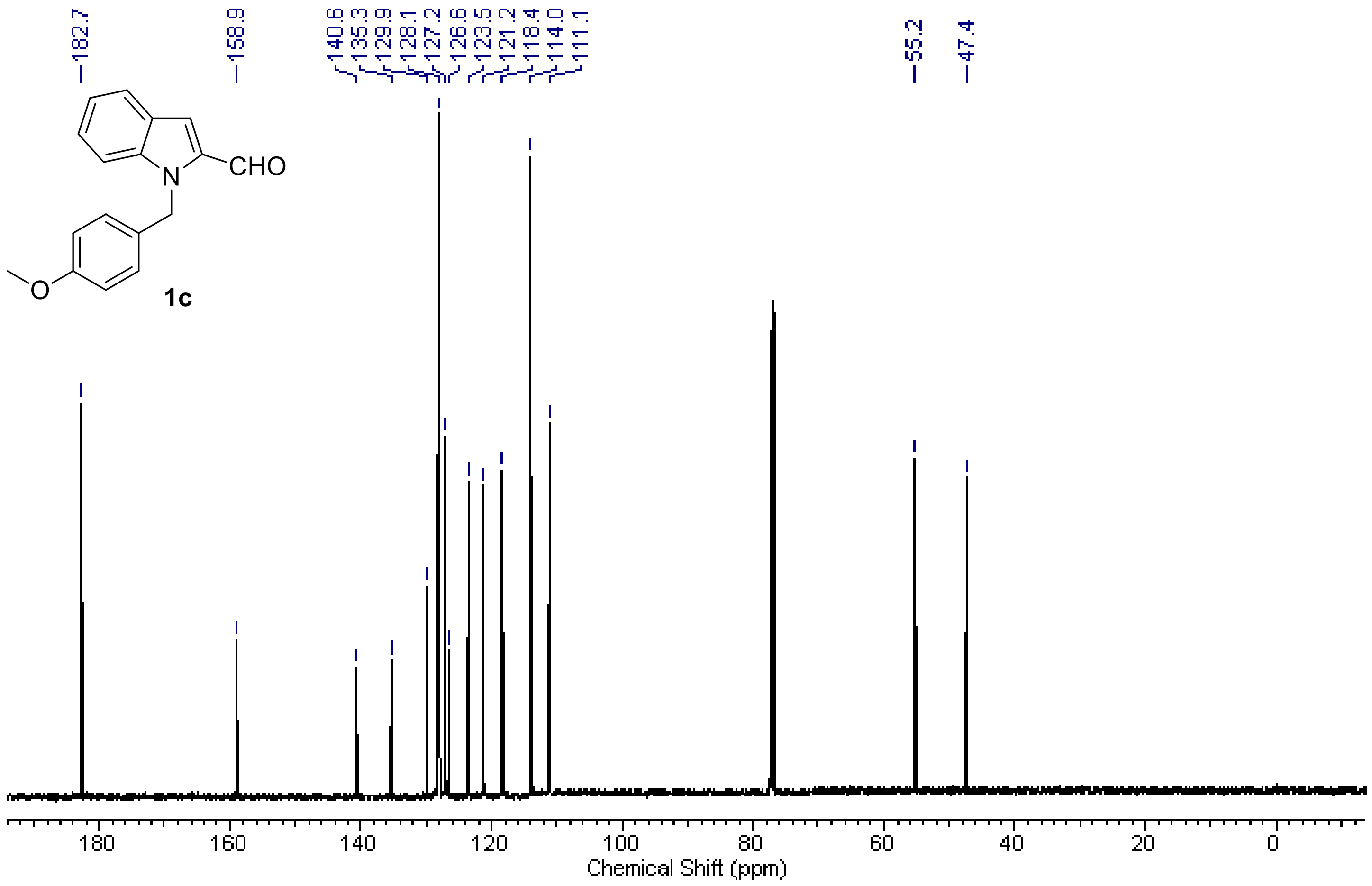

^{1}H NMR spectrum of *1-butyl-1H-indole-2-carbaldehyde* (**1d**)

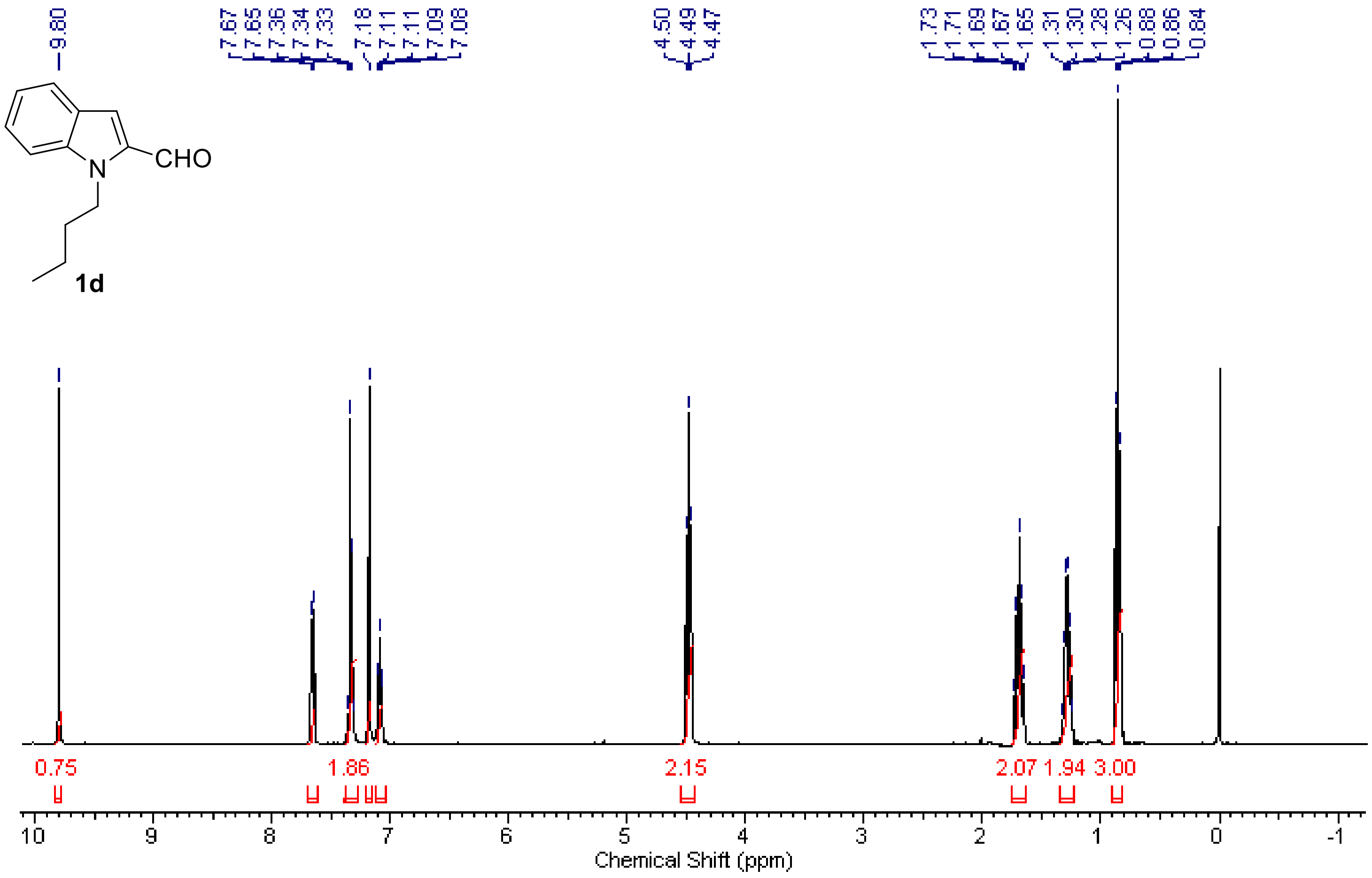

^{13}C NMR spectrum of *1-butyl-1H-indole-2-carbaldehyde* (**1d**)

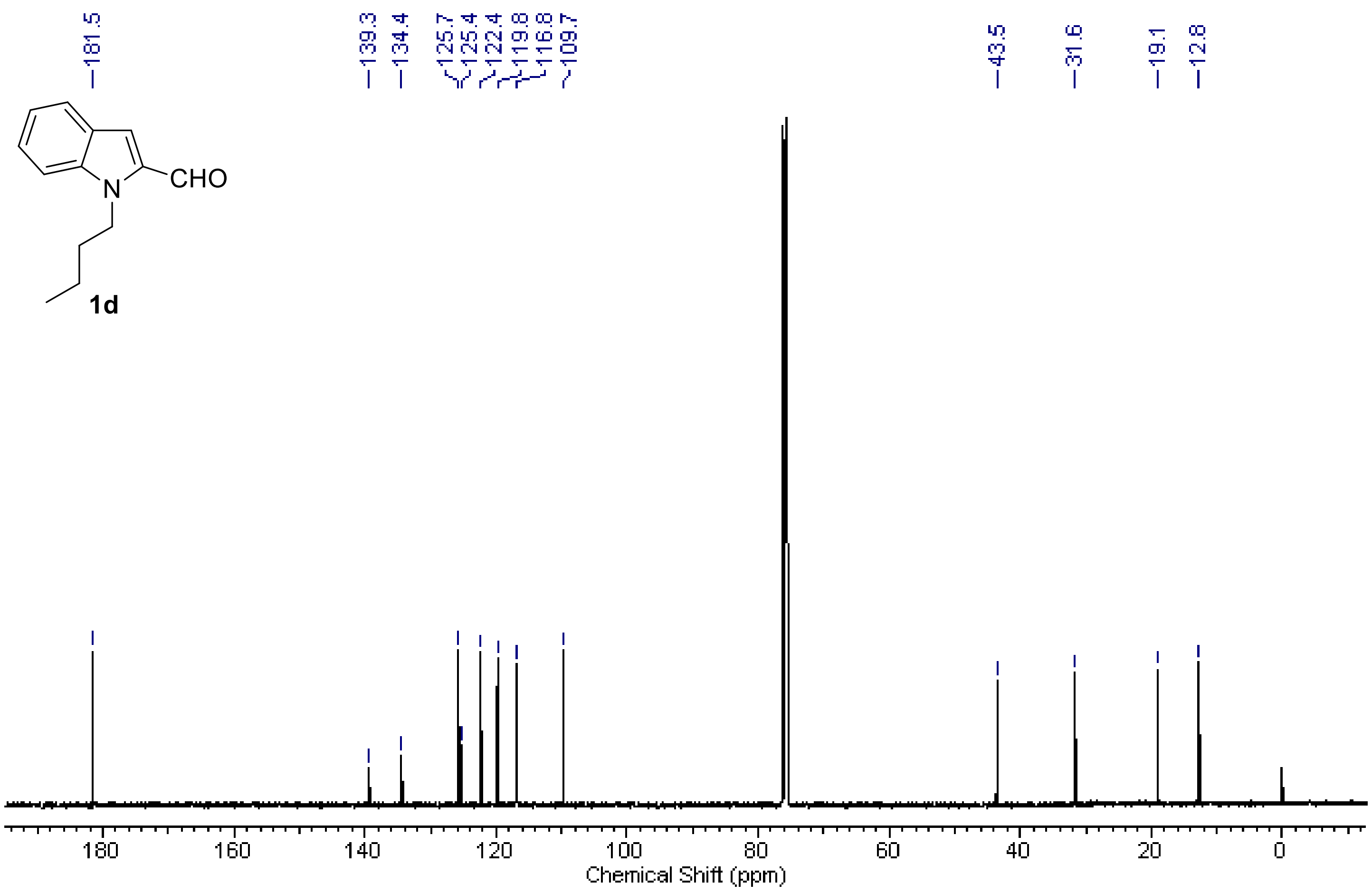

^{1}H NMR spectrum of *5-methoxy-1-methyl-1H-indole-2-carbaldehyde* (**1e**)

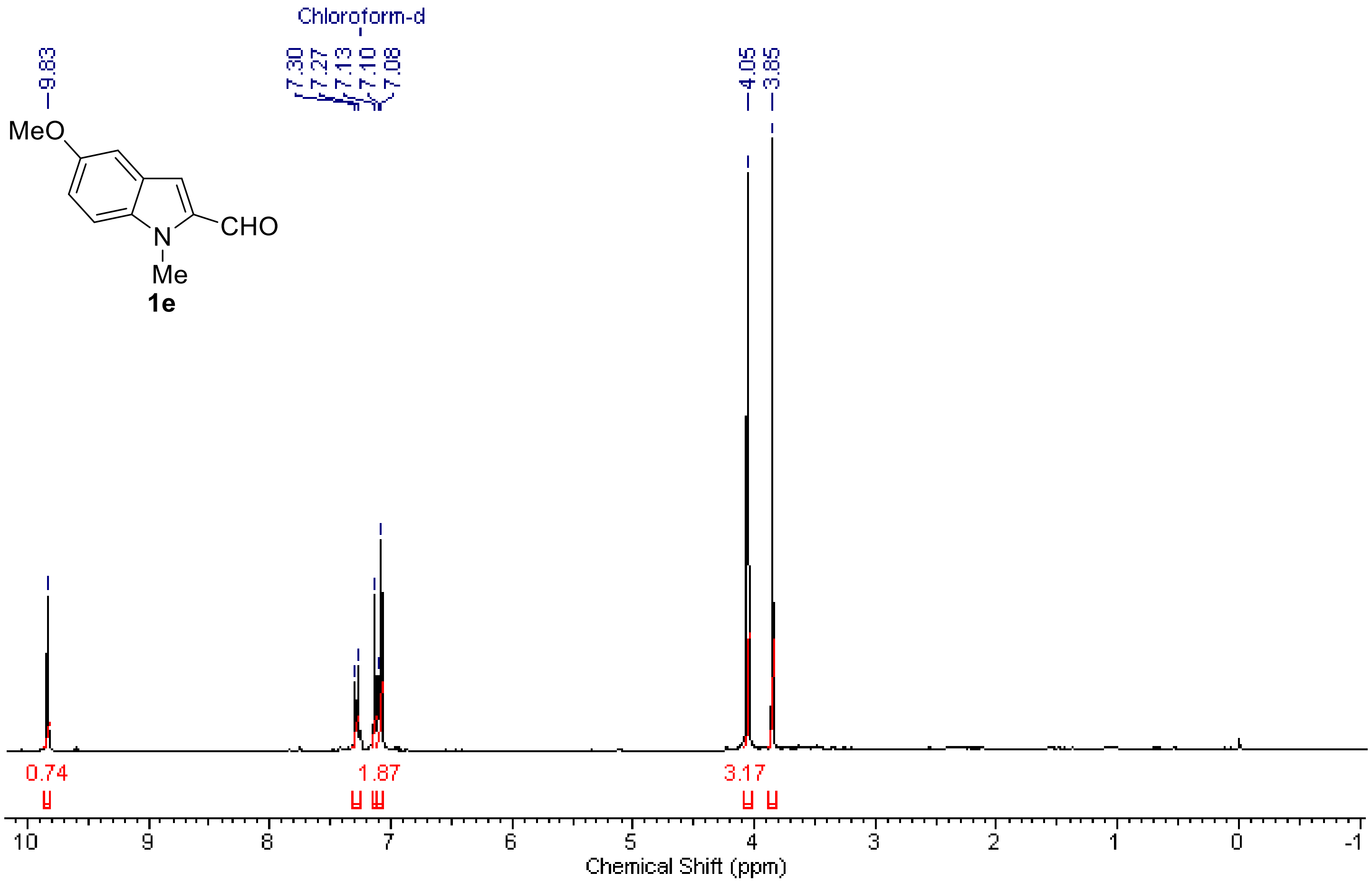

^{13}C NMR spectrum of *5-methoxy-1-methyl-1H-indole-2-carbaldehyde* (**1e**)

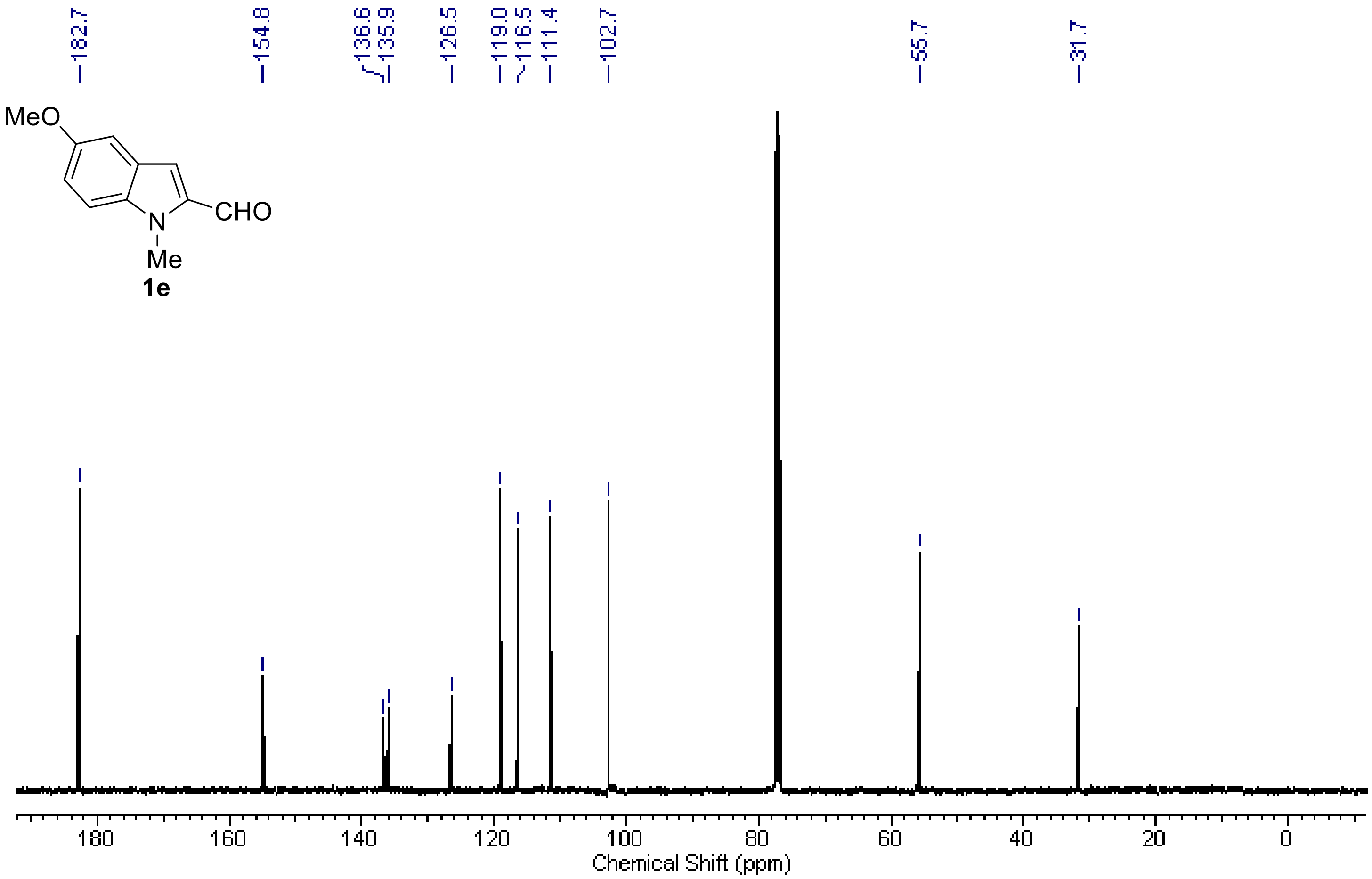

^{1}H NMR spectrum of *1-methyl-5-phenyl-1H-indole-2-carbaldehyde* (**1f**)

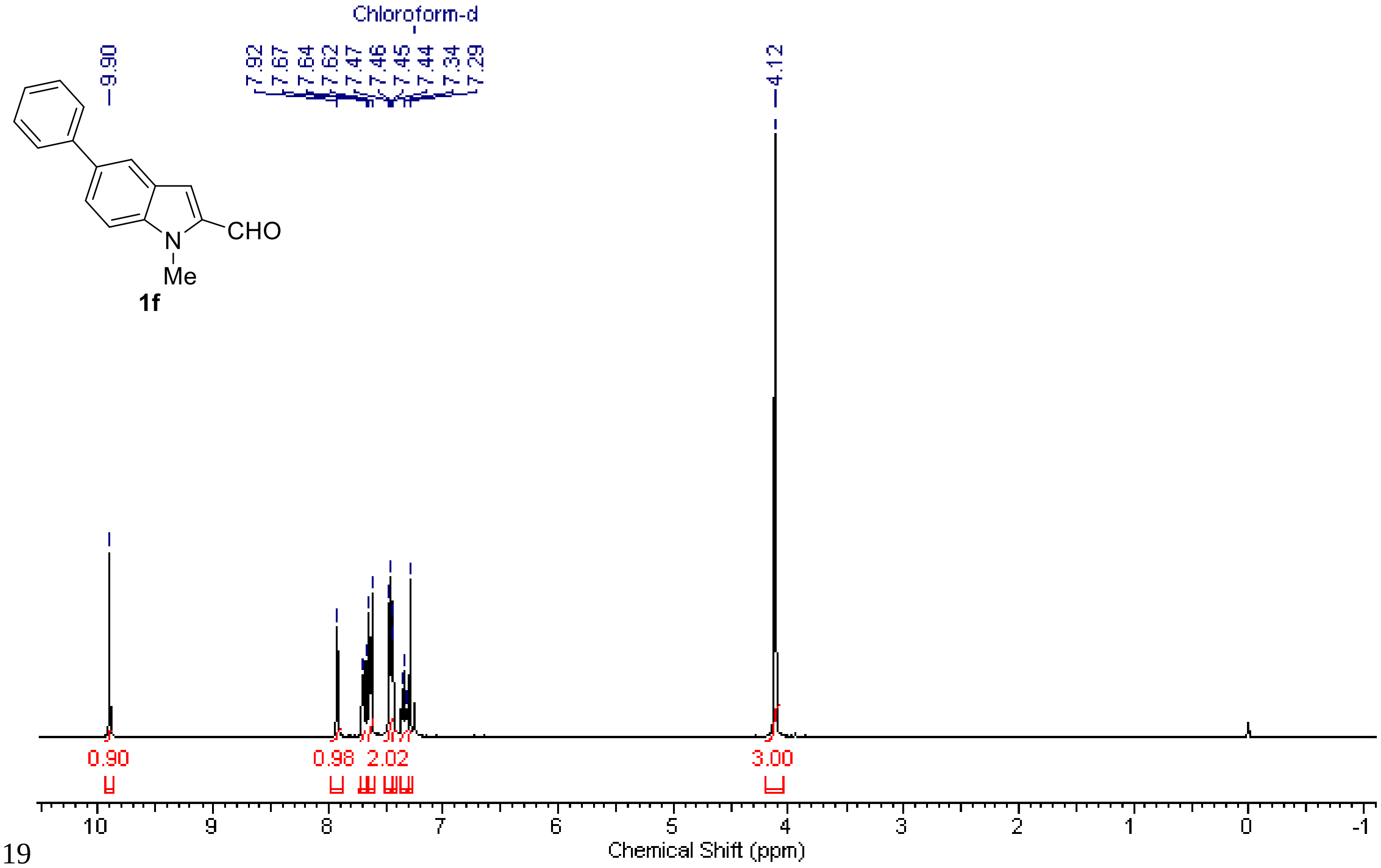

^{13}C NMR spectrum of *1-methyl-5-phenyl-1H-indole-2-carbaldehyde* (**1f**)

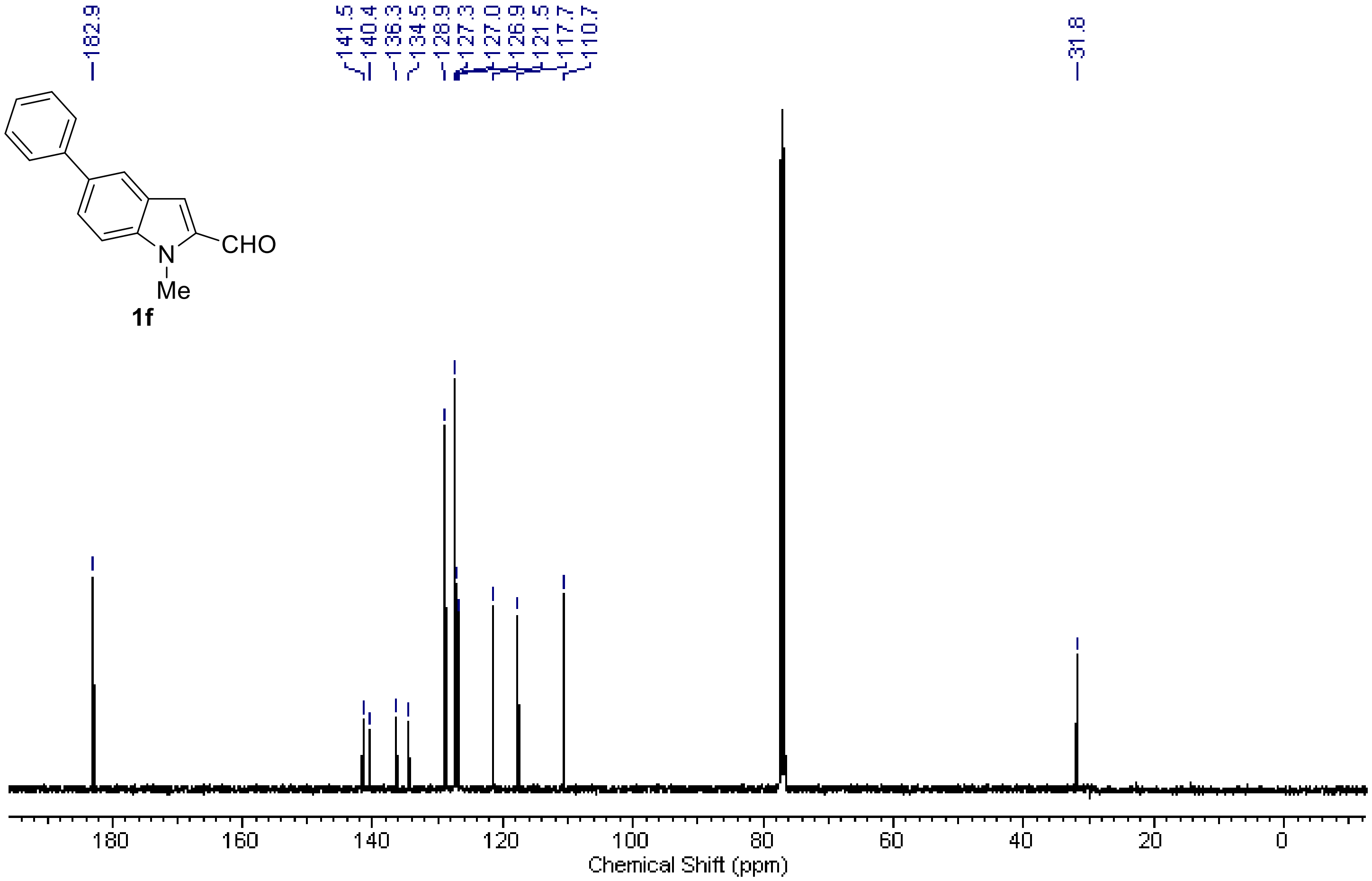

^{1}H NMR spectrum of *1-tosyl-1H-indole-2-carboxylate* (**1g**)

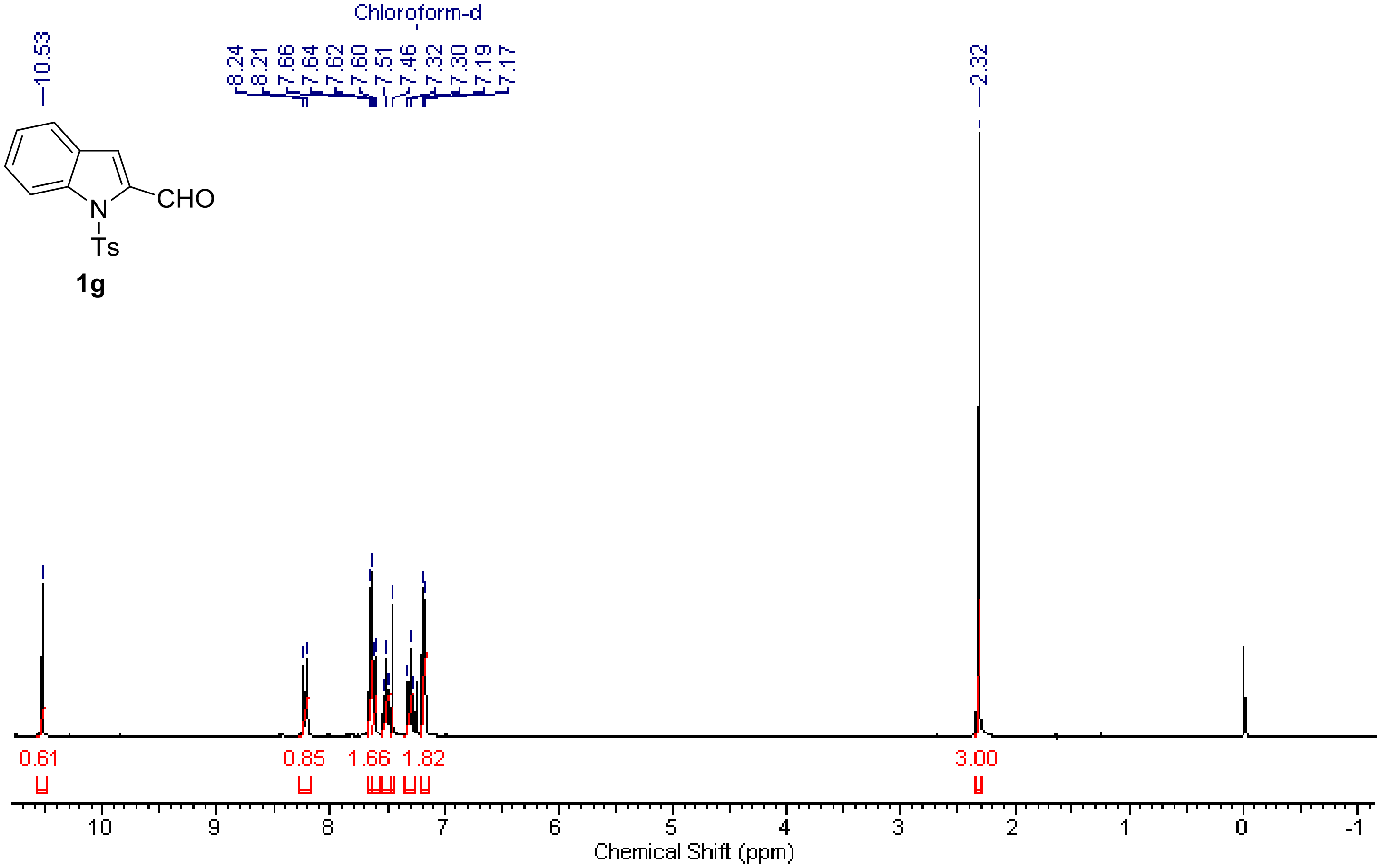

^{13}C NMR spectrum of *1-tosyl-1H-indole-2-carboxylate* (**1g**)

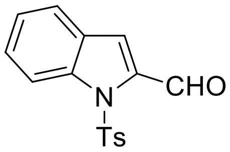

1g

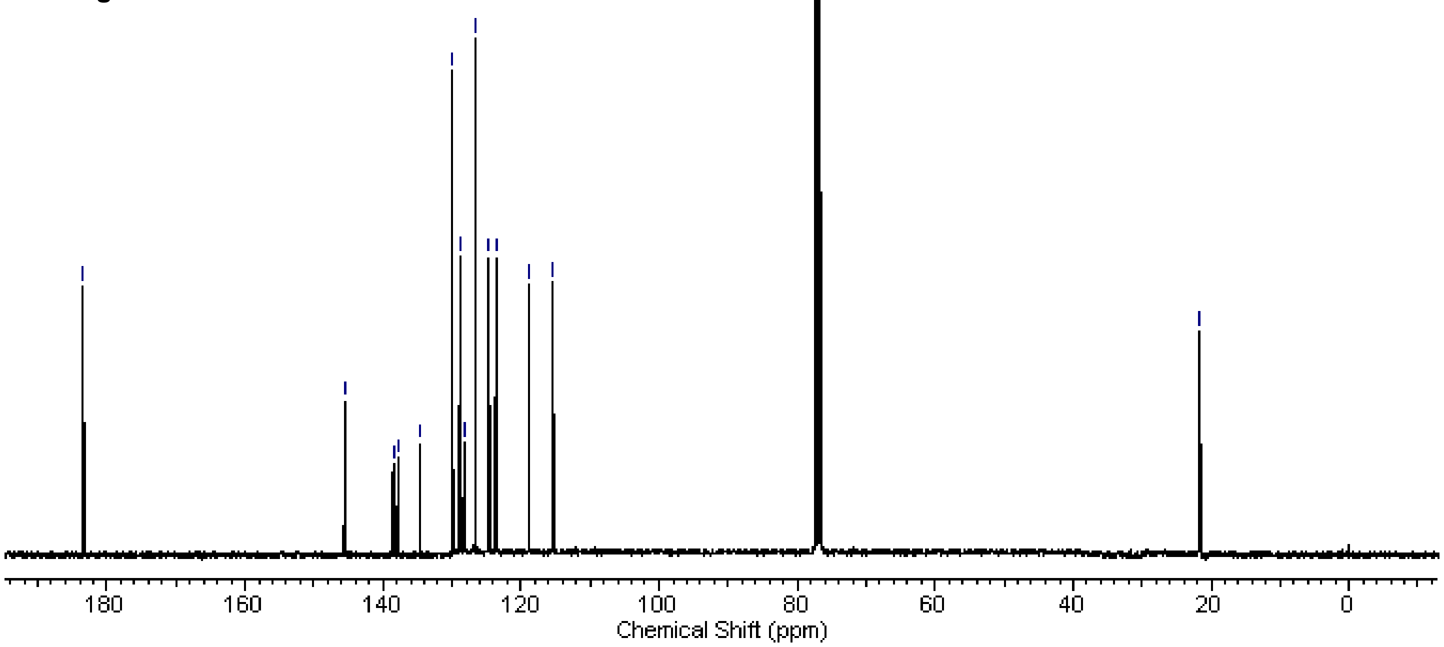

^{1}H NMR spectrum of *tert-butyl 2-formyl-1H-indole-1-carboxylate* (**1h**)

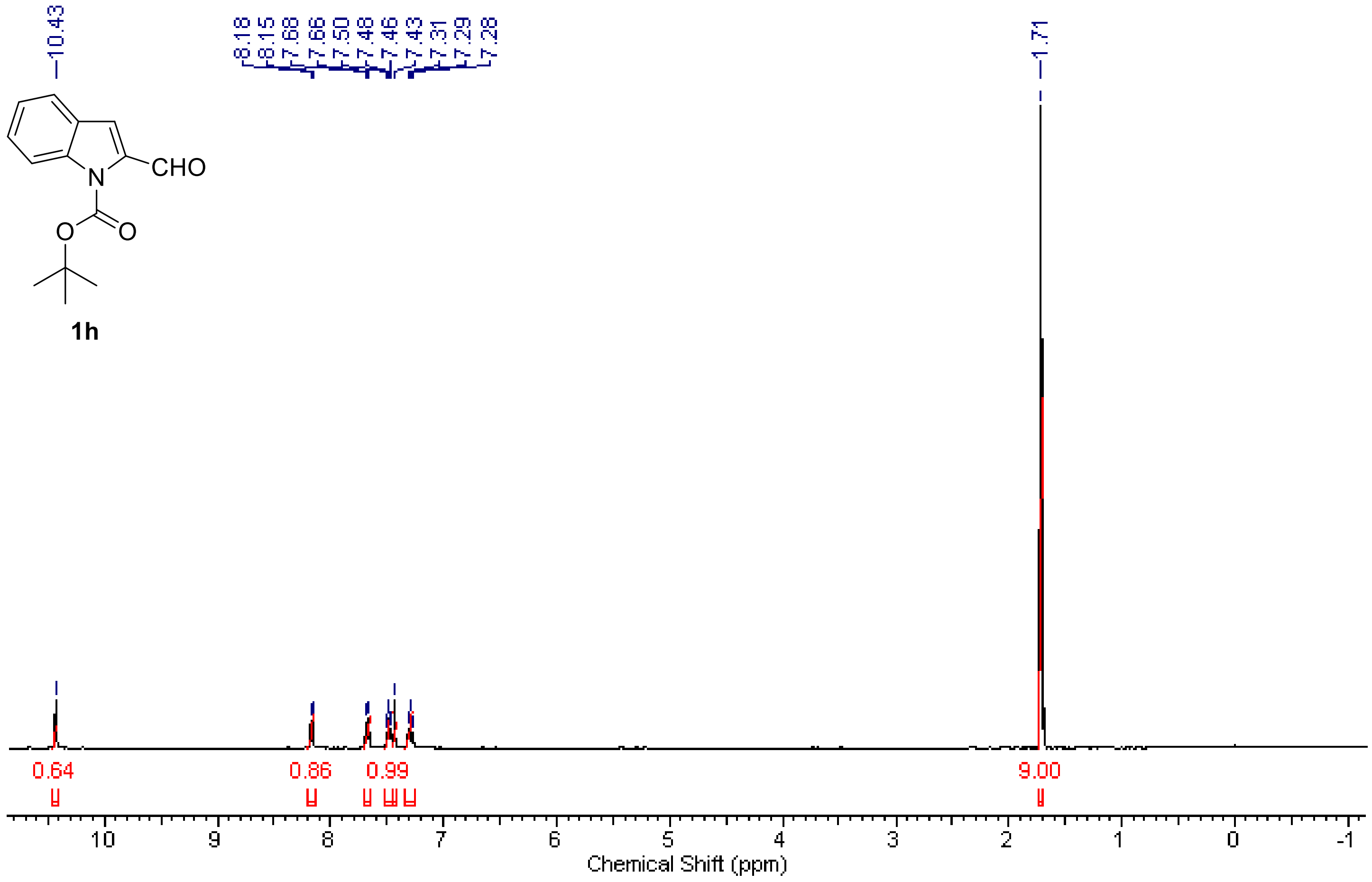

^{13}C NMR spectrum of *tert-butyl 2-formyl-1H-indole-1-carboxylate* (**1h**)

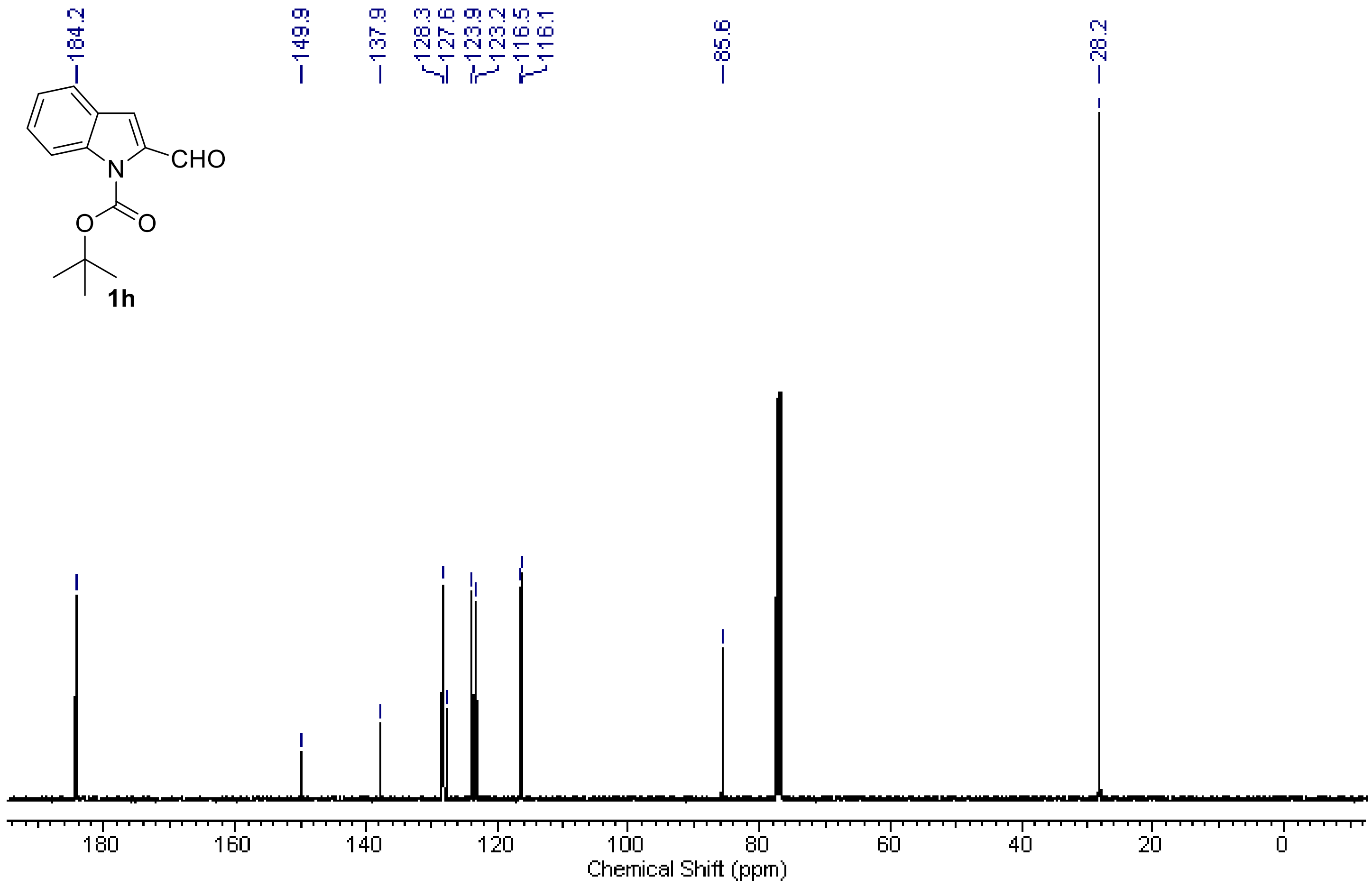

^{1}H NMR spectrum of *methyl 5-methyl-1-(1-methyl-1H-indol-2-yl)-5H-pyrido[4,3-b]indole-3-carboxylate* (**3aa**)

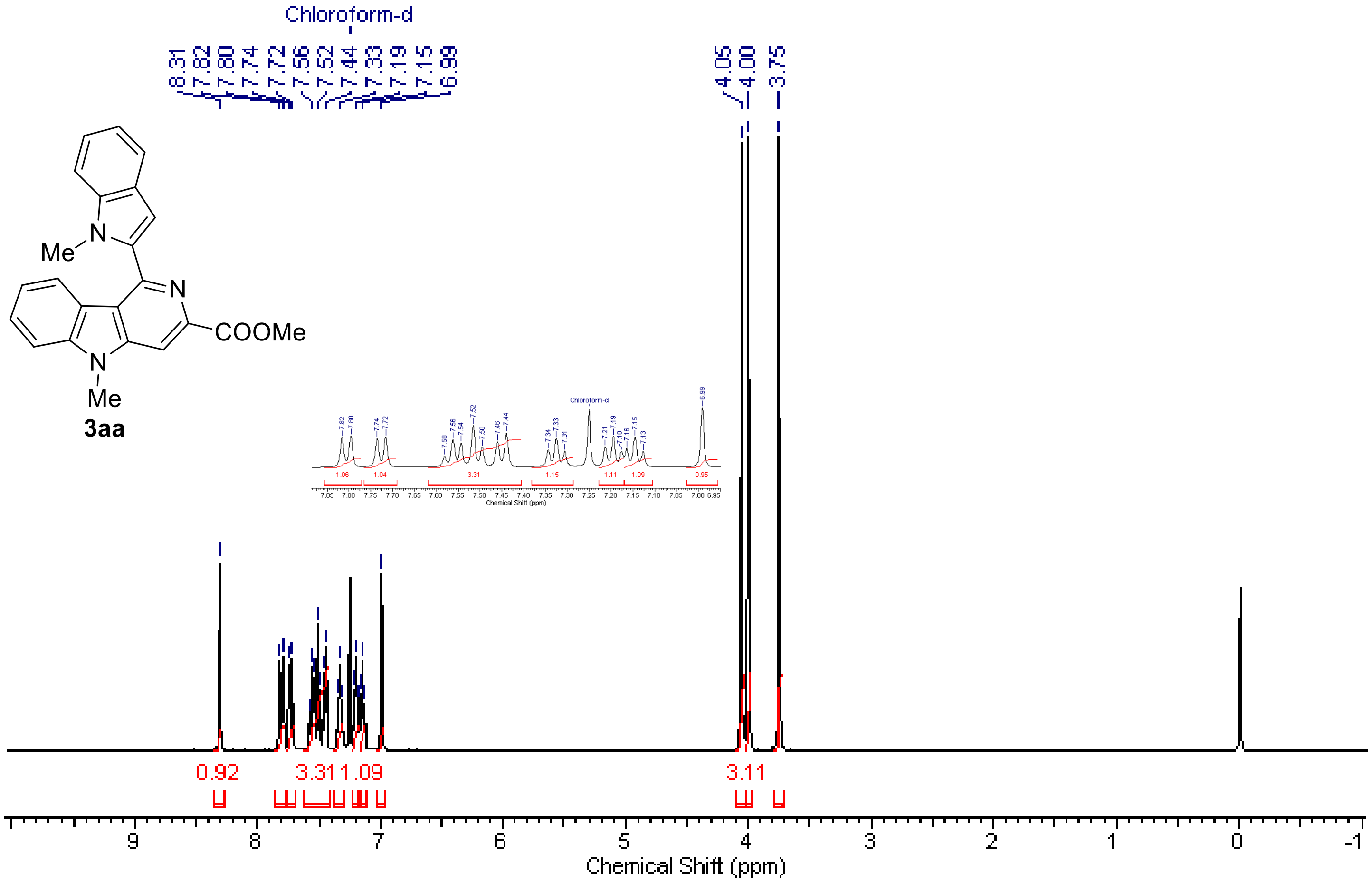

^{13}C NMR spectrum of *methyl 5-methyl-1-(1-methyl-1H-indol-2-yl)-5H-pyrido[4,3-b]indole-3-carboxylate* (**3aa**)

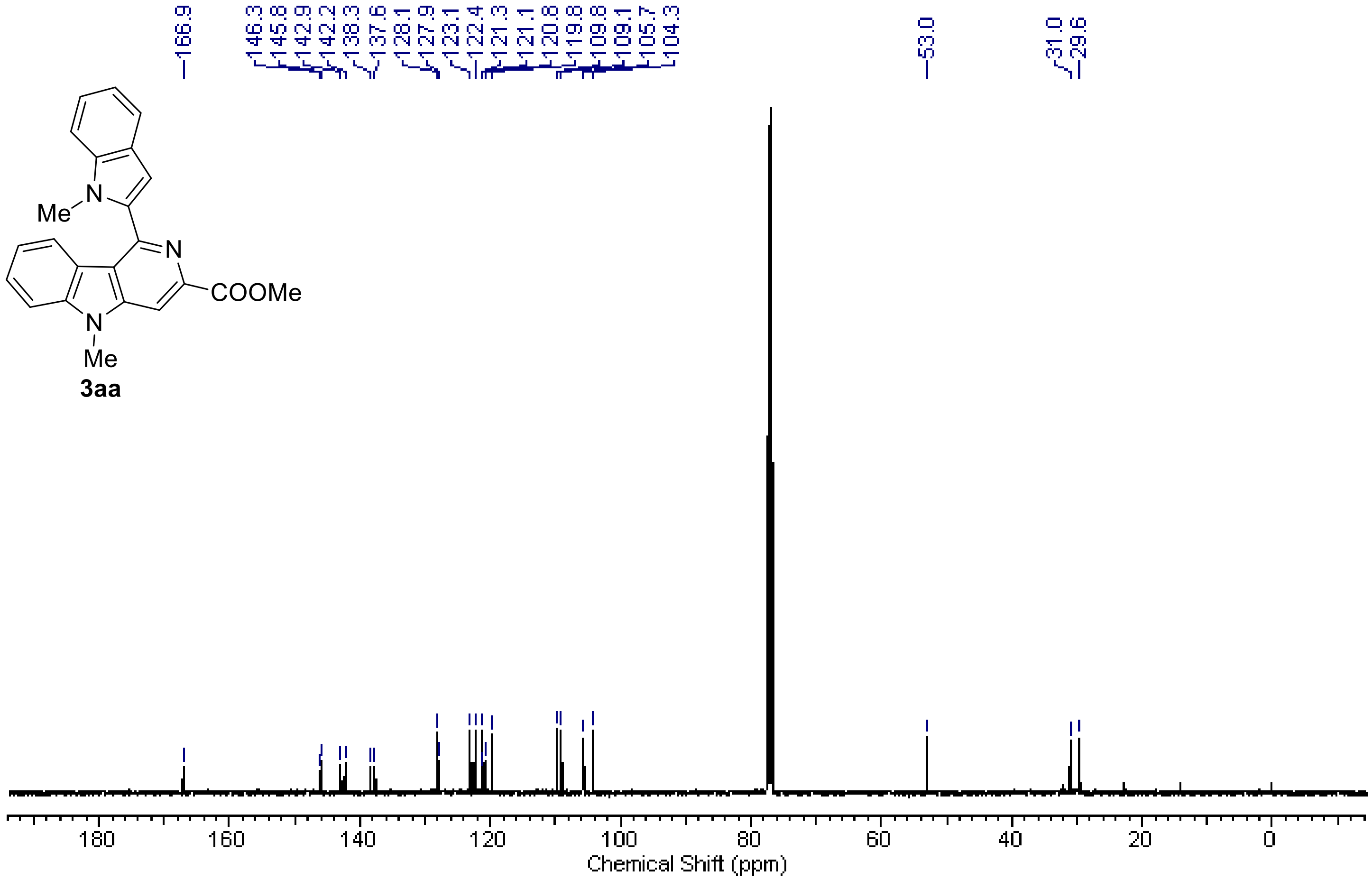

^{1}H NMR spectrum of *ethyl 5-methyl-1-(1-methyl-1H-indol-2-yl)-5H-pyrido[4,3-b]indole-3-carboxylate* (**3ab**)

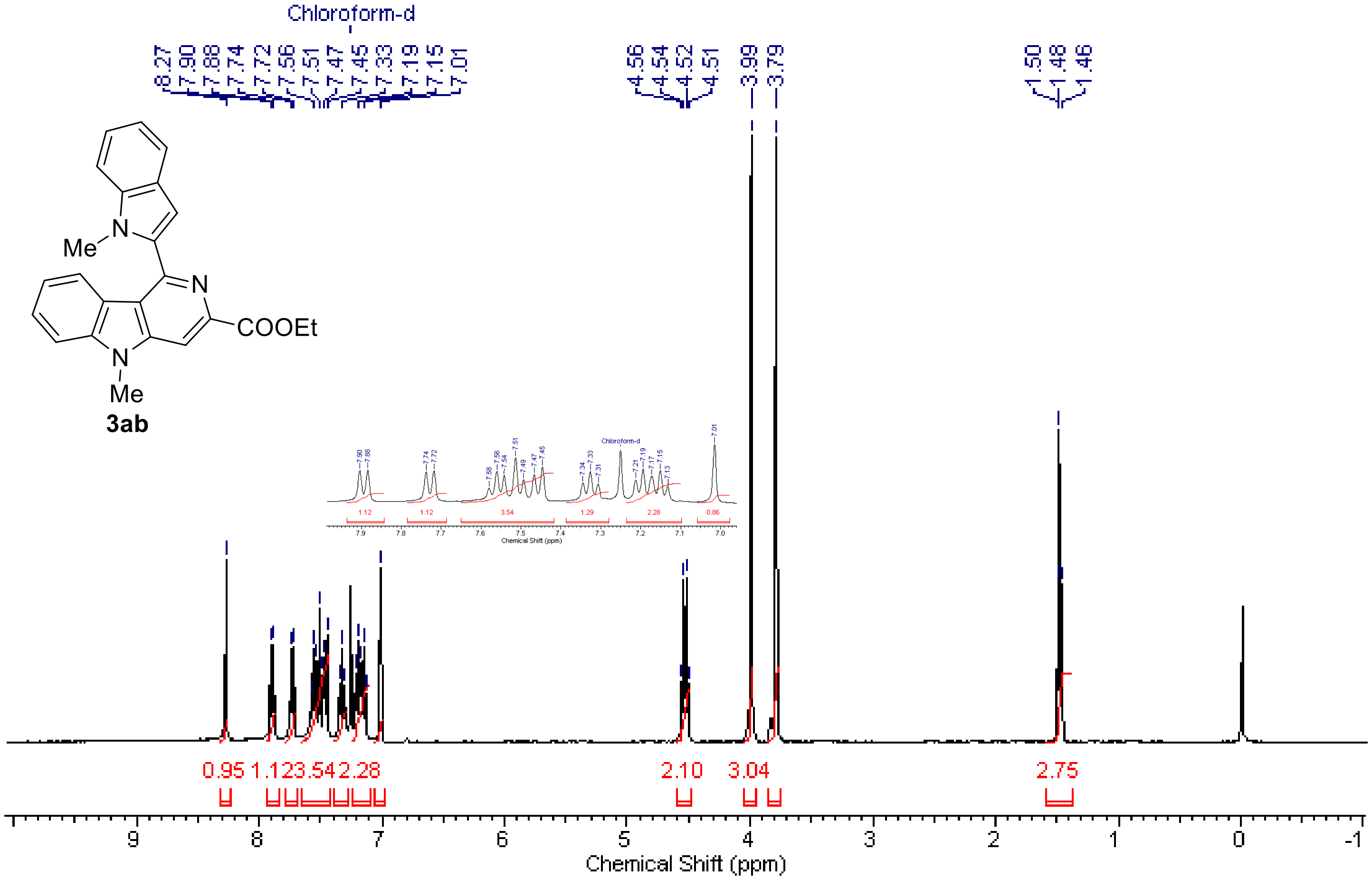

^{13}C NMR spectrum of *ethyl 5-methyl-1-(1-methyl-1H-indol-2-yl)-5H-pyrido[4,3-b]indole-3-carboxylate* (**3ab**)

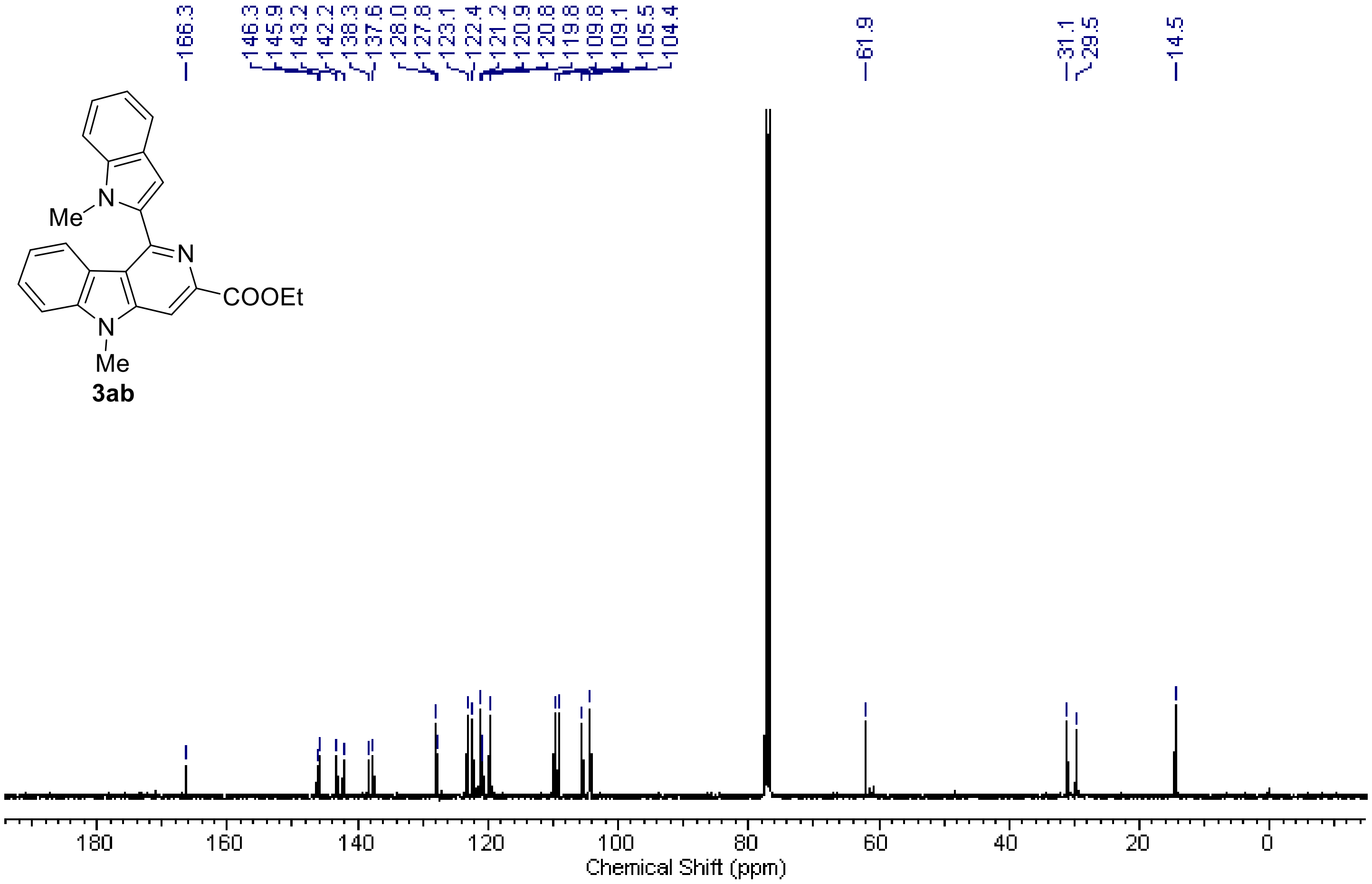

^{1}H NMR spectrum of *tert-butyl 5-methyl-1-(1-methyl-1H-indol-2-yl)-5H-pyrido[4,3-b]indole-3-carboxylate* (**3ac**)

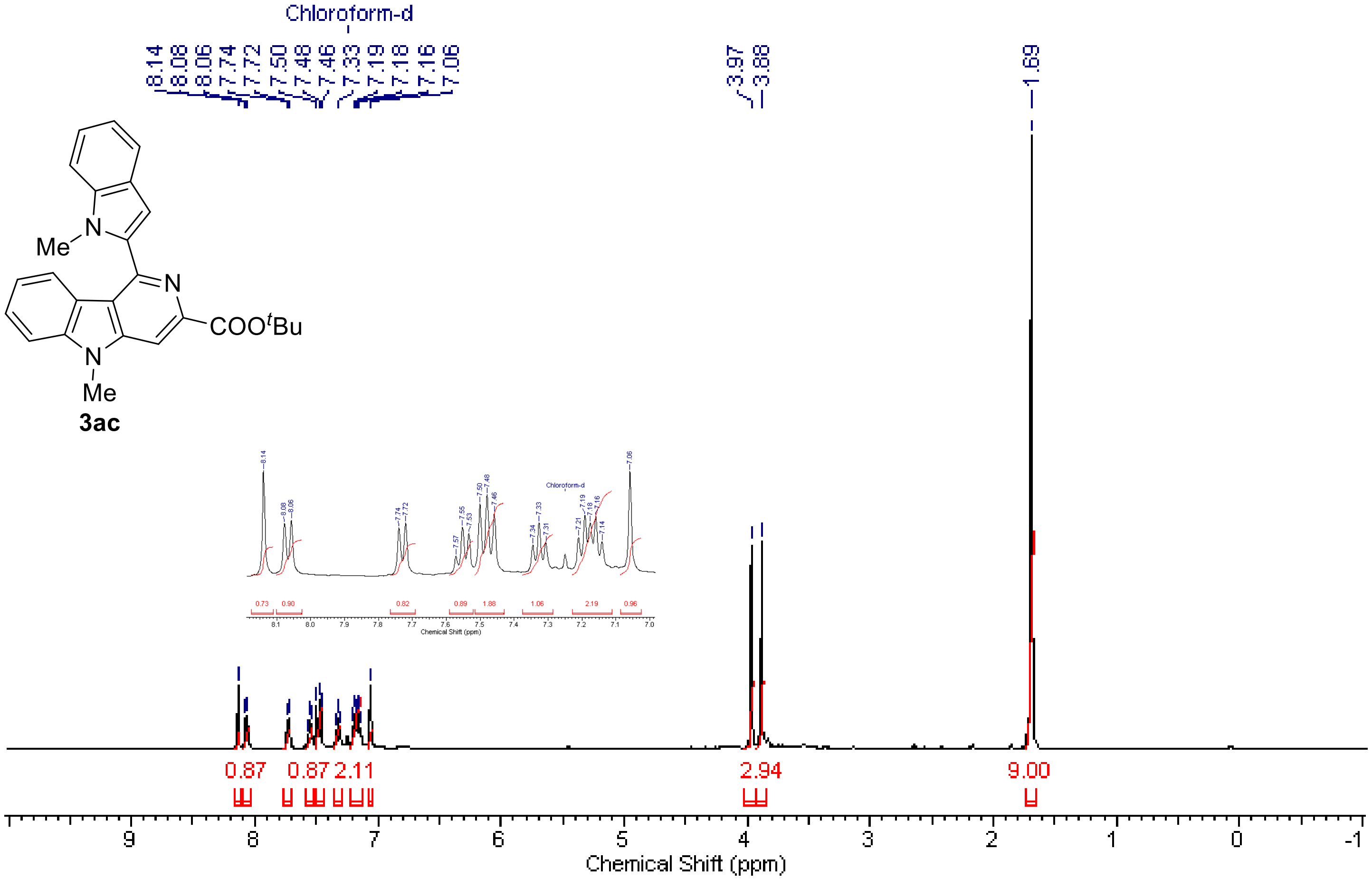

^{13}C NMR spectrum of *tert-butyl 5-methyl-1-(1-methyl-1H-indol-2-yl)-5H-pyrido[4,3-b]indole-3-carboxylate* (**3ac**)

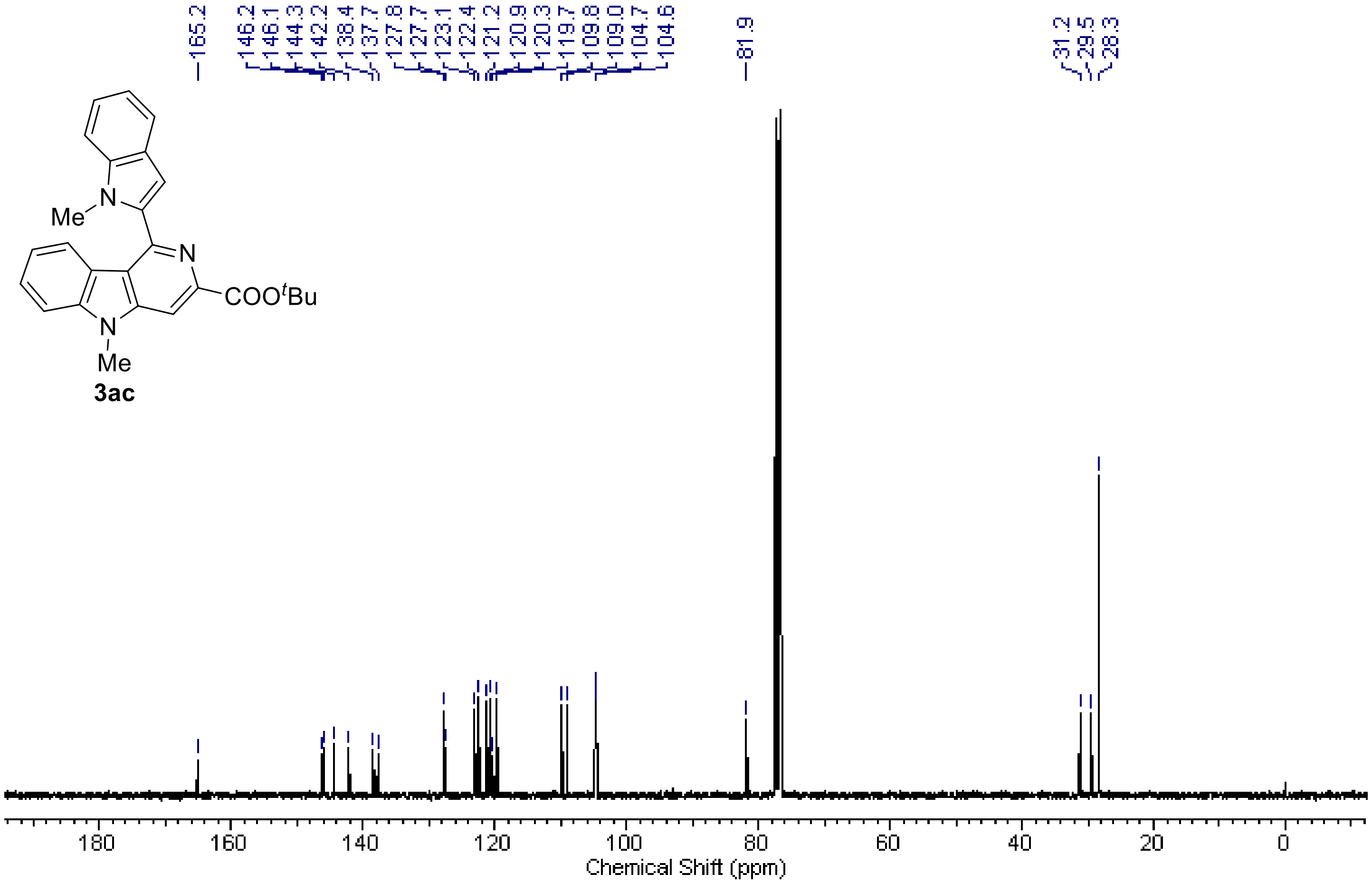

3ac

^{1}H NMR spectrum of *methyl 5-benzyl-1-(1-benzyl-1H-indol-2-yl)-5H-pyrido[4,3-b]indole-3-carboxylate* (**3ba**)

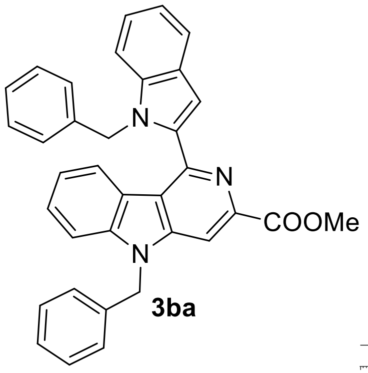

3ba

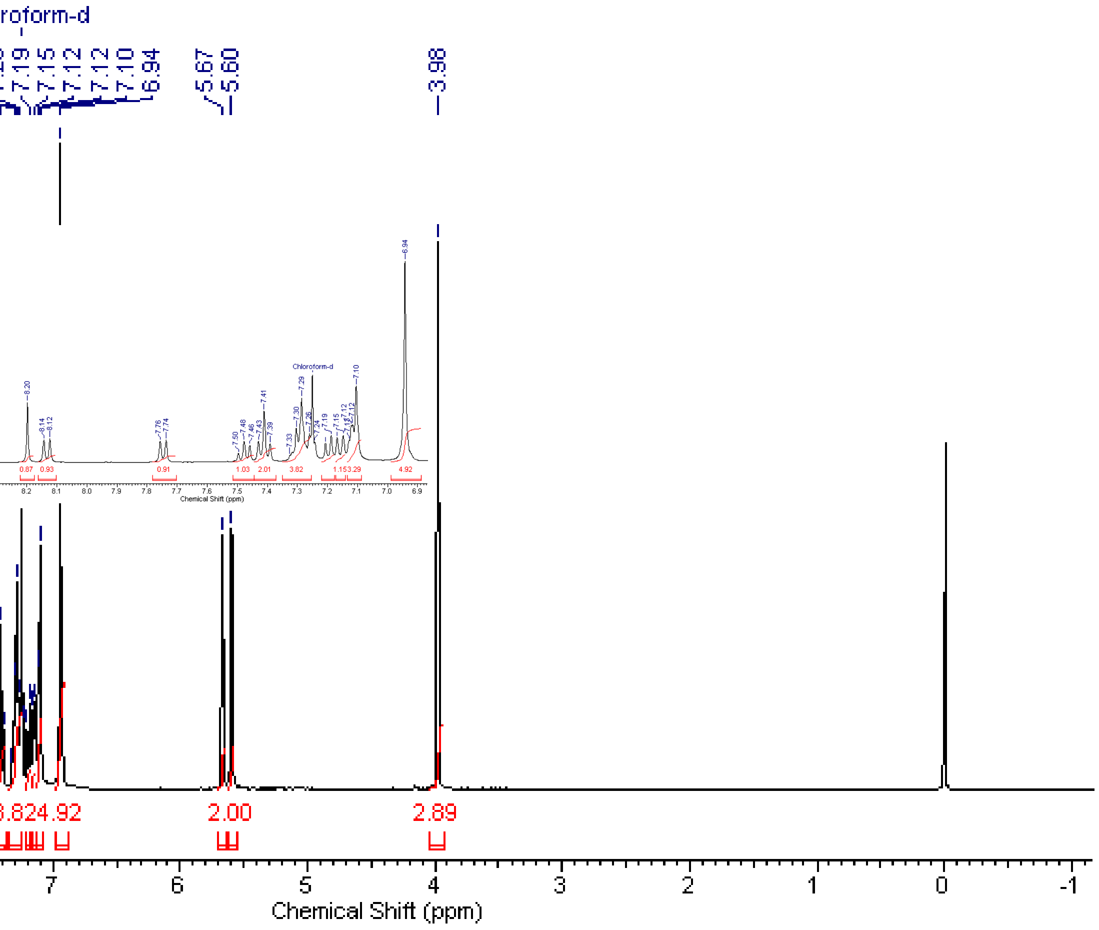

^{13}C NMR spectrum of *methyl 5-benzyl-1-(1-benzyl-1H-indol-2-yl)-5H-pyrido[4,3-b]indole-3-carboxylate* (**3ba**)

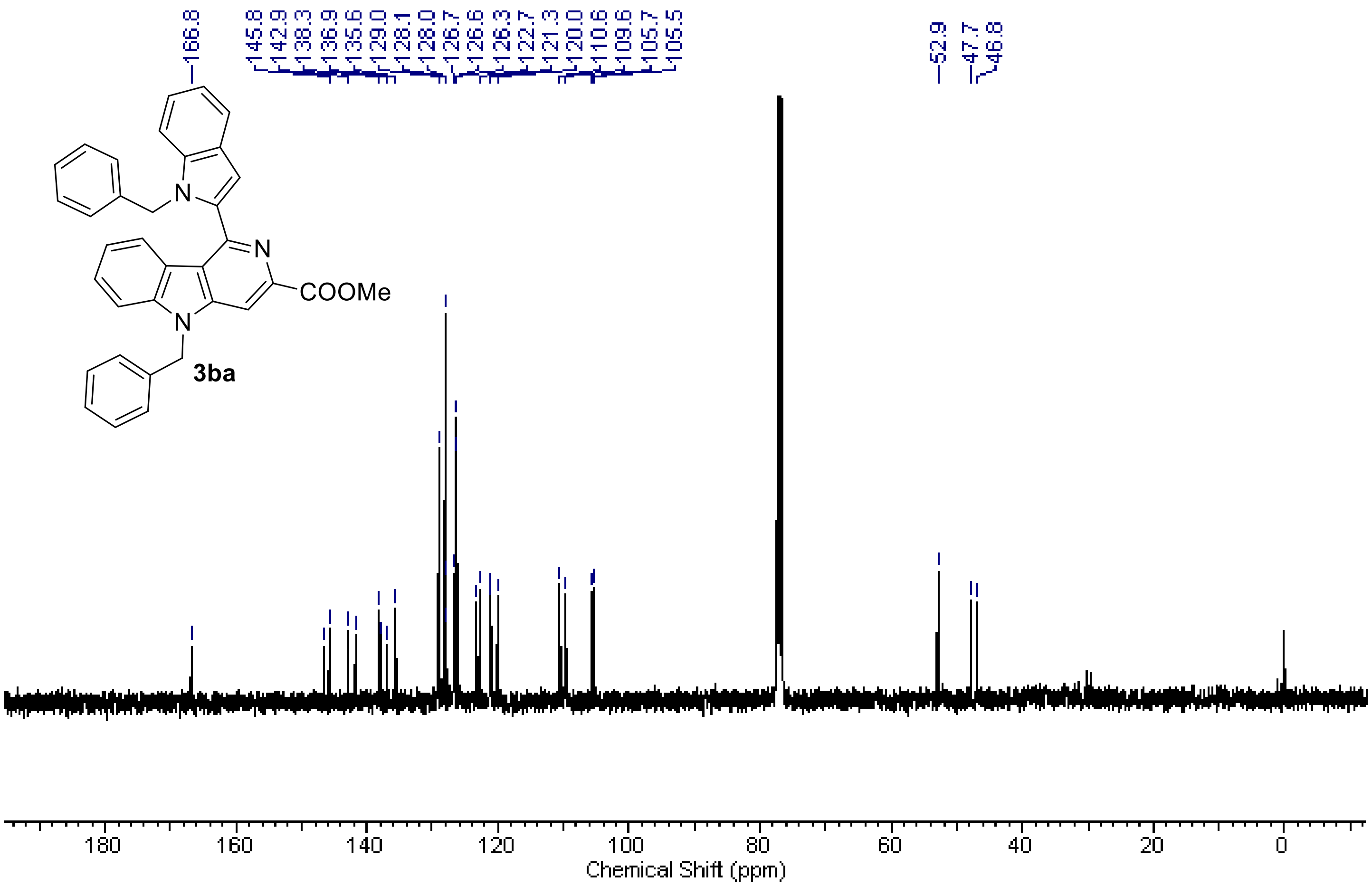

^{1}H NMR spectrum of *ethyl 5-benzyl-1-(1-benzyl-1H-indol-2-yl)-5H-pyrido[4,3-b]indole-3-carboxylate* (**3bb**)

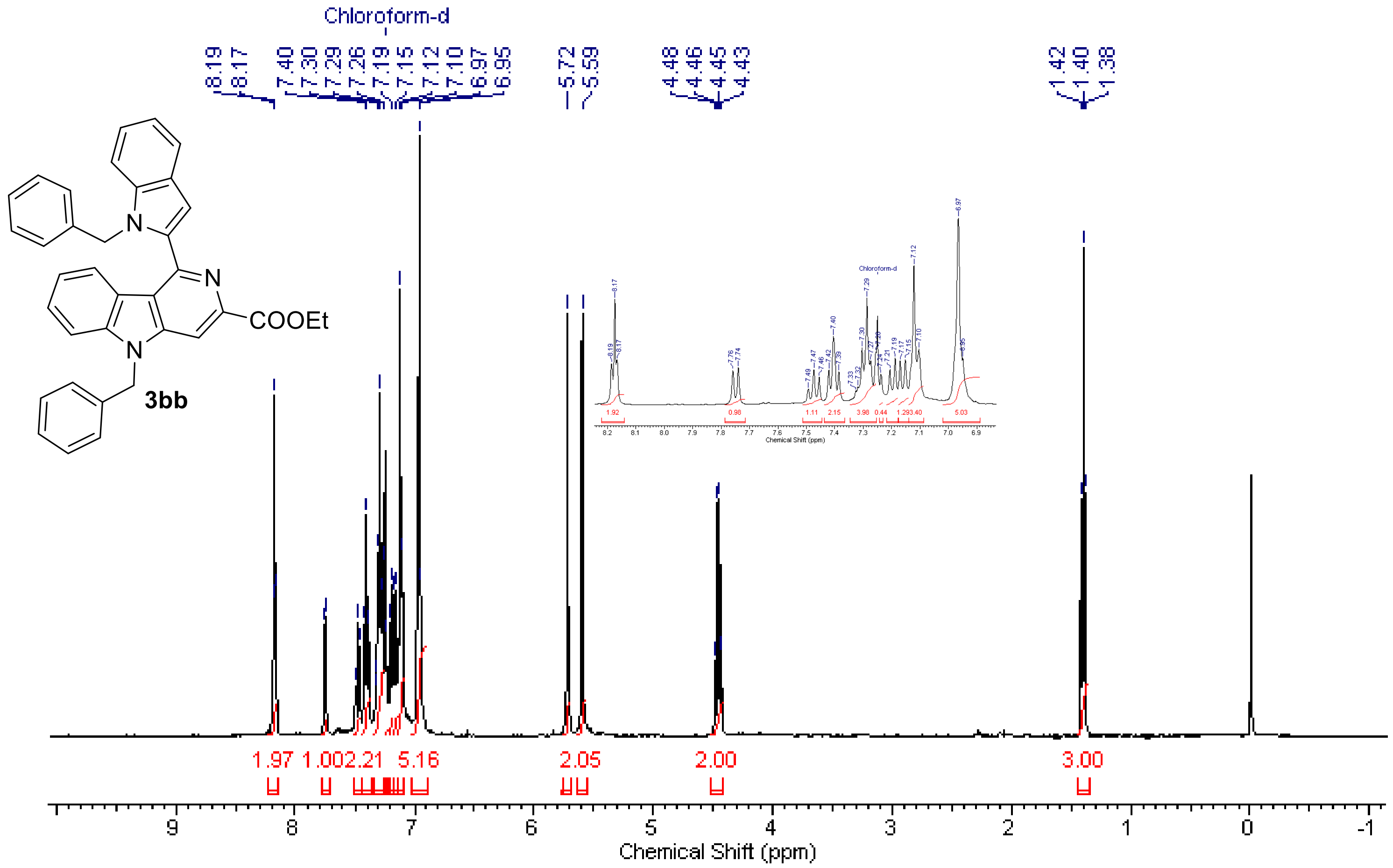

^{13}C NMR spectrum of *ethyl 5-benzyl-1-(1-benzyl-1H-indol-2-yl)-5H-pyrido[4,3-b]indole-3-carboxylate* (**3bb**)

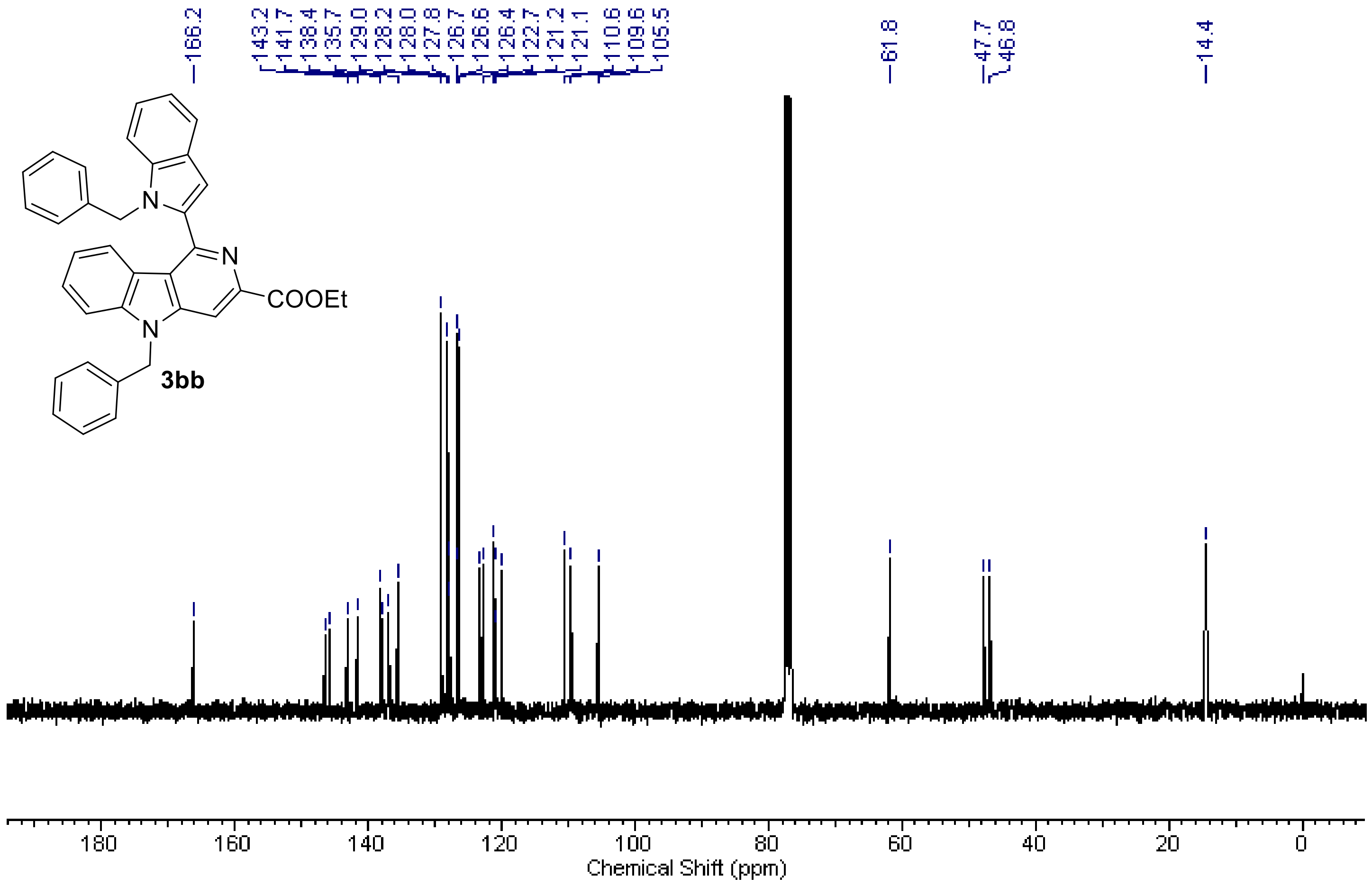

^{1}H NMR spectrum of *tert-butyl 5-benzyl-1-(1-benzyl-1H-indol-2-yl)-5H-pyrido[4,3-b]indole-3-carboxylate* (**3bc**)

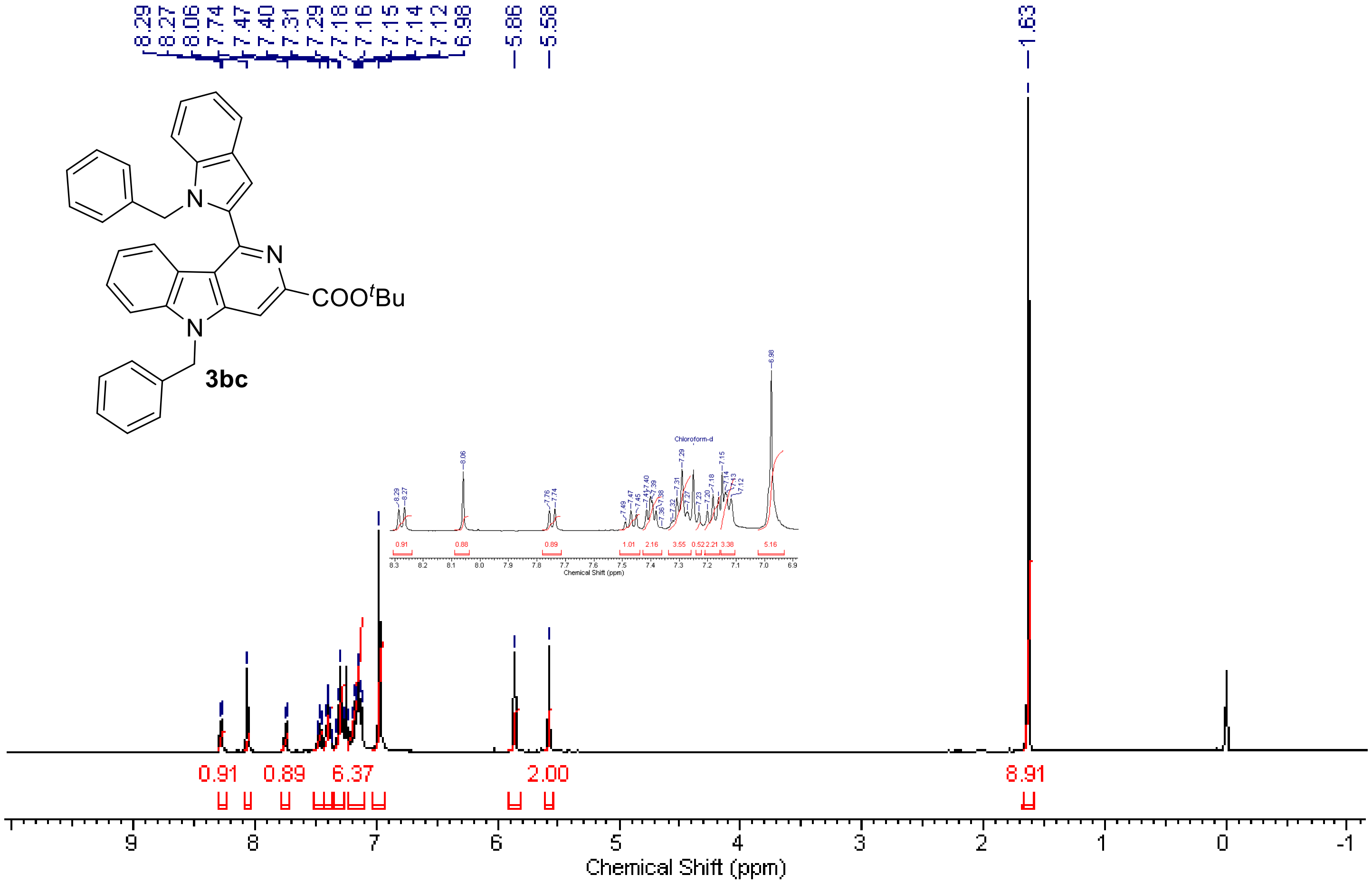

^{13}C NMR spectrum of *tert-butyl 5-benzyl-1-(1-benzyl-1H-indol-2-yl)-5H-pyrido[4,3-b]indole-3-carboxylate* (**3bc**)

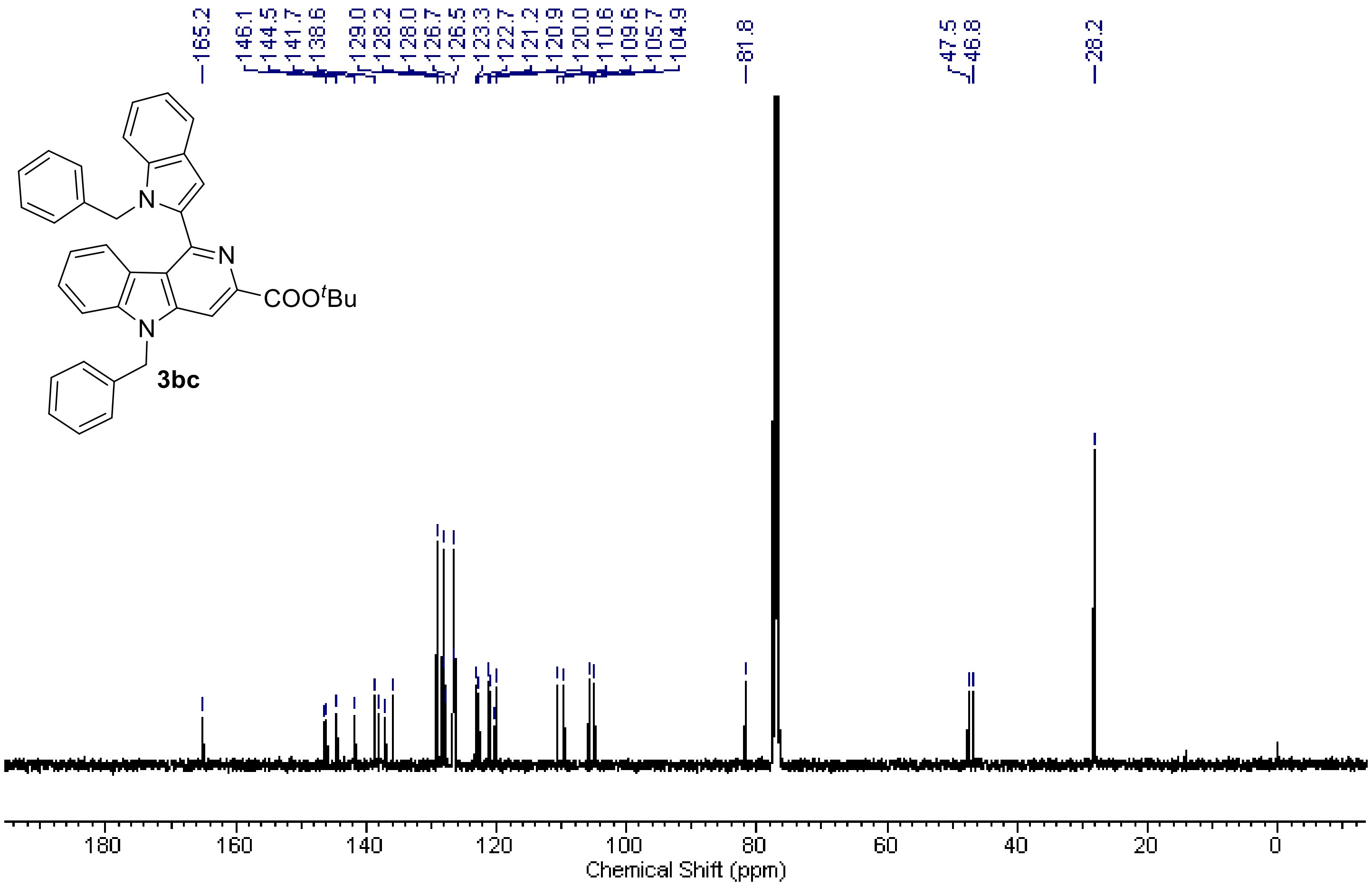

^{1}H NMR spectrum of *methyl 5-(4-methoxybenzyl)-1-(1-(4-methoxybenzyl)-1H-indol-2-yl)-5H-pyrido[4,3-b]indole-3-carboxylate* (**3ca**)

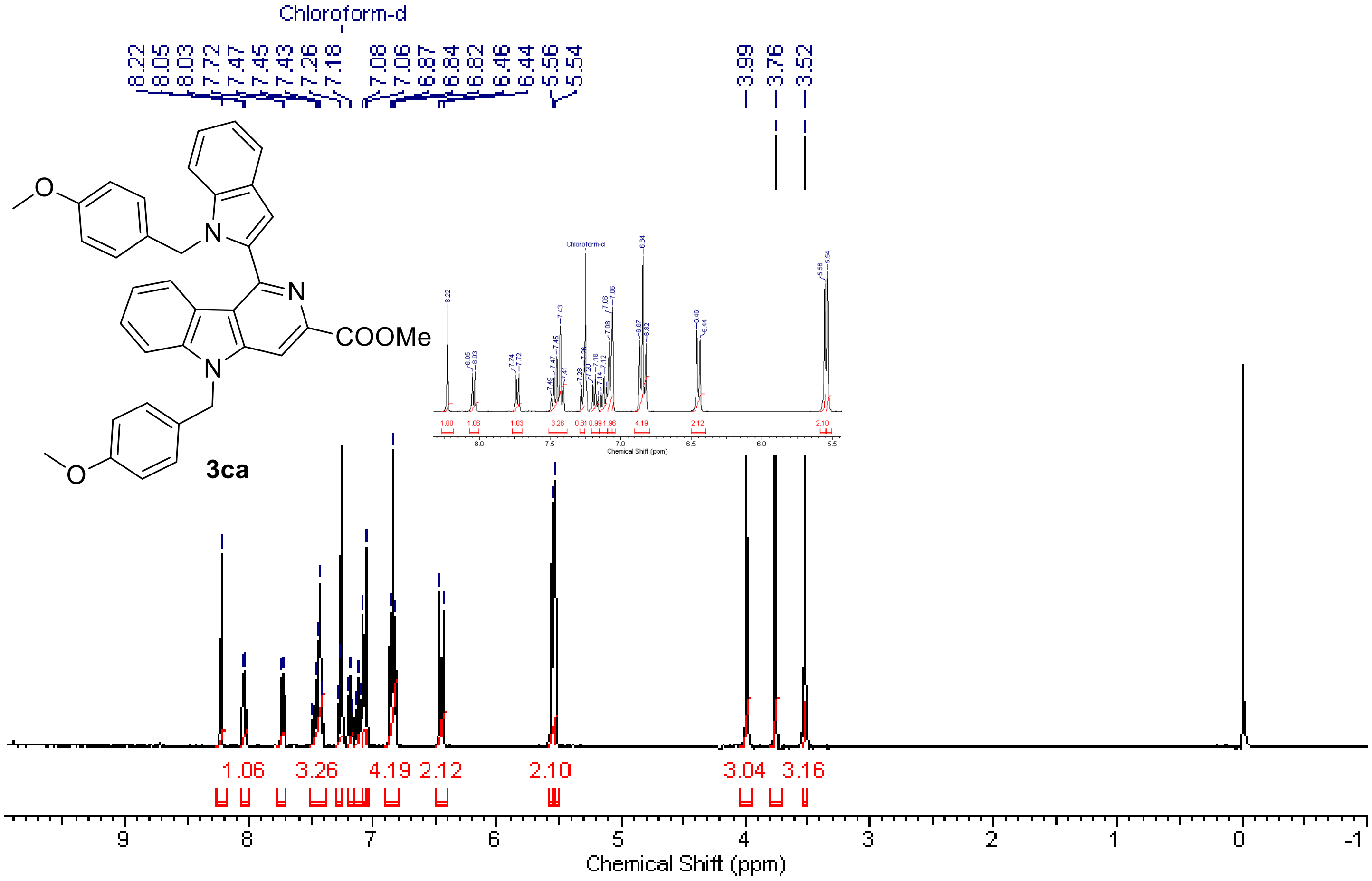

^{13}C NMR spectrum of *methyl 5-(4-methoxybenzyl)-1-(1-(4-methoxybenzyl)-1H-indol-2-yl)-5H-pyrido[4,3-b]indole-3-carboxylate* (**3ca**)

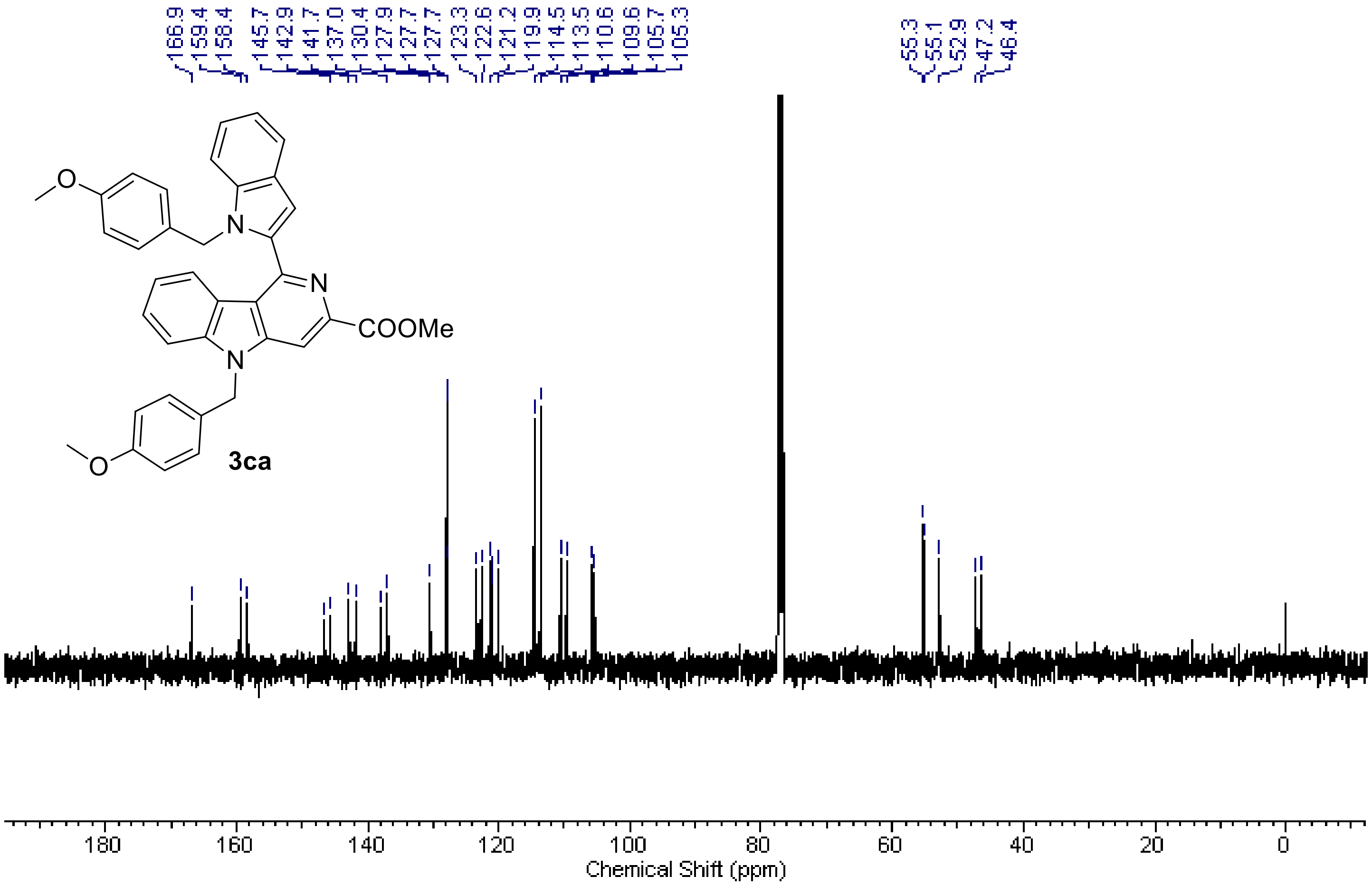

^{1}H NMR spectrum of *methyl 5-butyl-1-(1-butyl-1H-indol-2-yl)-5H-pyrido[4,3-b]indole-3-carboxylate* (**3da**)

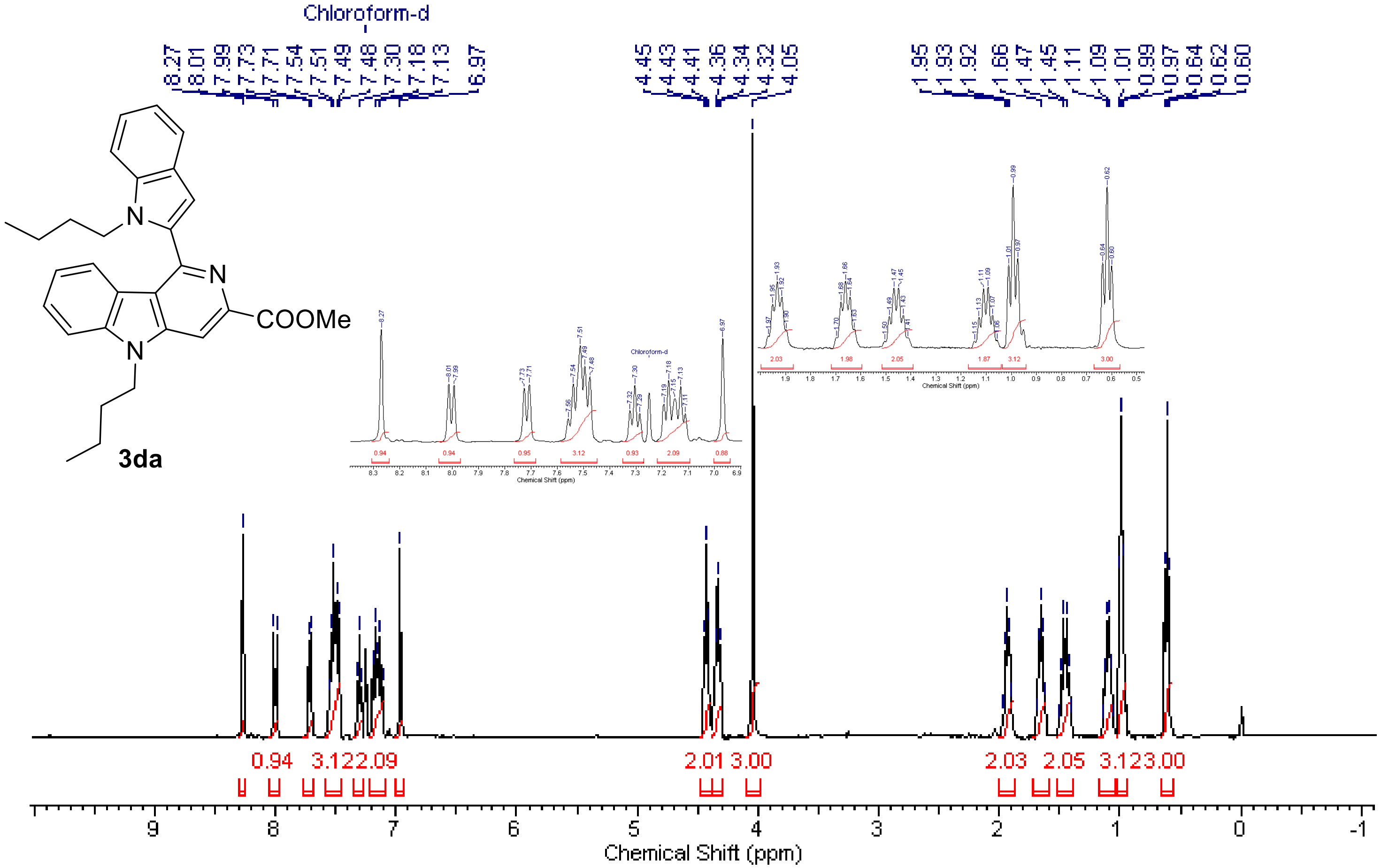

^{13}C NMR spectrum of *methyl 5-butyl-1-(1-butyl-1H-indol-2-yl)-5H-pyrido[4,3-b]indole-3-carboxylate* (**3da**)

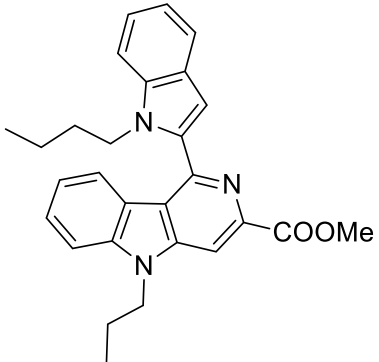

3da

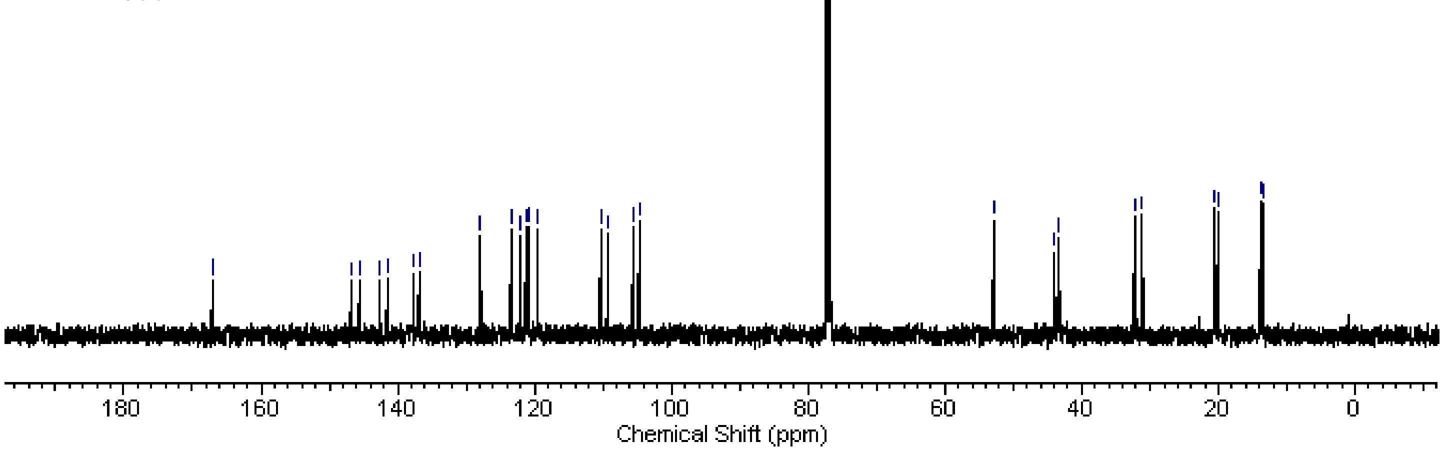

^{1}H NMR spectrum of *methyl 8-methoxy-1-(5-methoxy-1-methyl-1H-indol-2-yl)-5-methyl-5H-pyrido[4,3-b]indole-3-carboxylate* (**3ea**)

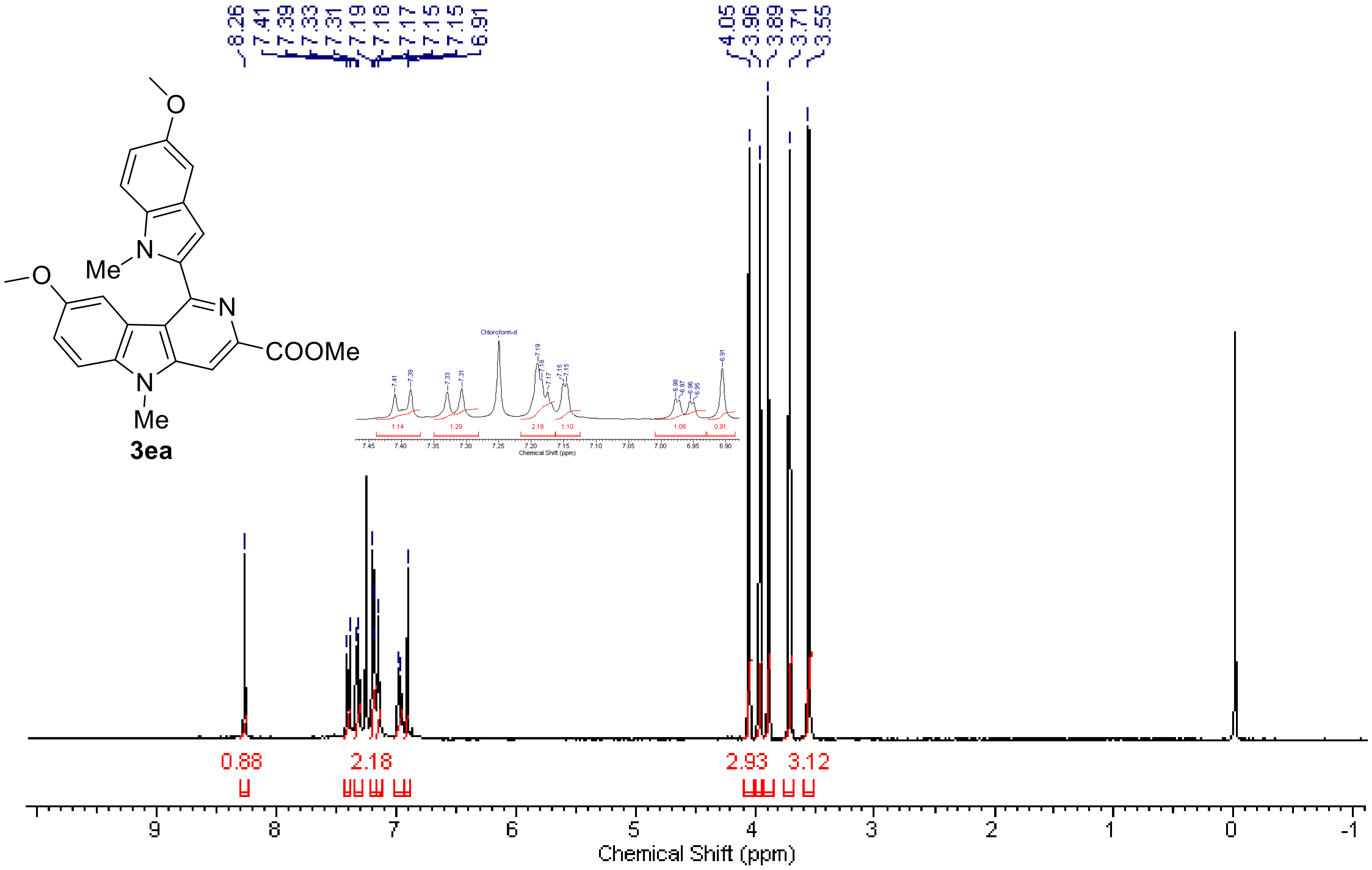

^{13}C NMR spectrum of *methyl 8-methoxy-1-(5-methoxy-1-methyl-1H-indol-2-yl)-5-methyl-5H-pyrido[4,3-b]indole-3-carboxylate* (**3ea**)

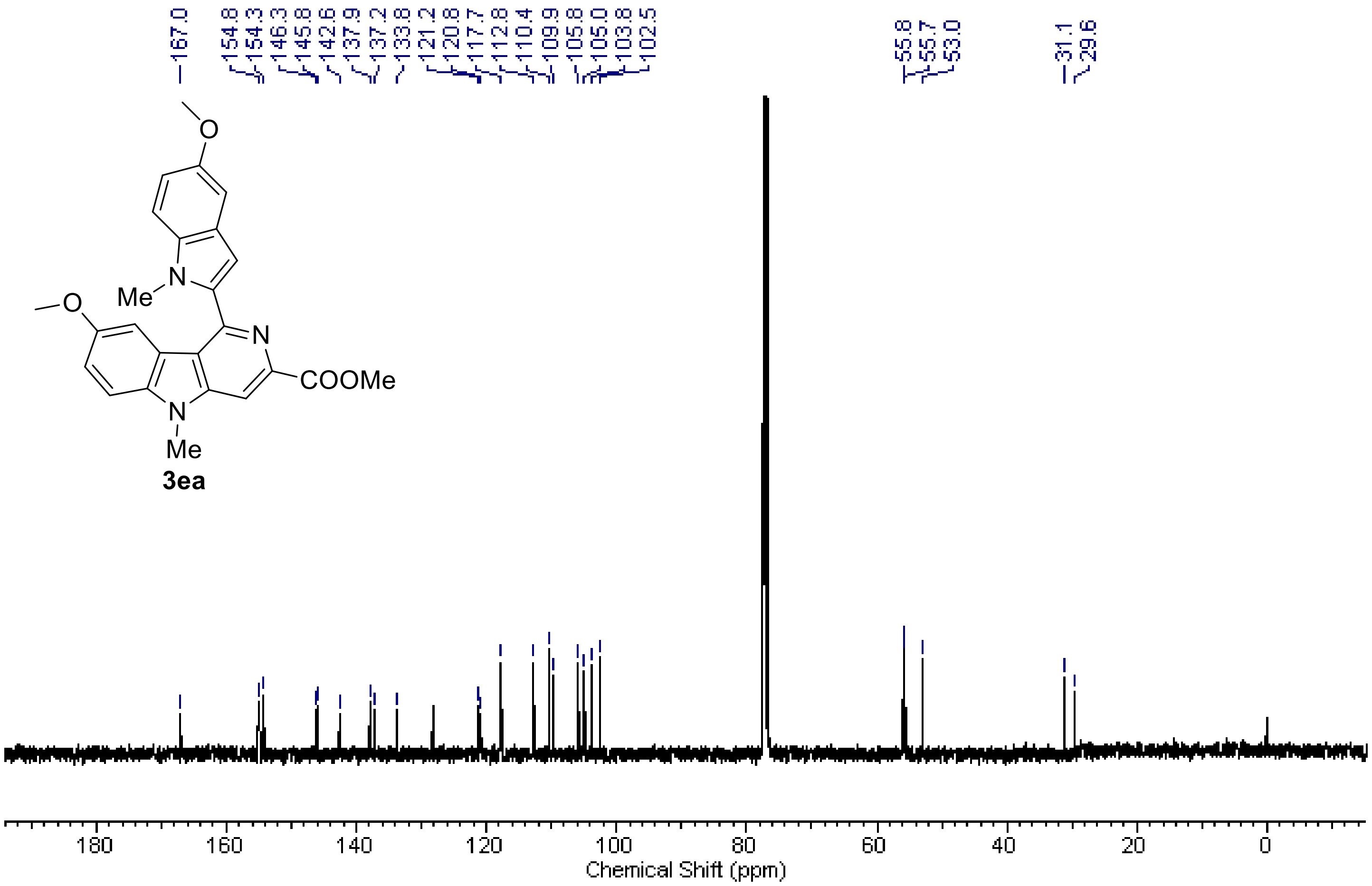

^{1}H NMR spectrum of *methyl 5-tosyl-1-(1-tosyl-1H-indol-2-yl)-2,5-dihydro-1H-pyrido[4,3-b]indole-3-carboxylate* (**3ga**)

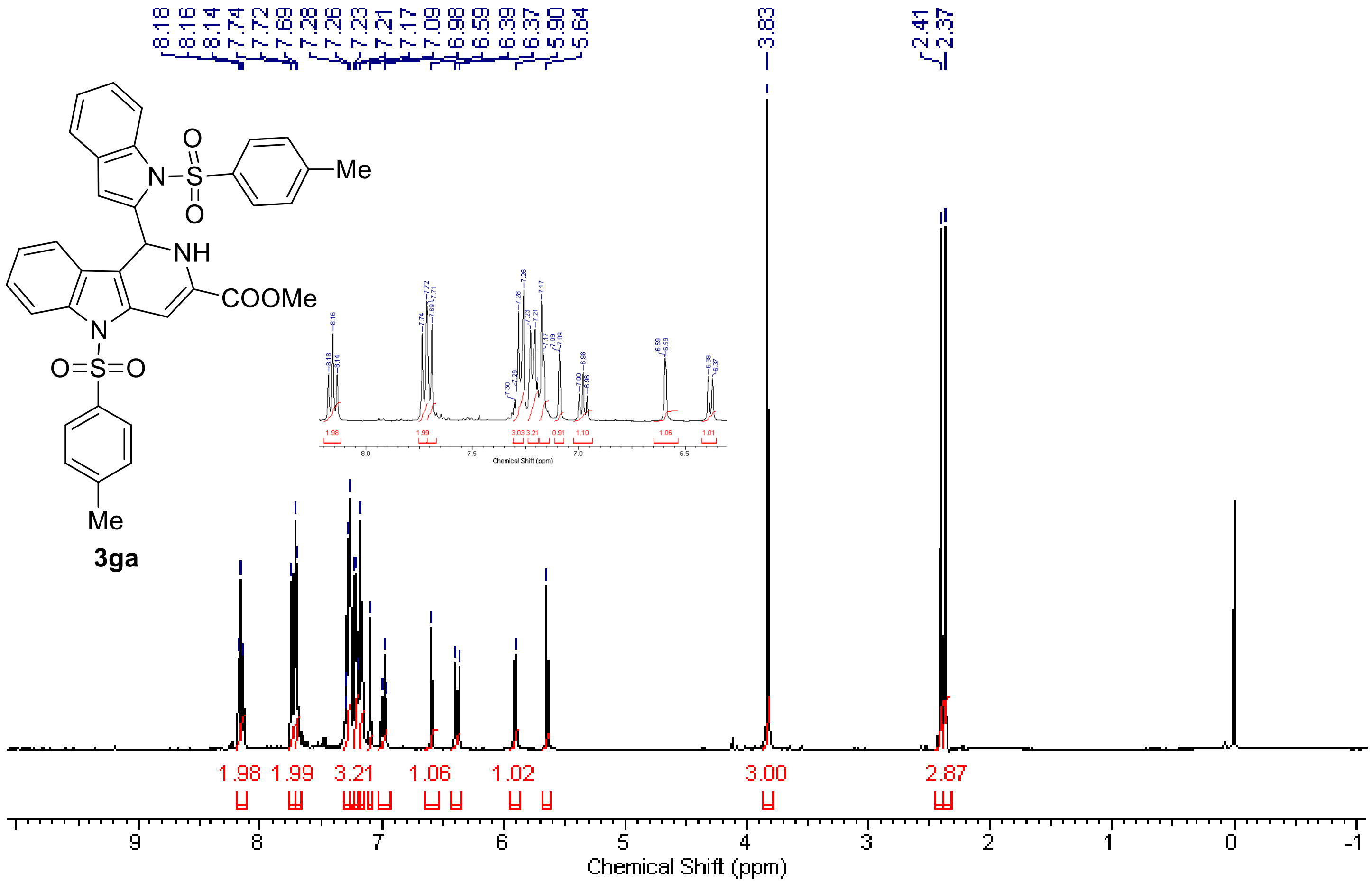

^{13}C NMR spectrum of *methyl 5-tosyl-1-(1-tosyl-1H-indol-2-yl)-2,5-dihydro-1H-pyrido[4,3-b]indole-3-carboxylate* (**3ga**)

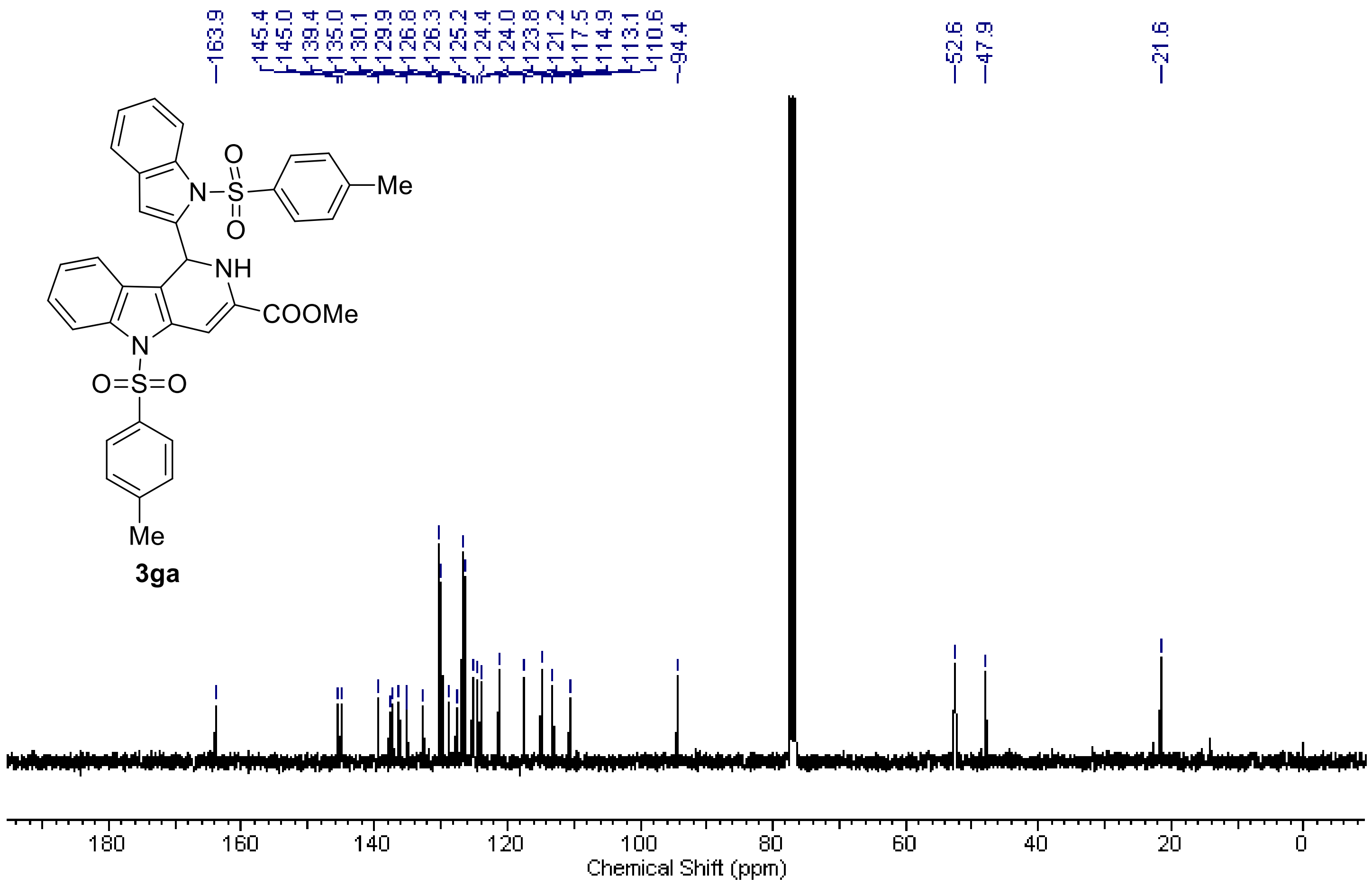

^{1}H NMR spectrum of *1-methyl-1H-indole* (**12a**)

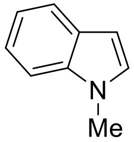

12a

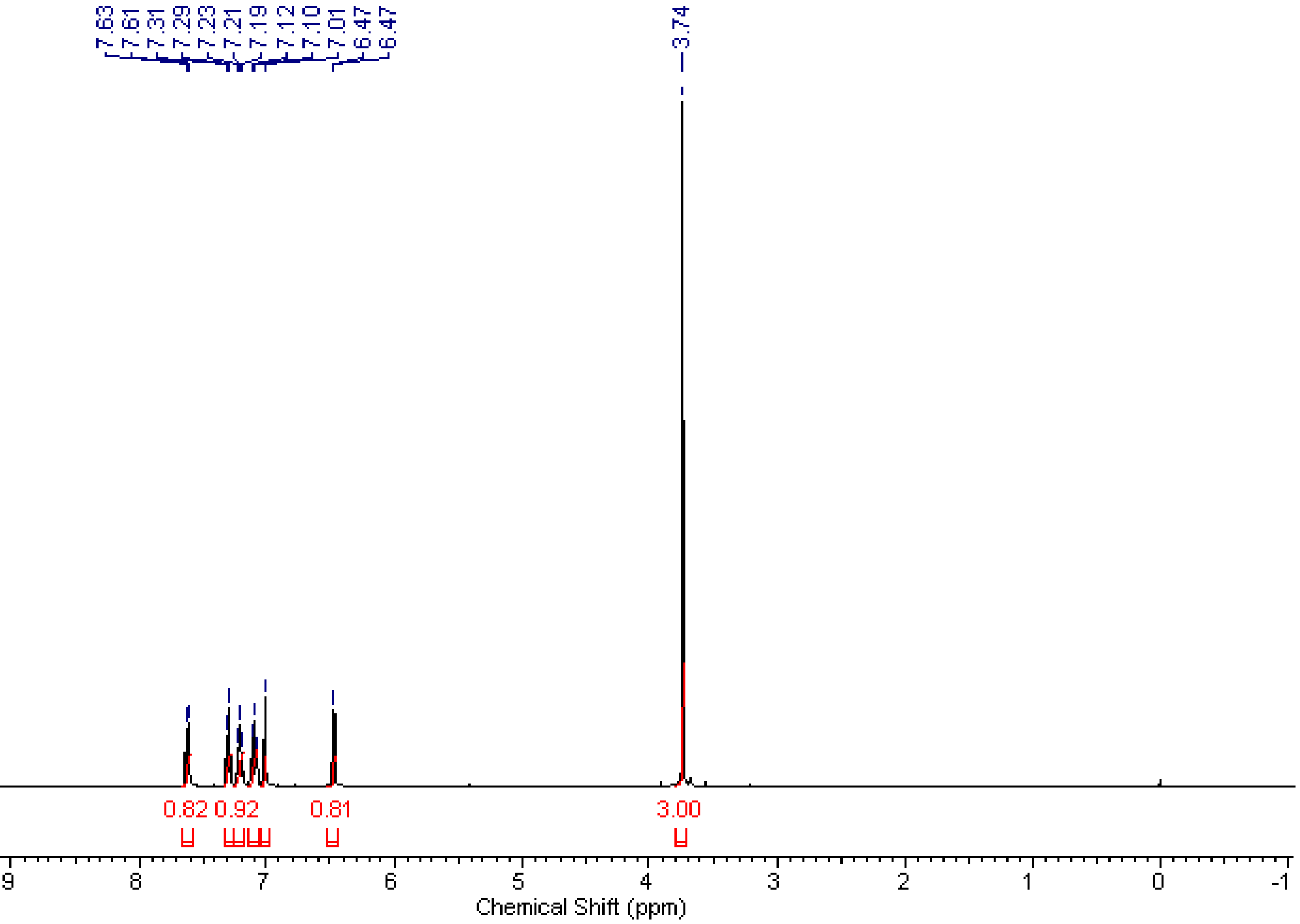

^{13}C NMR spectrum of *1-methyl-1H-indole* (**12a**)

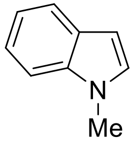

12a

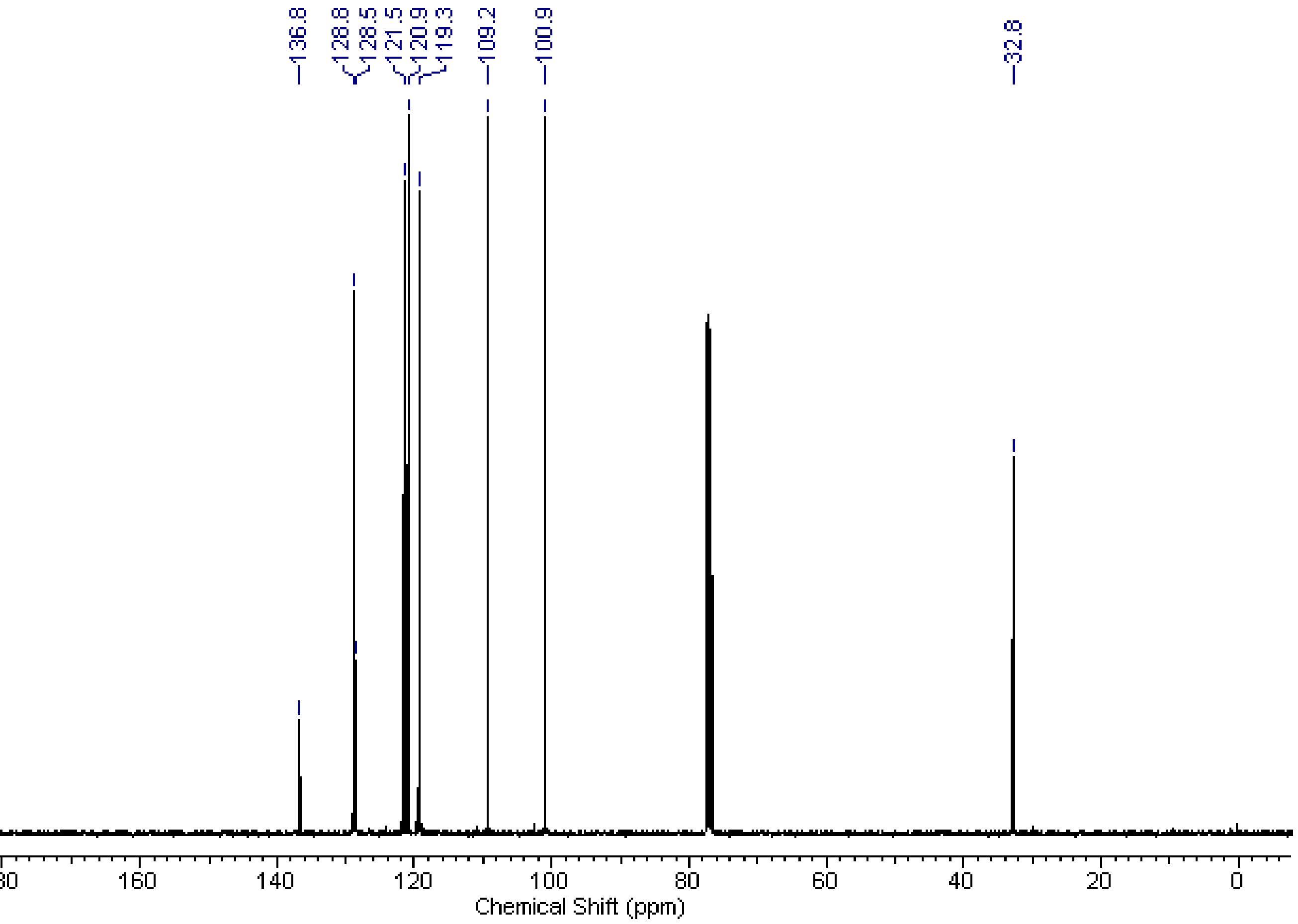

^{1}H NMR spectrum of *1-benzyl-1H-indole* (**12b**)

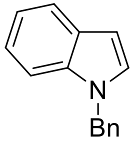

12b

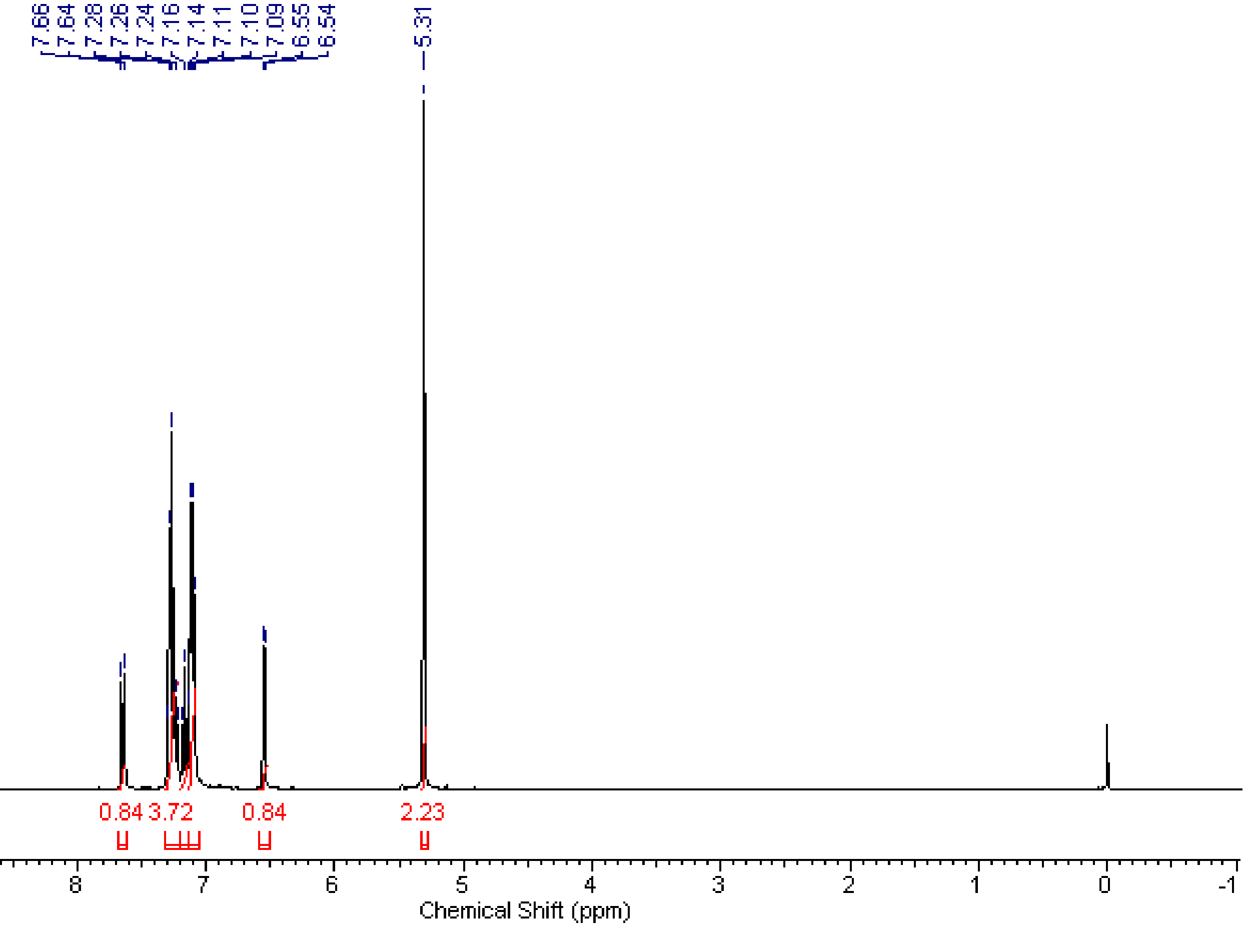

^{13}C NMR spectrum of *1-benzyl-1H-indole* (**12b**)

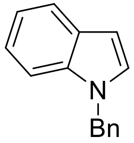

12b

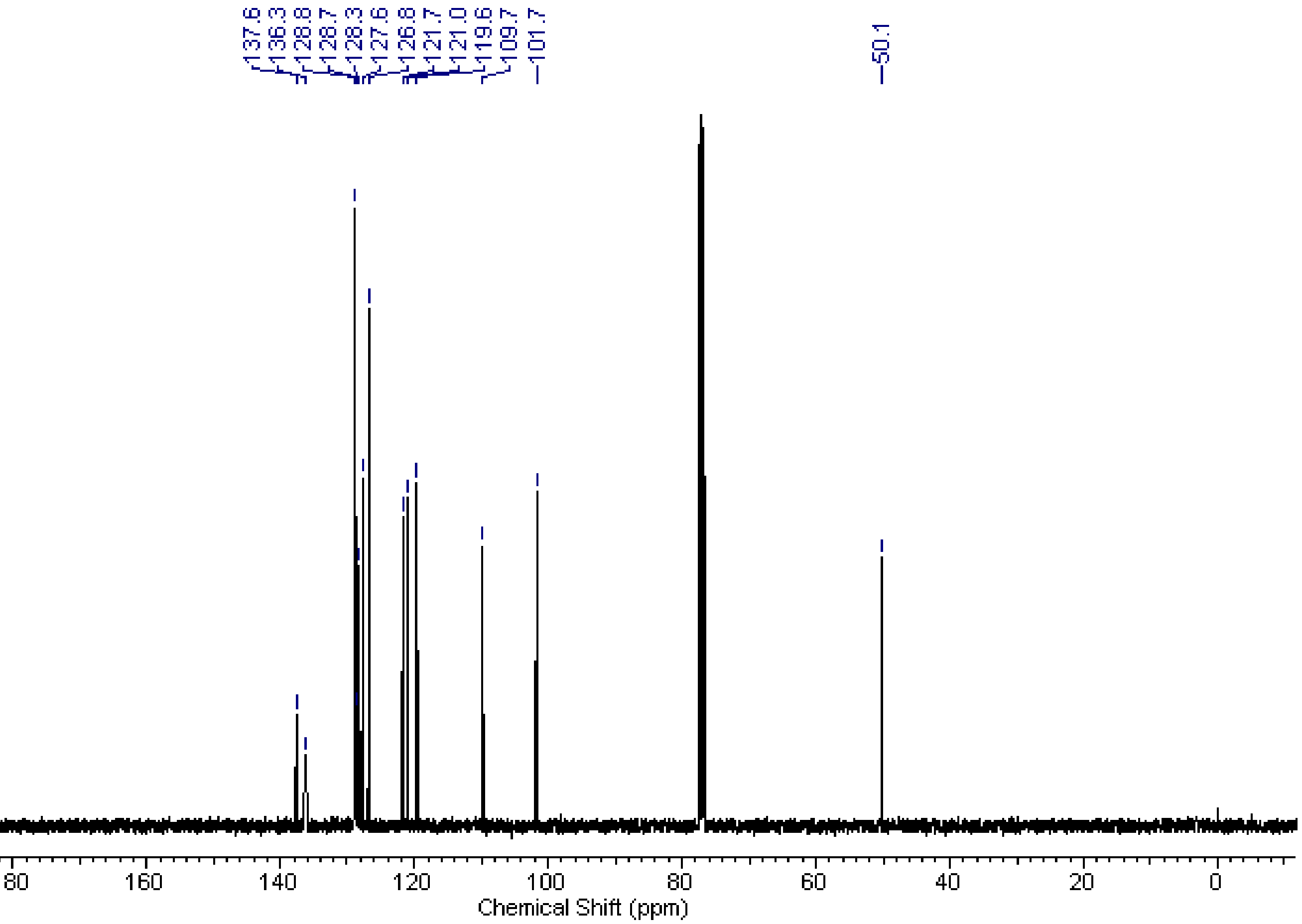

^{1}H NMR spectrum of 5-methoxy-1-methyl-1H-indole (**12e**)

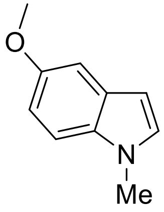

12e

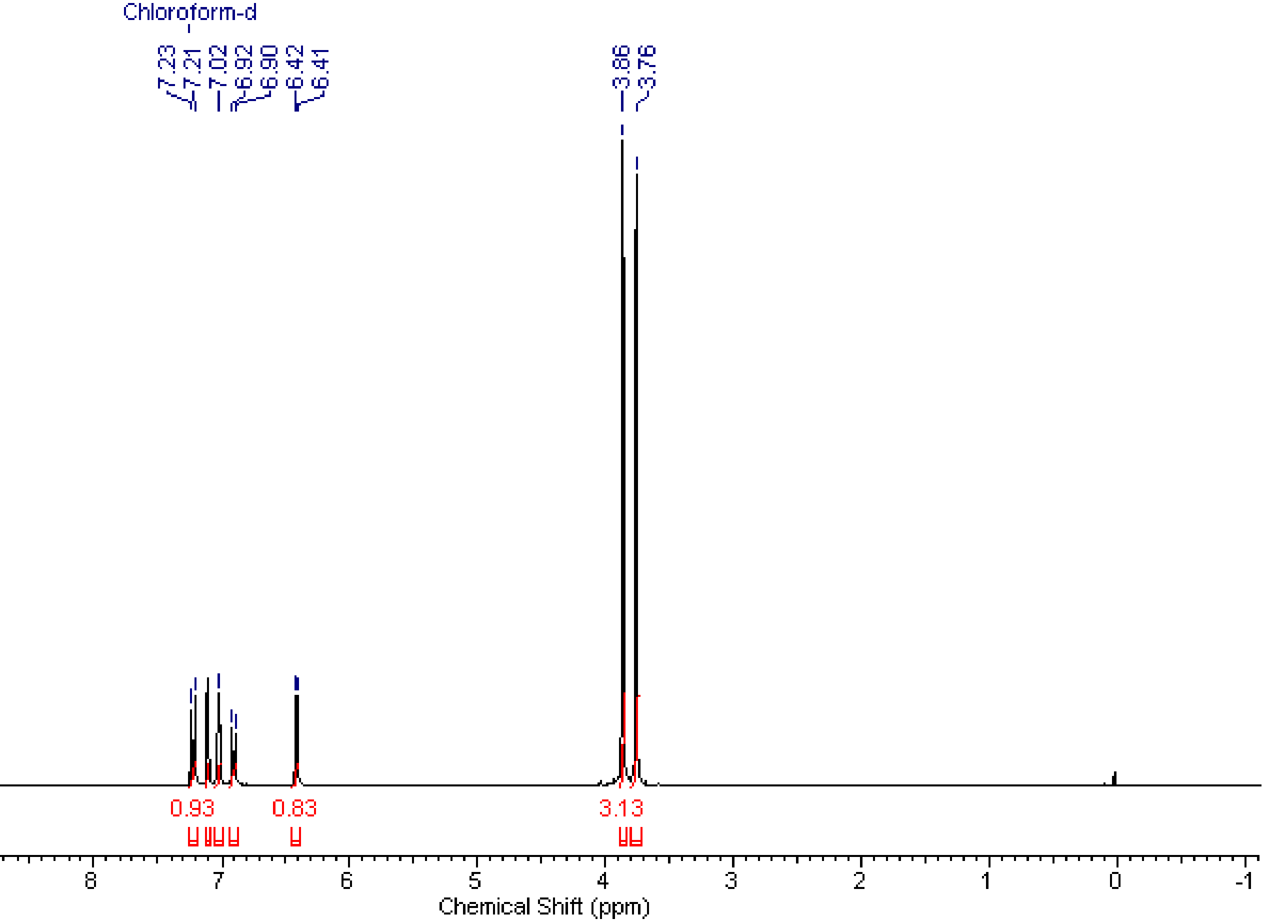

^{13}C NMR spectrum of 5-methoxy-1-methyl-1H-indole (**12e**)

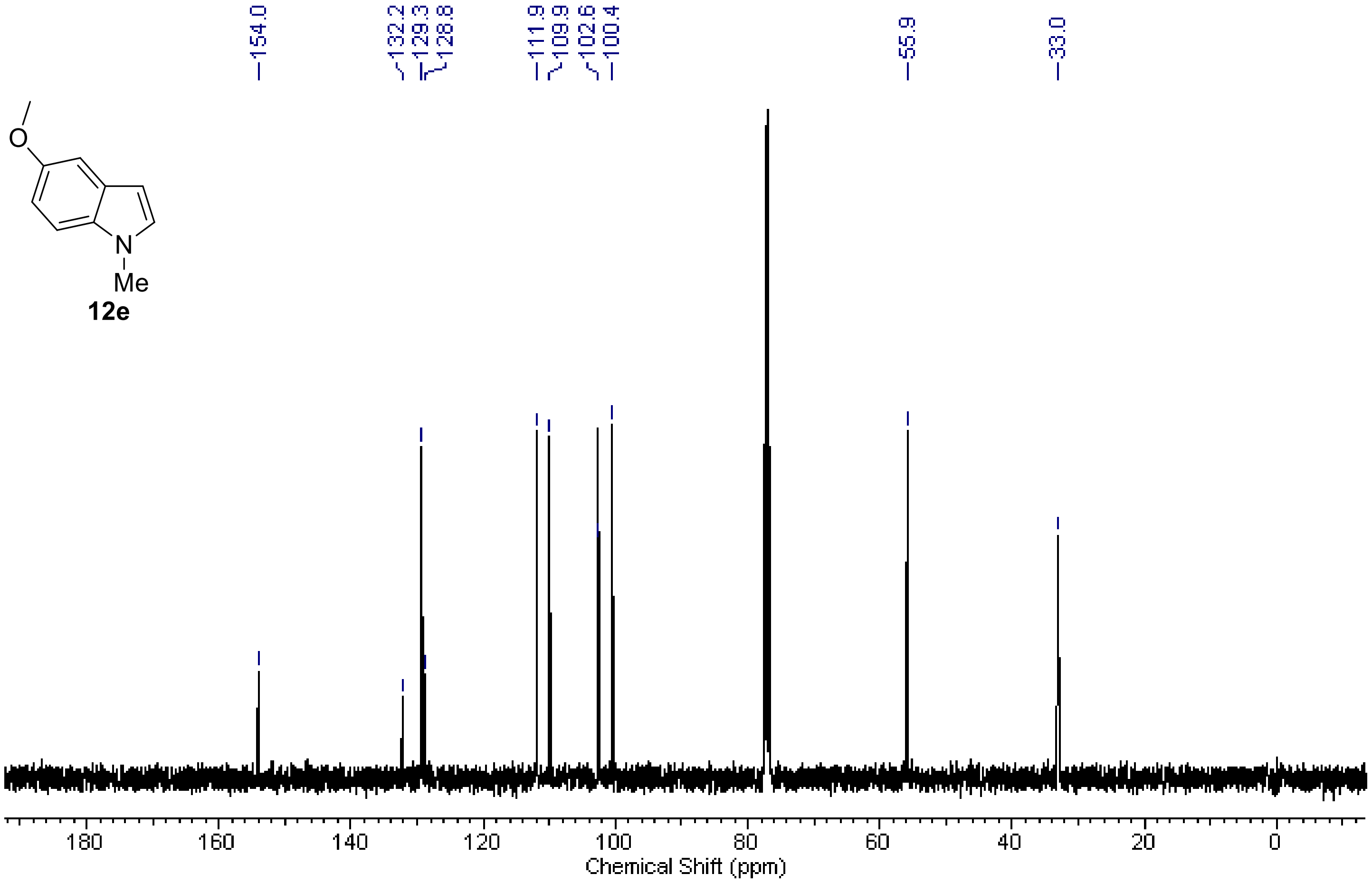

12e

^{1}H NMR spectrum of *1-methyl-5-phenyl-1H-indole* (**12f**)

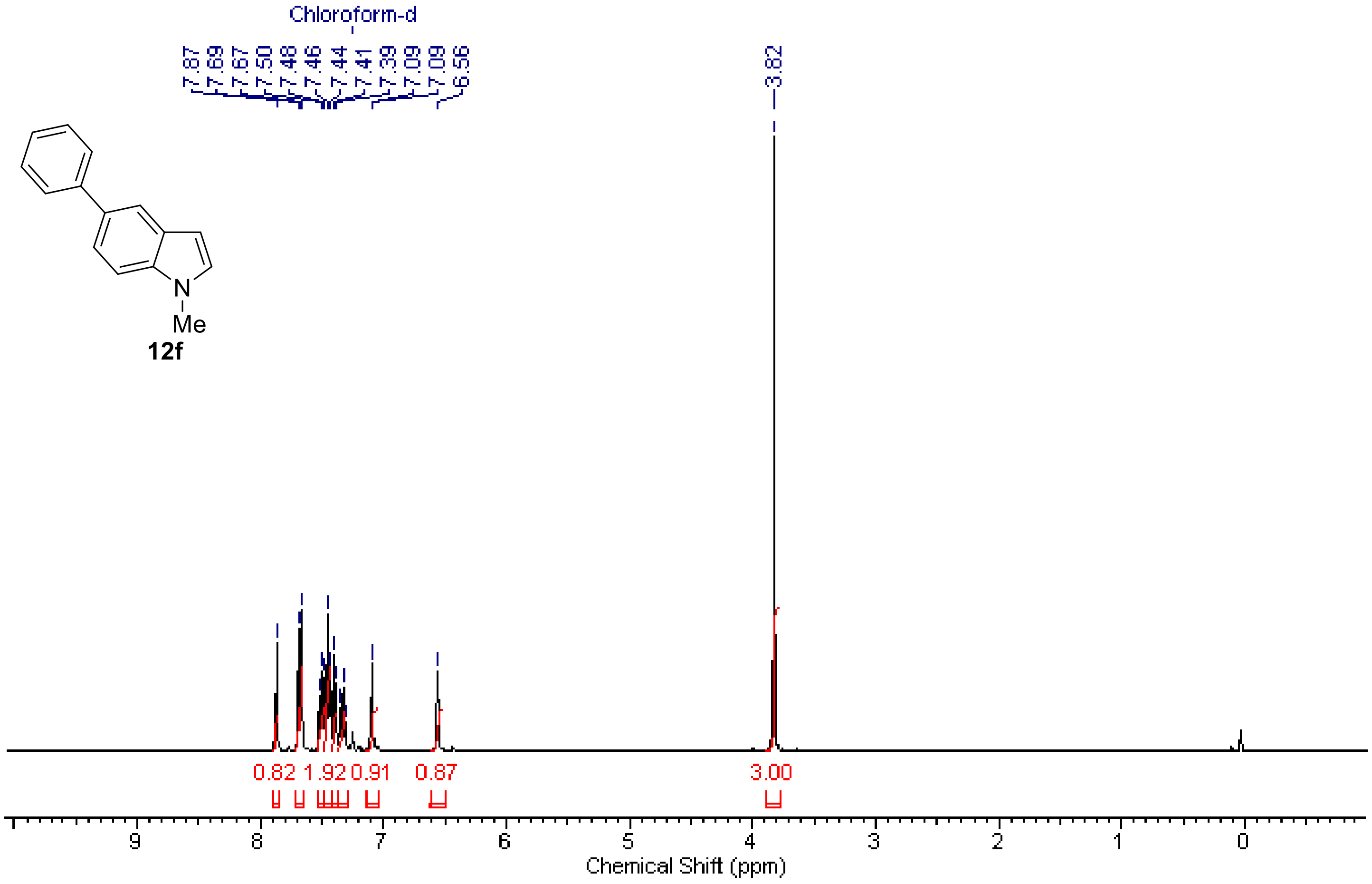

^{13}C NMR spectrum of *1-methyl-5-phenyl-1H-indole* (**12f**)

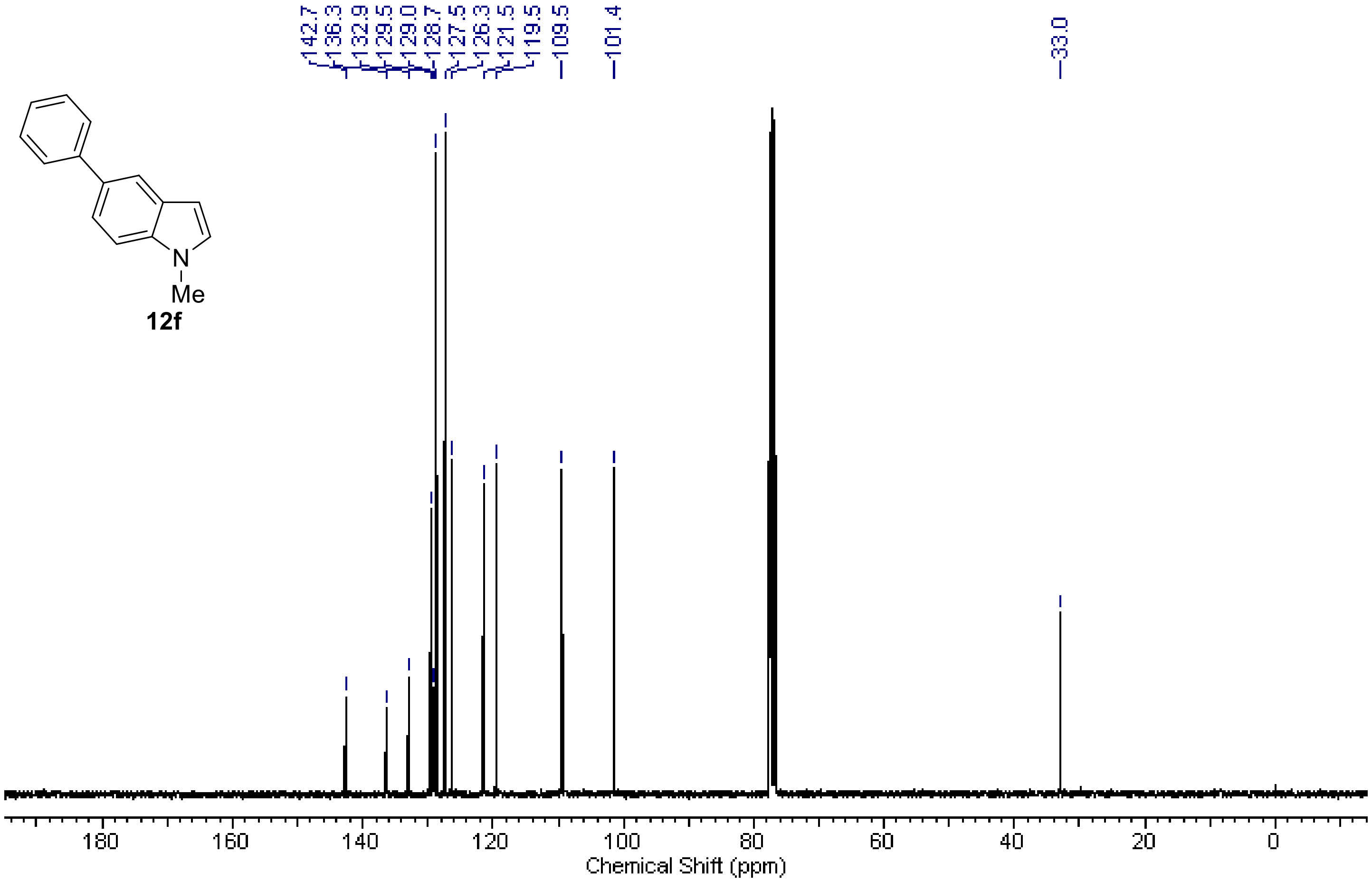

^{1}H NMR spectrum of *5-bromo-1-methyl-1H-indole* (**12i**)

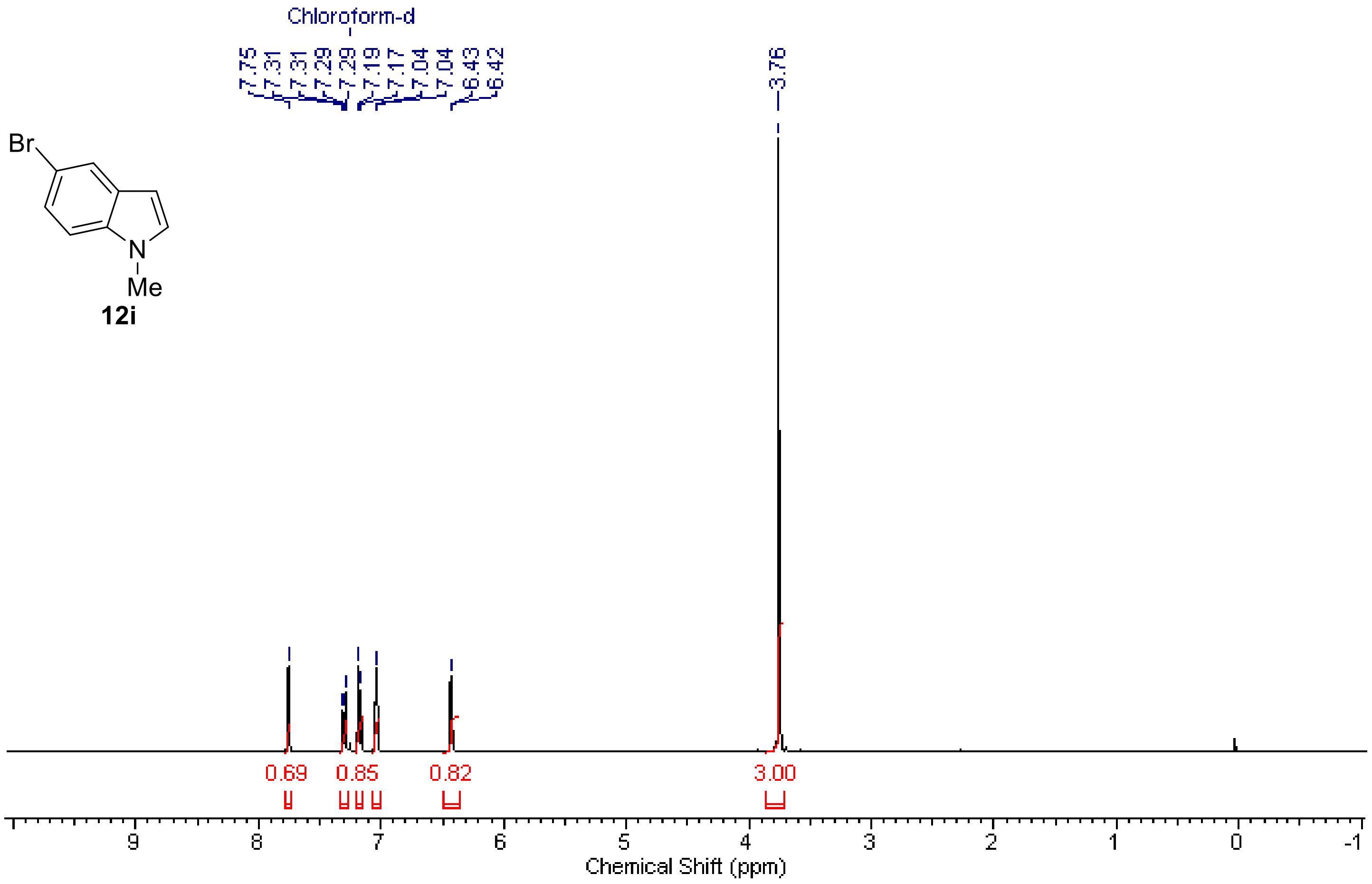

^{13}C NMR spectrum of *5-bromo-1-methyl-1H-indole* (**12i**)

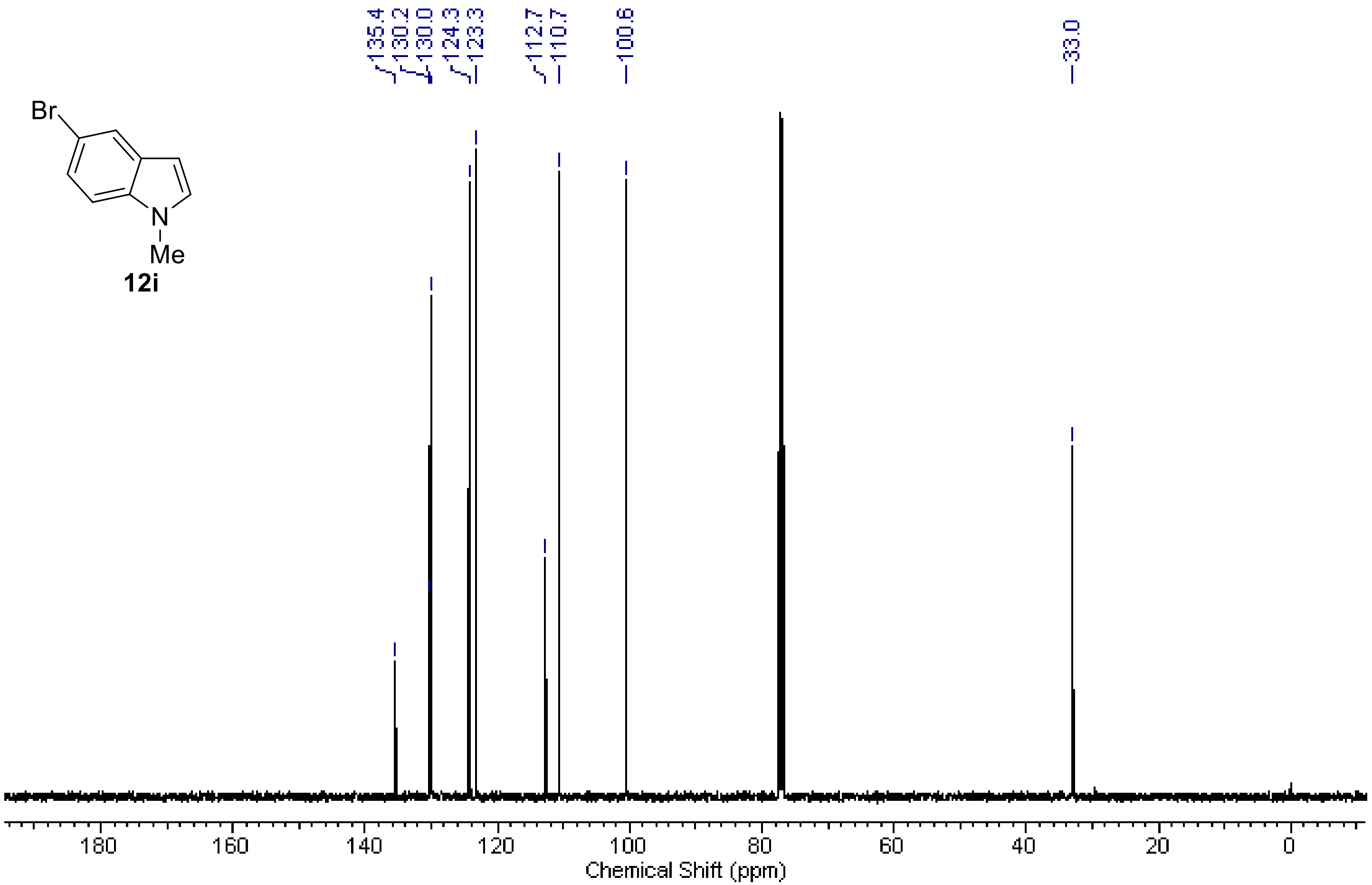

^{1}H NMR spectrum of *ethyl 1-butyl-1H-indole-2-carboxylate* (**14d**)

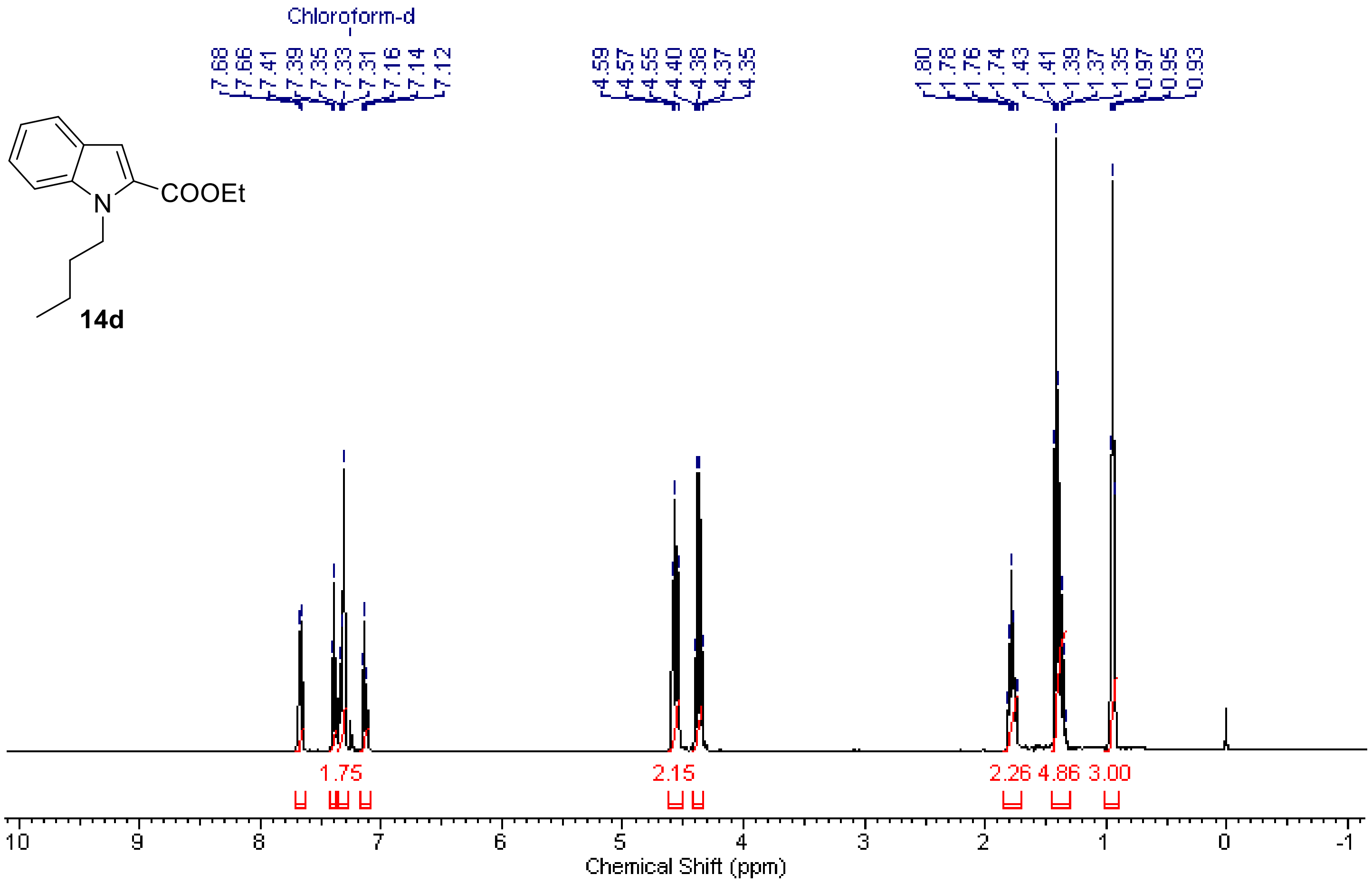

^{13}C NMR spectrum of *ethyl 1-butyl-1H-indole-2-carboxylate* (**14d**)

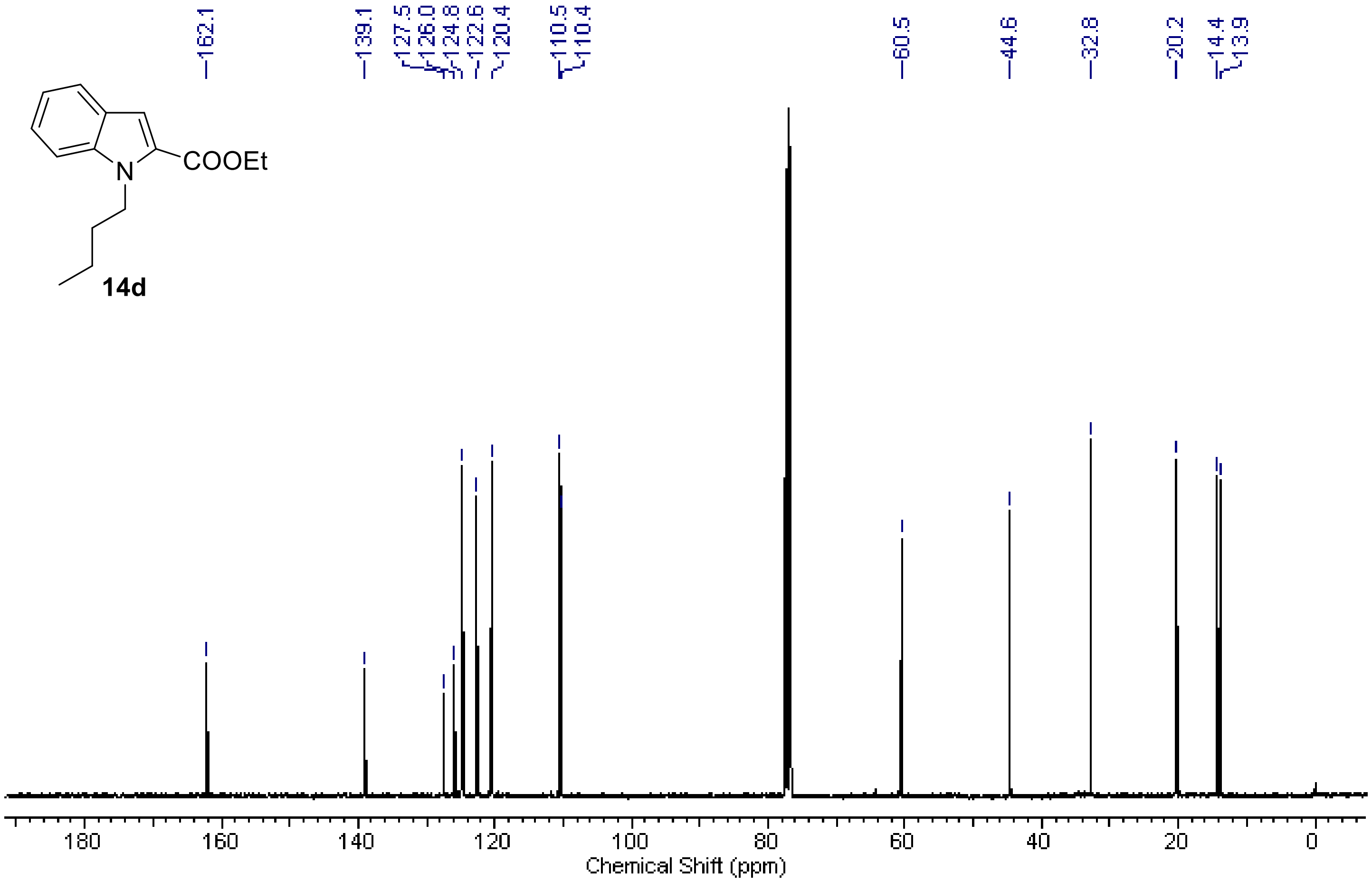

^{1}H NMR spectrum of *ethyl 1-tosyl-1H-indole-2-carboxylate* (**14g**)

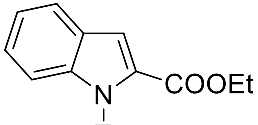

14g

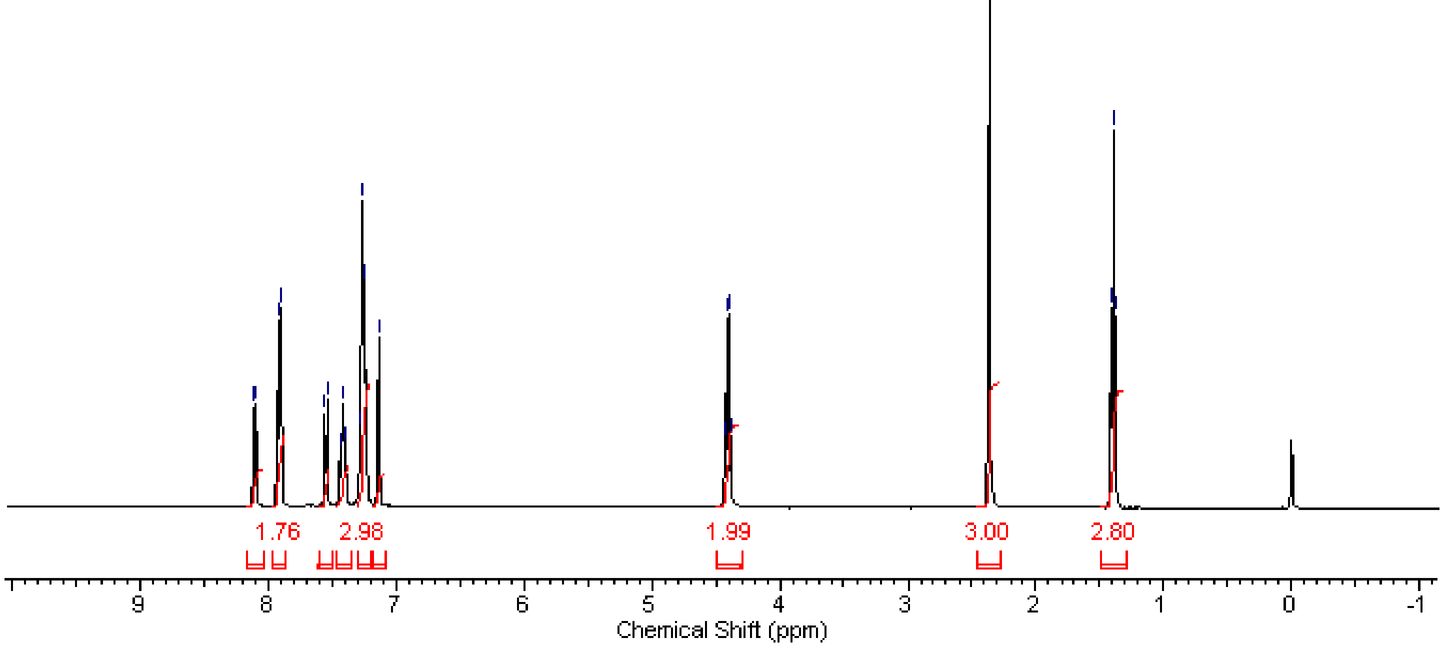

^{13}C NMR spectrum of *ethyl 1-tosyl-1H-indole-2-carboxylate* (**14g**)

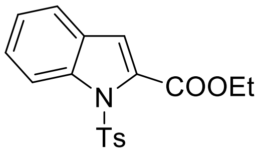

14g

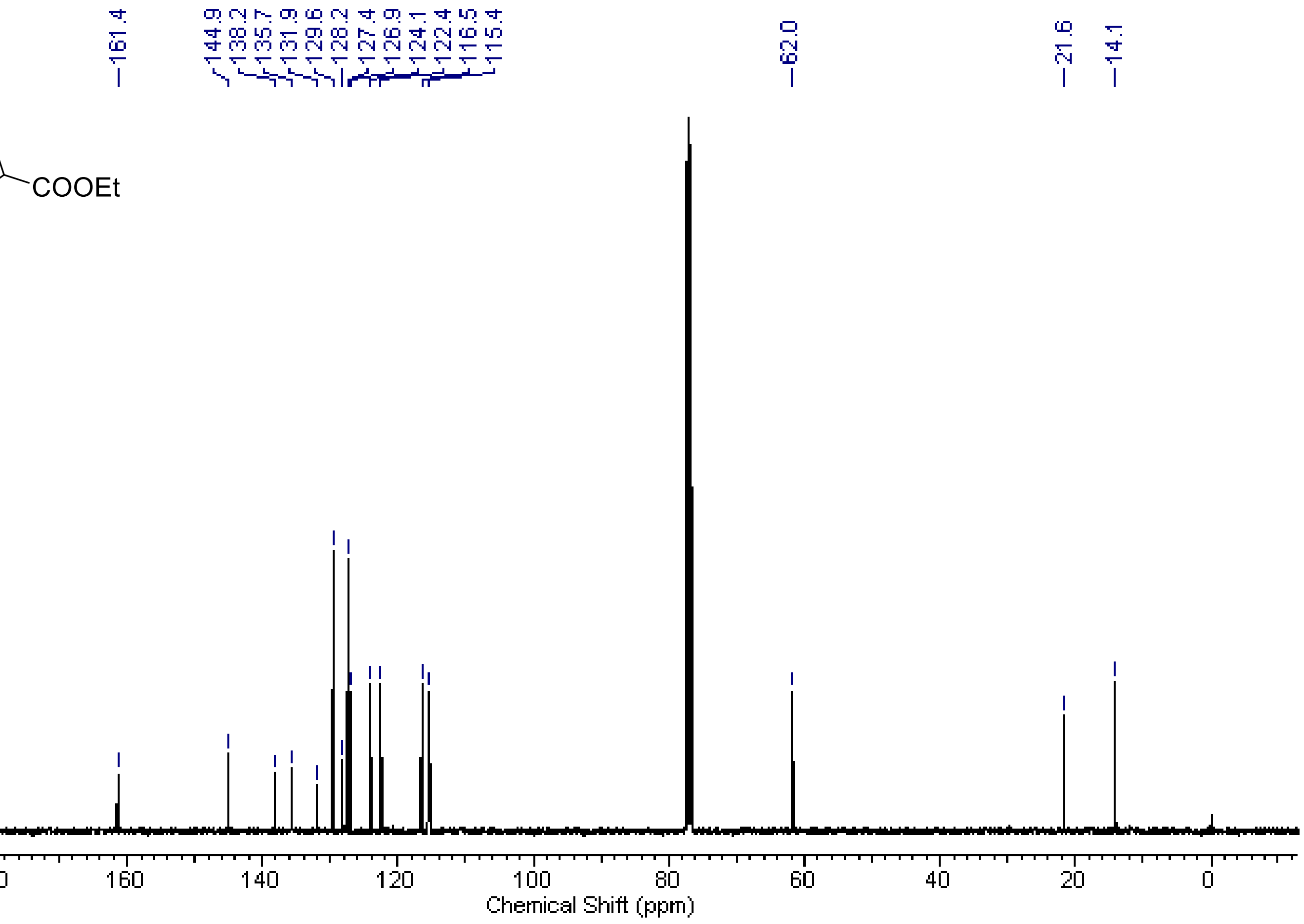

^{1}H NMR spectrum of *1H-indole-2-carbaldehyde* (**15**)

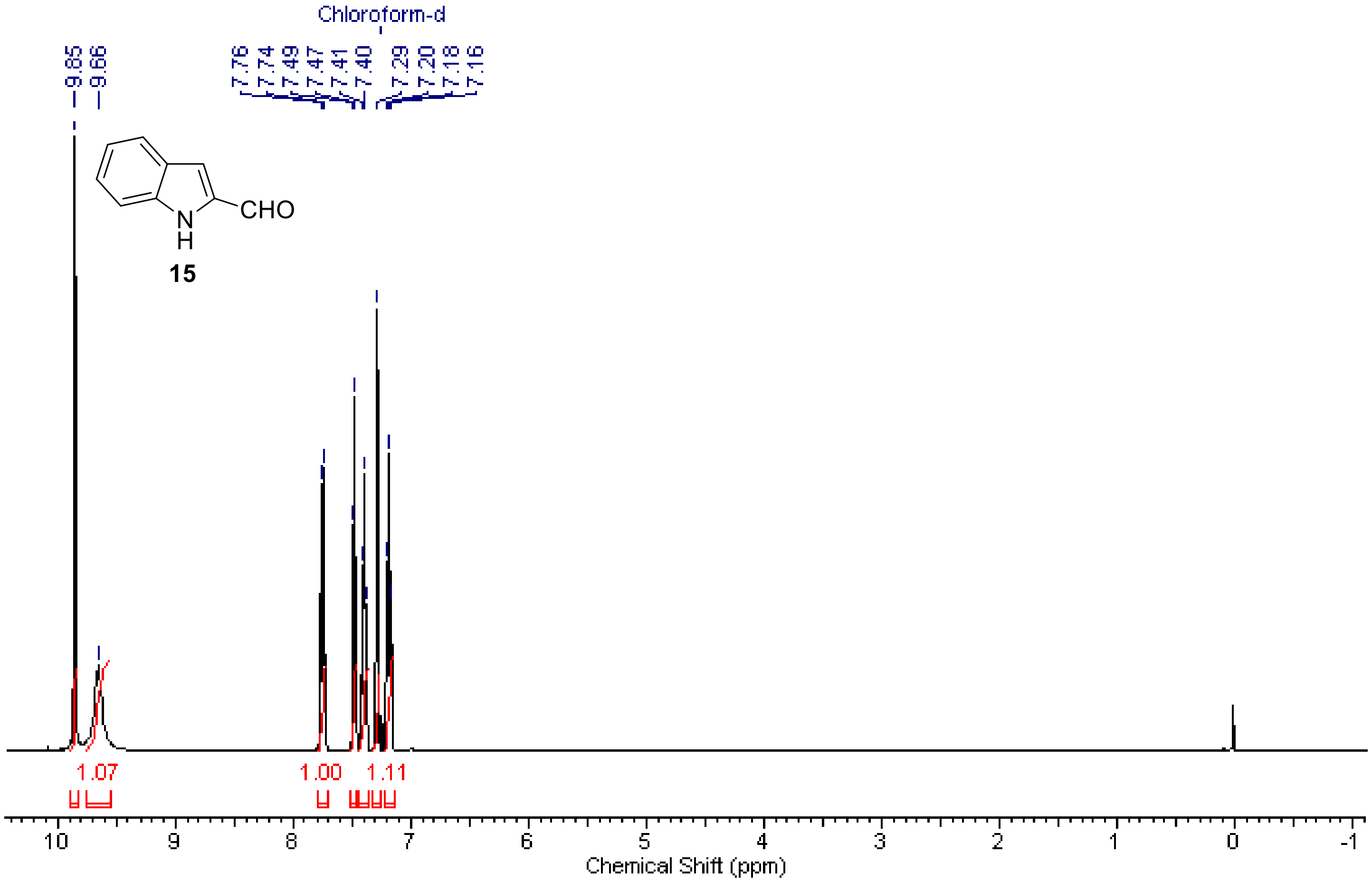

^{13}C NMR spectrum of *1H-indole-2-carbaldehyde* (**15**)

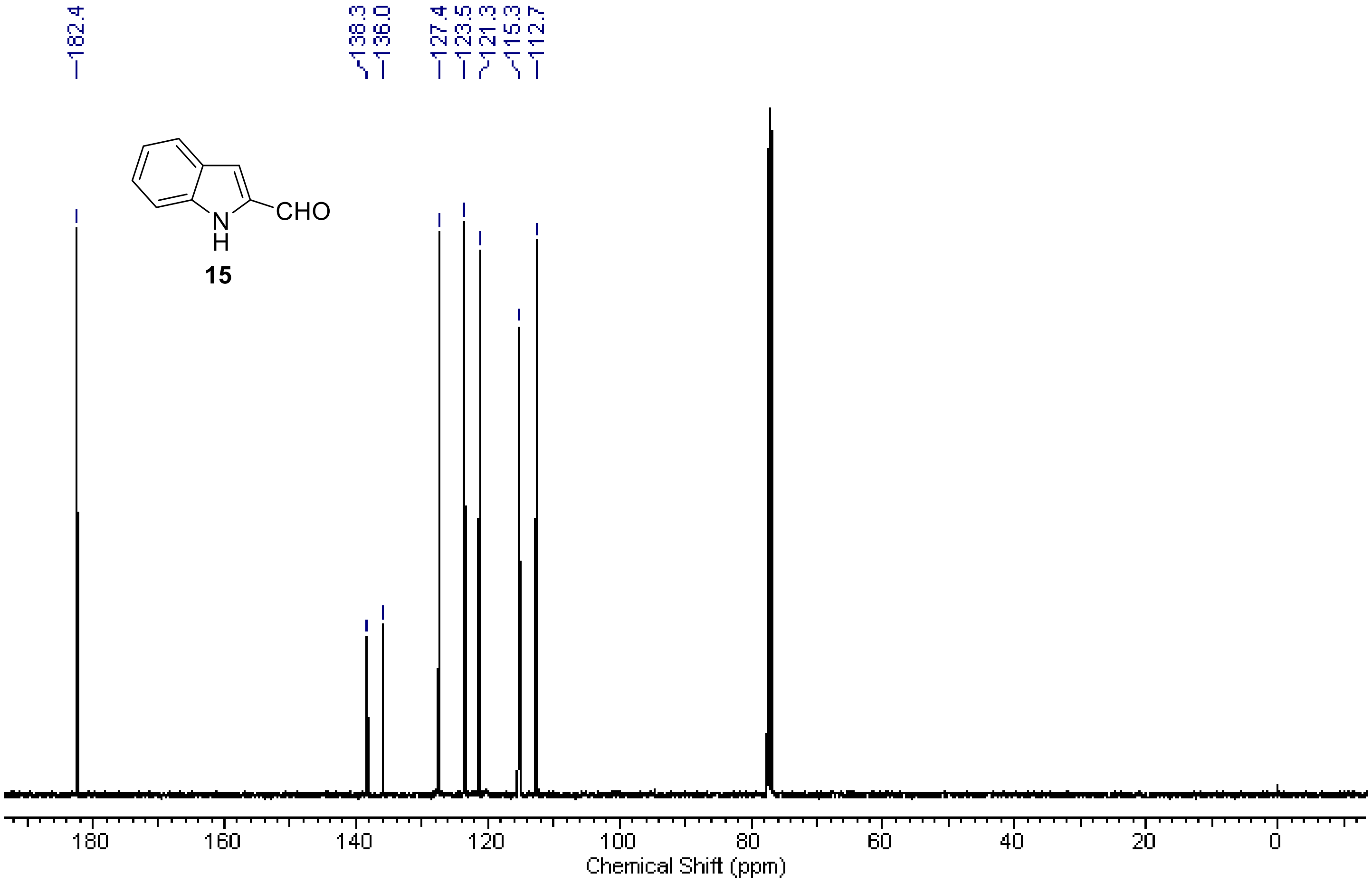

8. References

1. Clerici, F.; Gelmi, M. L.; Rossi, L. M. *Synthesis* **1987**, *11*, 1025–1027.
2. Biswas, S.; Singh, V.; Batra, S. *Tetrahedron* **2010**, *66*, 7781–7786.
3. Meguellati, A.; Ahmed-Belkacem, A.; Yi, W.; Haudecoeur, R.; Crouillère, M.; Brillet, R.; Pawlotsky, J. M.; Boumendjel, A.; Peuchmaur, M. *Eur. J. Med. Chem.* **2014**, *80*, 579–592.
4. Karadeolian, A.; Kerr, M. A. *Angew. Chem. Int. Ed.* **2010**, *49*, 1133–1135.
5. Carlier, P. R.; Lam, P. C. H.; Wong, D. M. *J. Org. Chem.* **2002**, *67*, 6256–6259.
6. Santaniello, E. *Synthesis* **1979**, *8*, 617–618.
7. Kiguchi, T.; Kuninobu, N.; Takahashi, Y.; Yoshida, Y.; Naito, T. *Synthesis* **1989,** *10*, 778–781.
8. Jiang, X.; Tiwari, A.; Thompson, M.; Chen, Z.; Cleary, T. P.; Lee, T. B. *Org. Process Res. Dev.* **2001**, *5*, 604–608
9. Quasdorf, K. W.; Riener, M.; Petrova, K. V.; Garg, N. K. *J. Am. Chem. Soc.* **2009**, *131*, 17748–17749.
10. Nowacki, M.; Wojciechowski, K. *Beilstein J. Org. Chem.* **2018**, *14*, 194–202.
11. McNulty, J.; Keskar, K.; Bordón, C.; Yolken, R.; Jones-Brando, L. *Chem. Commun.* **2014**, *50*, 8904–8907.
12. Pérez-Serrano, L.; Casarrubios, L.; Dominguez, G.; González-Pérez, P.; Pérez-Castells, J. *Synthesis* **2002**, 13, 1810–1812.
